# Unique features of a global human ectoparasite identified through sequencing of the bed bug genome

**DOI:** 10.1038/ncomms10165

**Published:** 2016-02-02

**Authors:** Joshua B. Benoit, Zach N. Adelman, Klaus Reinhardt, Amanda Dolan, Monica Poelchau, Emily C. Jennings, Elise M. Szuter, Richard W. Hagan, Hemant Gujar, Jayendra Nath Shukla, Fang Zhu, M. Mohan, David R. Nelson, Andrew J. Rosendale, Christian Derst, Valentina Resnik, Sebastian Wernig, Pamela Menegazzi, Christian Wegener, Nicolai Peschel, Jacob M. Hendershot, Wolfgang Blenau, Reinhard Predel, Paul R. Johnston, Panagiotis Ioannidis, Robert M. Waterhouse, Ralf Nauen, Corinna Schorn, Mark-Christoph Ott, Frank Maiwald, J. Spencer Johnston, Ameya D. Gondhalekar, Michael E. Scharf, Brittany F. Peterson, Kapil R. Raje, Benjamin A. Hottel, David Armisén, Antonin Jean Johan Crumière, Peter Nagui Refki, Maria Emilia Santos, Essia Sghaier, Sèverine Viala, Abderrahman Khila, Seung-Joon Ahn, Christopher Childers, Chien-Yueh Lee, Han Lin, Daniel S. T. Hughes, Elizabeth J. Duncan, Shwetha C. Murali, Jiaxin Qu, Shannon Dugan, Sandra L. Lee, Hsu Chao, Huyen Dinh, Yi Han, Harshavardhan Doddapaneni, Kim C. Worley, Donna M. Muzny, David Wheeler, Kristen A. Panfilio, Iris M. Vargas Jentzsch, Edward L. Vargo, Warren Booth, Markus Friedrich, Matthew T. Weirauch, Michelle A. E. Anderson, Jeffery W. Jones, Omprakash Mittapalli, Chaoyang Zhao, Jing-Jiang Zhou, Jay D. Evans, Geoffrey M. Attardo, Hugh M. Robertson, Evgeny M. Zdobnov, Jose M. C. Ribeiro, Richard A. Gibbs, John H. Werren, Subba R. Palli, Coby Schal, Stephen Richards

**Affiliations:** 1 Department of Biological Sciences, University of Cincinnati, Cincinnati, Ohio 45221, USA; 2 Fralin Life Science Institute and Department of Entomology, Virginia Tech, Blacksburg, Virginia 24061, USA; 3 Department of Biology, Applied Zoology, Technische Universitaet Dresden, Dresden 01062, Germany; 4 Department of Biology, University of Rochester, Rochester, New York 14627, USA; 5 National Agricultural Library, Beltsville, Maryland 20705, USA; 6 Department of Entomology, University of Kentucky, Lexington, Kentucky 40546, USA; 7 Department of Entomology, Washington State University, Pullman, Washington 99164, USA; 8 ICAR-National Bureau of Agricultural Insect Resources, Indian Council of Agricultural Research, Bengaluru 560024, India; 9 Department of Microbiology, Immunology, and Biochemistry, University of Tennessee Health Sciences Center, Memphis, Tennessee 38163, USA; 10 Cologne Biocenter and Zoological Institute, University of Cologne, Cologne 50674, Germany; 11 Institut für Bienenkunde (Polytechnische Gesellschaft), Goethe University Frankfurt, Oberursel 61440, Germany; 12 Department of Neurobiology and Genetics, Theodor-Boveri-Institute, Biocenter, University of Würzburg, Würzburg 97074, Germany; 13 Department of Evolutionary Biology, Institute of Biology, Freie Universitaet, Berlin 14195, Germany; 14 Department of Entomology, Texas A&M University, College Station, Texas 77843, USA; 15 Department of Genetic Medicine and Development and Swiss Institute of Bioinformatics, University of Geneva, Geneva 1211, Switzerland; 16 Computer Science and Artificial Intelligence Laboratory, Massachusetts Institute of Technology and The Broad Institute of MIT and Harvard, Cambridge, Massachusetts 02139, USA; 17 Pest Control Biology and Research Technologies, Bayer CropScience AG, Monheim 40789, Germany; 18 Department of Entomology, Purdue University, West Lafayette, Indiana 47907, USA; 19 Department of Entomology and Nematology, University of Florida, Gainesville, Florida 32611, USA; 20 Institue de Génomique Fonctionnelle de Lyon (IGFL), Ecole Normale Supérieure de Lyon, UMR5242-CNRS, Lyon 69007, France; 21 Department of Entomology, Max Planck Institute for Chemical Ecology, Jena 07745, Germany; 22 Graduate Institute of Biomedical Electronics and Bioinformatics, National Taiwan University, Taipei 10617, Taiwan; 23 Human Genome Sequencing Center, Department of Human and Molecular Genetics, Baylor College of Medicine, Houston, Texas 77030, USA; 24 Department of Biochemistry and Genetics Otago, University of Otago, Dunedin 9054, New Zealand; 25 Institute of Fundamental Science, Massey University, Palmerston North 4442, New Zealand; 26 Institute for Developmental Biology, University of Cologne, Cologne 50674, Germany; 27 Department of Biological Sciences, University of Tulsa, Tulsa, Oklahoma 74104, USA; 28 Department of Biological Sciences, Wayne State University, Detroit, Michigan 48202, USA; 29 Center for Autoimmune Genomics and Etiology, Division of Biomedical Informatics, and Division of Developmental Biology, Cincinnati Children's Hospital Medical Center, Department of Pediatrics, College of Medicine, University of Cincinnati, Cincinnati, Ohio 45229, USA; 30 Department of Entomology, The Ohio State University, Wooster, Ohio 44691, USA; 31 Department of Biological Chemistry and Crop Protection, Rothamsted Research, BBSRC Harpenden, Herts AL5 2JQ, UK; 32 United States Department of Agriculture—Agricultural Research Service Bee Research Laboratory, Beltsville, Maryland 20705, USA; 33 Department of Epidemiology of Microbial Diseases, Yale School of Public Health, Yale University, New Haven, Connecticut 06520, USA; 34 Department of Entomology, University of Illinois at Urbana-Champaign, Urbana, Illinois 61801, USA; 35 Laboratory of Malaria and Vector Research, National Institute of Allergy and Infectious Disease, Bethesda, Maryland 20892, USA; 36 Department of Entomology and W.M. Keck Center for Behavioral Biology, North Carolina State University, Raleigh, North Carolina 27695, USA

## Abstract

The bed bug, *Cimex lectularius*, has re-established itself as a ubiquitous human ectoparasite throughout much of the world during the past two decades. This global resurgence is likely linked to increased international travel and commerce in addition to widespread insecticide resistance. Analyses of the *C. lectularius* sequenced genome (650 Mb) and 14,220 predicted protein-coding genes provide a comprehensive representation of genes that are linked to traumatic insemination, a reduced chemosensory repertoire of genes related to obligate hematophagy, host–symbiont interactions, and several mechanisms of insecticide resistance. In addition, we document the presence of multiple putative lateral gene transfer events. Genome sequencing and annotation establish a solid foundation for future research on mechanisms of insecticide resistance, human–bed bug and symbiont–bed bug associations, and unique features of bed bug biology that contribute to the unprecedented success of *C. lectularius* as a human ectoparasite.

## Abstract (web summary)

The bed bug, *Cimex lectularius*, is a ubiquitous human ectoparasite with global distribution. Here, the authors sequence the genome of the bed bug and identify reductions in chemosensory genes, expansion of genes associated with blood digestion and genes linked to pesticide resistance.

## Graphical abstract

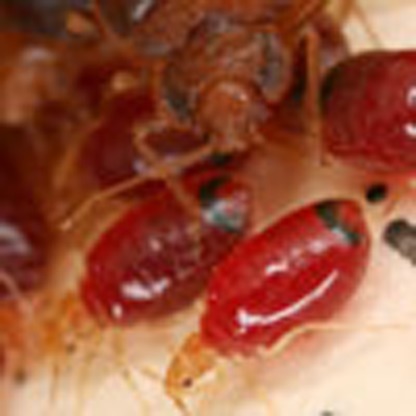

The common bed bug, *Cimex lectularius*, has a 3,000-year documented association with humans that is likely much more ancient^1^. This species was nearly eradicated after World War II in most economically and politically stable countries, in part through the liberal use of pesticides^2^, but reservoir populations have remained in underdeveloped countries, disadvantaged communities and in association with bats, chickens and other animals^2^. During the past ∼20 years, however, there has been a global resurgence of bed bugs in every continent except Antarctica^2^. The upsurge in prevalence of bed bugs has been extraordinary with infestations increasing 4,500% in Australia and similar escalations in other regions^2^,^3^. Bed bugs have also become highly prolific in the United States, with reports of infestations in all 50 states^2^,^3^. This rapid expansion has been linked to increased international travel, frequent exchange of second-hand materials, a lack of education on issues related to bed bugs and the evolution of resistance to all major classes of insecticides, including organochlorines, organophosphates and pyrethroids^4^.

The biology of bed bugs features many unique aspects that contribute to their success as a human parasite^5^. First, all mobile life stages of the bed bug are obligatory blood feeders, and blood serves as the sole source for ingested nutrients and water^5^,^6^. This trophic specialization requires a dedicated chemosensory system to detect, find and accept proper hosts. Moreover, recent evidence of two distinct lineages of *C. lectularius*, one associated with humans and the other with bats^7^, suggests chemosensory specialization between bat- and human-associated bed bugs. In addition, hematophagy requires specific enzymes and associated pathways to properly digest and assimilate blood and dispose of excess water; such specialization also drives obligate associations with symbionts, including *Wolbachia*, that generate critical micronutrients that are deficient in vertebrate blood^8^,^9^.

Bed bugs mate through traumatic insemination; males pierce the cuticle of the female abdomen with a modified reproductive organ and deliver sperm into her haemolymph^10^,^11^. This mode of reproduction is under strong selection by sexual conflict that involves sexually transmitted microbes and selects for immune networks that may in turn affect bed bug–pathogen associations^12^. Bed bugs appear resistant to the effects of repeated rounds of inbreeding^13^, which likely results in the fixation of beneficial gene complexes and the purging of deleterious alleles that may improve local adaptation, such as pesticide resistance. Interestingly, outbreeding does not appear to be disadvantageous, but any heterotic effect appears to be minimal and short-lived^13^. Bed bugs have recently been identified as potential vectors for American trypanosomes^14^, but unlike other haematophagous arthropods, direct confirmation of pathogen transmission to humans is rare^15^,^16^. Last, resistance to insecticides has become widespread and pyrethroid resistance has reached levels 10,000-fold higher than in susceptible bed bug populations^17^. Multiple mechanisms of insecticide resistance and cross-resistance may also impede the development of new classes of pesticides. Recent transcriptome studies have examined other specific aspects of bed bug biology, but the lack of a sequenced genome has stalled deeper understanding of bed bugs and their evolutionary and ecological relationships relative to other insects.

Here we report the genome of *C*. *lectularius* and associated bacteria along with phylogenomic analyses and extensive manual annotation. This study reveals evolutionary adaptations associated with the lifestyle of bed bugs, including significant reductions in chemosensory genes, expansion of genes that are associated with blood digestion and the entire repertoire of genes that have been associated with pesticide resistance in various other species. In addition, we identified the presence of multiple putative lateral gene transfer (LGT) events from various bacteria, including *Wolbachia* and *Arsenophonus*.

## Results and Discussion

### General features of the genome and orthologue analyses

Individual features of the bed bug genome analyses are provided as Supplementary Information (Supplementary Figs 1–42; Supplementary Notes 1–22; Supplementary Data 1–33). Our final draft assembly comprises 650.47 Mb of total sequence in 1,402 scaffolds and 45,073 contigs (N50 lengths 7.17 Mb and 23.5 kb, respectively; Supplementary Data 1). This is 25% smaller than the predicted genome size of 864.5 Mb (determined through comparison with other insects by propidium iodide analyses; Supplementary Note 17) and is likely due to unassembled heterochromatin and other repetitive regions. We predicted 13,953 genes using a custom MAKER annotation pipeline tuned for arthropod genomes and this was improved to 14,220 through manual curation. A total of 1,352 gene models representing gene families of interest, including 273 cuticle proteins and 114 chemoreceptors were manually curated, confirming gene identity and revealing where automated gene structures needed correction (Supplementary Note 1). To assess the completeness of the assembly and gene prediction, we analysed the predicted genes and genome assembly for benchmarking sets of universal single-copy orthologues (BUSCOs^18^). In addition, the presence of a complete Hox cluster and all expected autophagy genes was documented, two categories that are known to be conserved among insect genomes (Supplementary Notes 10 and 12). In general, the *C*. *lectularius* gene set and genome has slightly more missing BUSCOs, ∼10%, compared with the genomes of seven other arthropods, but is still relatively complete (Supplementary Data 28). We therefore concluded that the data set for *C*. *lectularius* is sufficiently comprehensive for further downstream analyses.

**Figure 1:**
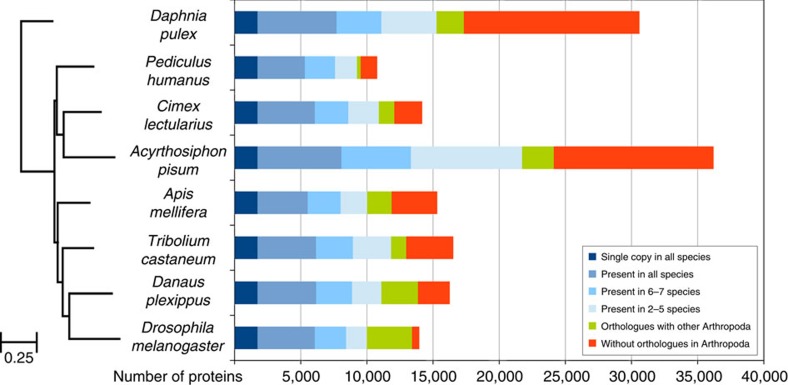
Phylogenetic placement and orthology comparison among *Cimex lectularius* and other arthropod species. The phylogenetic analysis places *C. lectularius* as a sister species to another hemipteran, *Acyrthosiphon pisum*. The phylogeny is built using RAxML and it is based on the 1,734 single-copy orthologues that are present in all eight species. All nodes in the phylogenetic tree have 100% bootstrap support, while the branch length unit is substitutions per site. There are 1,734 genes that are present as single copy in all eight species tested. Another 4,187 of the *C. lectularius* genes are found in varying copy number in the other seven species, while 2,433 are found in the majority of species (that is, in 5–7 species) and 2,153 genes are found in ≥2 species (that is, in 2–4 species). Moreover, 1,147 genes have an orthologue in an arthropod other than the selected seven species. Last, 2,285 genes are lineage specific and do not have an orthologue in any other arthropod species.

In addition, we characterized homologous and orthologous relationships between genes in relation to those of other sequenced arthropods using a previously described orthology delineation approach employed by OrthoDB^19^. The analyses were performed with the 45 arthropod species included in OrthoDB7 (http://www.orthodb.org). Over 80% of *C. lectularius* genes have orthologues in at least one arthropod species (Fig. 1). Of these, 1,734 were universal single-copy orthologues across eight species, which were used to determine the maximum-likelihood phylogeny. As expected, our analyses of these eight arthropod genomes placed another hemipteran, the pea aphid *Acyrthosiphon pisum*, as the sister species of *C*. *lectularius* (Fig. 1). It is worth noting that *A*. *pisum* has more than twice as many genes due to extensive gene duplication in >2,000 gene families^20^. Large-scale transcription factor analyses revealed 634 putative transcription factors and we were able to infer DNA-binding motifs for 214 (34%; Supplementary Note 22).

### Host location, obligate blood feeding and immunity

Bed bugs are obligate blood feeders, and unlike mosquitoes and many other blood-feeding insects, all immature stages and both sexes of adults rely exclusively on blood for nutrition and water^5^,^6^,^7^. *C. lectularius* prefers humans as hosts but accepts a range of other vertebrate hosts^5^,^7^. The association with humans in the built environment, coupled with their crepuscular/nocturnal activity and the complete reduction in wings, predicts specialized mechanisms for host location, acceptance, and blood ingestion and digestion. Bed bugs are equipped with small compound eyes that protrude prominently from the lateral head capsule and object recognition is suspected to play a role in host detection^21^. Consistent with low-resolution landscape vision, the bed bug genome contains one member each of the ultraviolet- and broadband long-wavelength-sensitive rhabdomeric opsin subfamilies in line with that of most other hemipteran genomes sequenced (Supplementary Note 9), as well as crepuscular insect species in general^22^. Circadian clock genes in *C. lectularius* appear to encode both *Drosophila*- and mammalian-like proteins (Supplementary Note 5), with notable absence of sequences for CRY1 and JET, which are necessary in *Drosophila* for the light input pathway to the clock^23^,^24^. *Cimex* may thus represent a valuable model in circadian rhythm research, particularly for organisms that inhabit low-light or -dark environments.

**Figure 2:**
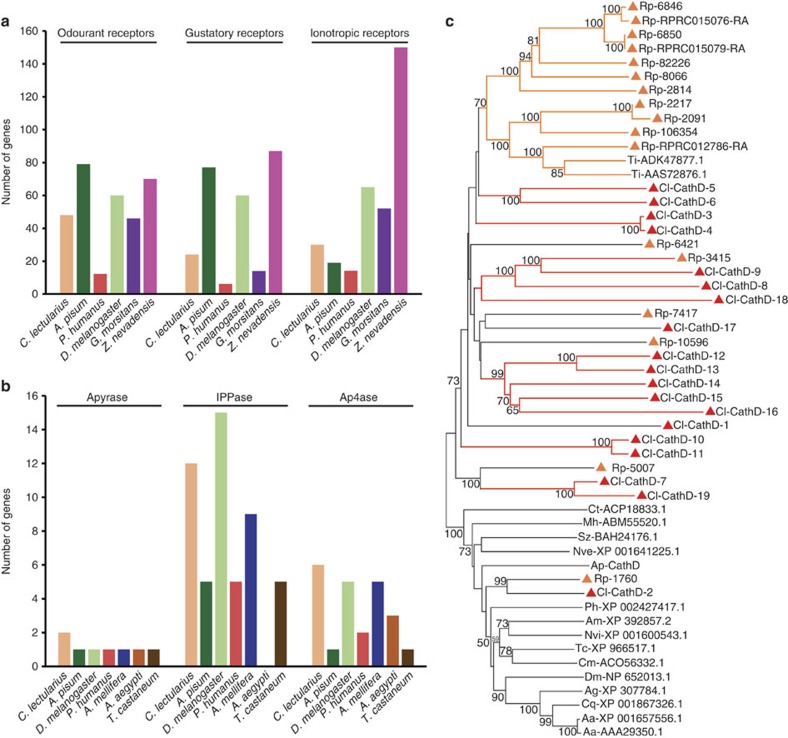
Aspects related to host location and blood feeding identified based on the *C*. *lectularius* genome. (**a**) Genes associated with chemical reception among multiple insect species. *Zootermopsis nevadensis* (**b**) Genes associated with saliva function among multiple insect species. (**c**) Phylogeny of cathepsin D genes among multiple insect species. Sequences derived from *Cimex lectularius* (Cl) are denoted with red triangles and those derived from *Rhodnius prolixus* (Rp) are denoted with orange triangles. Other insect cathepsin D proteins represent those of *Triatoma infestans* (Ti), *Acyrthosiphon pisum* (Ap), *Anopheles gambiae* (Ag), *Drosophila melanogaster* (Dm), *Pediculus humanus corporis* (Ph), *Apis mellifera* (Am), *Nasonia vitripennis* (Nvi), *Tribolium castaneum* (Tc), *Callosobruchus maculatus* (Cm), *Sitophilus zeamais* (Sz), *Chrysomela tremula* (Ct), *Maconellicoccus hirsutus* (Mh), *Nematostella vectensis* (Nve), *Culex quinquefasciatus* (Cq) and *Aedes aegypti* (Aa).

Olfactory and gustatory processing in insect sensilla depends on three families of chemoreceptors: odourant, gustatory and ionotropic receptors^25^. Olfactory receptors play critical roles in mate finding, host location and navigation through a dark environment using the sense of smell. The major functions of gustatory receptors (GRs) are to mediate gustation—most importantly to detect sweet (phagostimulatory) and bitter (deterrent) tastants—as well as to sense carbon dioxide^26^. Ionotropic receptors evolved from ionotropic glutamate receptors in ancestral animals, and are involved in both olfaction and gustation^27^. We idenified 48 genes encoding 49 olfactory receptors, 24 genes encoding 36 GRs and 30 ionotropic receptor genes (Fig. 2a; Supplementary Note 4). This repertoire of chemosensory genes is substantially reduced relative to that of phytophagous hemipterans (for example, pea aphid), extending a similar trend noted in the genome sequences of other blood-feeding insects (Fig. 2a). Moreover, the intermediately sized repertoire of bed bug chemoreceptors is in line with the moderate complexity of its chemical ecology, being an obligate blood feeder such as tsetse flies (*Glossina morsitans*)^28^,^29^, but having a broader host range that encompasses many vertebrates, whereas *Pediculus humanus humanus* (body louse) feeds only on humans^30^. We found no sugar receptors in the *Cimex* genome, as previously documented in other obligate blood feeders, including tsetse flies^28^,^29^ and lice^30^. This finding also explains the lack of phagostimulation by glucose in *C. lectularius*^31^. Remarkably, *Cimex* has four GRs related to a conserved lineage of carbon dioxide receptors found in flies, moths, beetles and a termite^32^, but that are absent from the pea aphid, hymenopteran species and blood feeders such as *Pediculus*^30^ (Supplementary Note 4). The *Cimex* chemosensory gene families appear to have few expansions and slow evolving members (Supplementary Figs 2–4), suggesting a comparatively stable chemosensory ecology. We also found 11 odourant-binding proteins that appear to be highly species specific in *C. lectularius*, and 14 chemosensory proteins that are more conserved relative to other blood-feeding insects^33^.

One of the major obstacles in the acquisition of a blood meal is host haemostasis, the physiological process that prevents blood loss through platelet aggregation, fibrin crosslinking, vasoconstriction and local immune responses. The bed bug genome builds upon previous sialotranscriptome and proteome studies^34^ and contributes to our understanding of bed bug saliva complexity and unique adaptations of blood-sucking insects. Of interest, *Cimex* appears to have expanded salivary apyrases, proteins involved in the inhibition of ADP-dependent platelet aggregation, including two *Cimex*-type apyrases^35^. In addition, 12 members of the inositol polyphosphate phosphatase family that act as nitric oxide carriers, and 6 members of the Ap4a_hydrolase family, the largest number in any insect genome, were identified (Fig. 2b; Supplementary Note 18). This expanded array of salivary proteins likely permits bed bugs to stealthily feed repeatedly on the same host without inflicting pain.

Vertebrate blood is an excellent source of proteins and lipids, but it is deficient in specific micronutrients, has high water content, and its digestion requires a suite of specific digestive enzymes (Fig. 2c). Analysis of the *C. lectularius* genome revealed 187 potential digestive enzymes (Supplementary Data 12). *C. lectularius* has fewer serine proteases than most insects, but a similar repertoire to blood-feeding *Rhodnius* (kissing bug) and *Pediculus* (Supplementary Note 7 (refs 30, 36)). Of interest is a large expansion of genes associated with cathepsin D (Fig. 2c), an aspartic protease adapted for acidic pH^35^. A similar expansion, albeit of different specific cathepsin D genes, has been found in *Rhodnius* and deemed critical for optimal blood digestion^36^,^37^.

Removal of excess water from the blood meal is essential for proper digestion and aquaporins (AQPs) appear to be critical for this process^38^. Bed bugs possess seven or eight AQP genes, which are within the 6–8 range common for most insects^38^ (Supplementary Note 3). Among peptide hormones and amine receptors, we documented a full complement of diuretic and antidiuretic hormones and their receptors that serve to precisely initiate and terminate postprandial diuresis (Supplementary Note 14). Unlike *Rhodnius*^39^,^40^, *C. lectularius* has only one *capa* gene encoding antidiuretic neuropeptide hormone.

Like blood-feeding ticks and triatomine bugs, but unlike most other blood-feeding insects, bed bugs can survive long periods of starvation between blood meals^5^,^6^. This adaptation requires nutrient conservation (for example, lower metabolism) and mechanisms to prevent excessive water loss and dehydration-induced mortality^5^,^6^. The latter is specifically dependent on differential expression of aquaporins and heat-shock proteins^38^ (Supplementary Notes 3 and 11). In general, genes for heat-shock proteins and autophagy are similar in *Cimex* and other insect species, suggesting that their differential expression is likely responsible for the extreme dehydration and starvation tolerance noted in bed bugs. These gene sequences will facilitate the discovery of other physiological and behavioural mechanisms underlying the extreme dehydration and starvation tolerance of bed bugs.

The bed bug genome shows strong candidates for the key members of the Toll, Imd and Jak/STAT immune pathways, although the *C*. *lectularius* repertoire is arguably more sparse for those pathways than in holometabolous insects^41^. Recognition proteins are under-represented, as are antimicrobial peptides (two recognition proteins and two defensins and a cluster of three diptericin-like peptides; Supplementary Data 29), although the latter are notoriously difficult to identify by sequence similarity. The RNA interference pathway is represented in the *C. lectularius* genome with multiple paralogues for Dicer, Argonaute and other enzymes required for this defence pathway.

### Symbiosis and lateral gene transfer

Obligate hematophagy can result in significant deficiencies in specific micronutrients that are poorly represented in blood. *Wolbachia*, a common endoparasite that can affect growth and reproduction in many insect species^42^, has evolved a symbiotic nutritional relationship with *C. lectularius*^8^,^9^. *Wolbachia* provides the bed bug with a cocktail of specific B vitamins that are critical for reproduction and development^8^,^9^. We annotated genes associated with B vitamin metabolism and determined, as with other insects, that bed bugs possess the genes necessary for B vitamin salvage and conversion after their ingestion in blood or synthesis by *Wolbachia* (Supplementary Note 19).

A computational pipeline^43^ was used to detect bacterial scaffolds within the assembly as well as candidate LGTs from bacteria to the bed bug. The nearly complete *Wolbachia* endosymbiont of *C. lectularius* was assembled into 16 scaffolds (Supplementary Data 21). In addition, the nearly complete genome of a *Staphylococcus* associate of bed bugs was assembled into 15 scaffolds, which include three plasmids and a ∼3.16-Mb chromosome (Supplementary Data 21). On the basis of high-sequence similarity of chromosomal scaffolds, this bacterium is a close relative of *S*. *xylosus*, an associate of the skin of humans and other animals^44^. *Staphylococcus* bacteria are commonly found in bed bugs based on two microbiome surveys^12^,^45^, including on the male genitalia and inside the female body, and we report here the first draft genome of this *C. lectularius* associate. There is evidence of sexual transmission of *Staphyloccus*^12^. Further studies are necessary to determine whether this bacterium is routinely acquired from human hosts or is a strain specifically adapted to *Cimex*. A third scaffold was assembled with homology to the bacterium *Pectobacterium carotovorum*. This bacterium is typically associated with plants, and the scaffold is only 250 kb in size, whereas *P. carotovorum* genomes are typically ∼5 Mb. Further, the scaffold does not contain a ribosomal locus, and therefore this bacterium is unlikely to be an endosymbiont of *Cimex* with a severely reduced genome size. Given that coverage of this scaffold is similar to the genome coverage (Supplementary Data 21), we speculate that this may be a large lateral gene insertion in *Cimex*; however, further study is needed to resolve this question.

**Figure 3:**
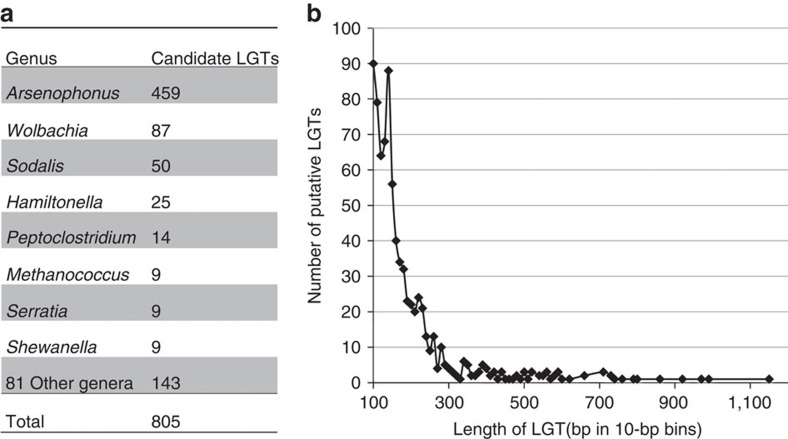
Summary of putative lateral gene transfers (LGTs) >100 bp in the *C. lectularius* assembly. (**a**) Number of candidate LGTs identified. (**b**) Length of candidate LGTs in bins spanning 10 bp.

The bed bug shows evidence of extensive bacterial LGTs in its genome. In addition to the case described above, there are 805 candidate LGTs of size >100 bp that appear to be scattered throughout the bed bug genome (Fig. 3). This is the largest number of candidate LGTs found in screening of 14 arthropod genomes using this pipeline. LGTs from the genus *Arsenophonus* (*n*=459 or 57%) are the most commonly found, followed by *Wolbachia* (*n*=87 or 10.9%). Other genera represented include *Sodalis*, *Hamiltoniella* and *Peptoclostridium*. The large number of *Arsenophonus* LGTs found is uncommon in insect genomes so far screened, with numbers typically ranging from 0–22. *Arsenophonus* is a widely distributed arthropod-associated bacterium^46^, but has not been reported in *Cimex* and no scaffolds for this bacterium were detected in the genome assembly. The type species *A. nasoniae*^47^ has a sequenced genome^48^. It causes male killing in a parasitoid wasp, whereas phenotypic effects of other *Arsenophonus* are less well understood. The second most common source of candidate LGTs in *Cimex* is *Wolbachia*, which is a known mutualistic endosymbiont. *Wolbachia* sequences from this symbiont assembled into a nearly complete genome with high sequence coverage. Because of the presence of *Wolbachia* bacteria in *Cimex*, it is possible that some apparent LGTs are due to assembly errors joining *Wolbachia* and *Cimex* sequences. However, examination of junctions between eukaryotic and prokaryotic sequences for spanning sequence reads and cloned paired ends strongly supports that nearly all of these are legitimate LGT events. In addition, LGT–eukaryotic sequence junctions were amplified for five of six candidates, confirming their presence in the genome (Supplementary Fig. 42). The LGTs found in *Cimex* appear to be unique insertions, as no matches were found to the closest published insect genome (*A. pisum*). Comparative studies among *C. lectularius* populations and related species will be important to determine whether there is genomic variation in LGTs among bed bugs.

The typical pattern of LGT evolution is expected to be insertion (most likely due to non-homologous DNA repair mechanisms) followed by degradation and loss. In this way, LGTs are similar to nuclear mitochondrial DNA insertions found in the genome of most eukaryotes^49^. However, bacterial LGTs can also evolve into functional eukaryotic genes, providing novel biochemical functions in the eukaryote^50^. Most candidate LGTs in *C. lectularius* show no or only traces of gene expression in RNA-seq data from adult males and females, and are thus unlikely to be functional. An exception is a *Wolbachia* LGT on scaffold 132, which encodes a patatin-like gene. Bacterial patatin-like genes have lypolytic properties and can be involved in pathogenicity of some bacteria^51^,^52^. This LGT shows high expression in adult males but no expression was detected in adult females. While the function and detailed male expression pattern of this gene remain to be determined, we speculate that it may be involved in the unusual insemination mechanism of *Cimex*.

### Genes associated with pesticide resistance

A major factor for the increased prevalence of bed bugs in the past two decades, and a contributing factor to the immense difficulties in eradicating infestations, has been the pervasiveness of pyrethroid resistance^4^,^53^,^54^,^55^,^56^. Resistance can result from multiple mechanisms that include target-site mutations, differential gene expression, alterations in the permeability of the cuticle or digestive tract and behavioural changes^4^,^57^,^58^,^59^. Transcriptomic evidence supports the presence of multiple resistance mechanisms in bed bug populations^4^. To fully understand these potential mechanisms, we manually annotated genes associated with pesticide resistance, including cuticular proteins that can impede pesticide penetration and enzymes that can detoxify pesticides.

V419L and L925I mutations in the voltage-gated sodium channel α-subunit gene have been identified and shown to be responsible for deltamethrin (a pyrethroid) resistance in bed bugs^60^. Molecular analysis of bed bug populations from across the USA and Europe found that >80% and >95% of the respective populations contained V419L and/or L925I mutations in the voltage-gated sodium channel gene, indicating widespread distribution of target-site-based pyrethroid resistance^7^,^61^. Previous studies showed that higher expression of genes coding for metabolic enzymes including P450s, carboxylesterases and glutathione-*S*-transferases and a reduction in penetration due to higher expression of cuticular protein genes are likely responsible for insecticide resistance of bed bugs^4^,^59^.

Insect genomes code for four distinct clades of P450s called clans: the CYP2, 3, 4 and Mito clan. The *C*. *lectularius* genome contains 58 genes and one pseudogene coding for P450 enzymes (Supplementary Data 22). Relatively few insect P450s with known or suspected physiological functions are significantly conserved across species^53^, and these tend to be involved in biosynthesis of hydrocarbons that cover the insect exoskeleton and prevent desiccation (CYP4G subfamily^4^). Most bed bug P450s (36/58 genes) are members of the highly diverse CYP3 clan; these genes lack clear orthologous relationships and thus are likely involved in species-specific functions. Several transcriptomic analyses have demonstrated substantial overexpression of some bed bug P450s in a manner that was correlated with metabolic resistance to the pyrethroid insecticide deltamethrin^4^,^54^,^58^. Four of the P450 genes identified in the *Cimex* genome (CYP397A1, CYP398A1, CYP4CM1 and CYP6DN1) are known to be overexpressed in deltamethrin-resistant populations^4^. Knockdown in the expression of these four P450 genes by RNA interference caused a reduction in deltamethrin resistance levels^4^,^62^. In addition, RNA interference of cytochrome P450 reductase, which encodes a co-enzyme required for P450 activity, reduced deltamethrin resistance levels in resistant populations of *Cimex*^62^. These results indicate that P450s play an important role in *Cimex* insecticide resistance.

ATP-binding cassette (ABC) transporters play important roles in the shuttling of a wide variety of substrates including hormones, ions, sugars, amino acids, vitamins, peptides, polysaccharides, lipids and insecticides^63^. In a recent study, the expression of 8 out of 27 contigs coding for ABC transporters was elevated in pesticide-resistant *Cimex* populations relative to susceptible populations^54^. We identified 24 additional ABC transporters (Supplementary Data 23); in total, *Cimex* encodes 51 ABC transporters belonging to all eight known classes. Interestingly, 25 of the 51 transporters belong to ABCG/H class, members of which are known to be involved in transport of xenobiotics^63^. Three of the ABC transporters identified in the *Cimex* genome (ABCG20-3, ABCG23-5 and ABCH-B; previously named ABC8, ABC9, ABC10 and ABC11 based on transcriptome analysis^54^) are overexpressed in the epidermis in 21 field-collected resistant populations relative to susceptible populations^4^. In addition, knockdown of ABCG20-3 (ABC8 and ABC9 are encoded by this gene) reduced deltamethrin resistance^4^. With the complete set of ABC transporter genes, future studies will be able to fully assess their contribution to insecticide resistance.

Carboxylesterases are critical in the metabolic breakdown of insecticides^64^. We identified 30 carboxylesterase genes in the *Cimex* genome (Supplementary Data 24). Half of them are located in a single cluster in scaffold 81, suggesting significant gene duplication, and one carboxylesterase, CLE11776 (previously named ClC21331), is expressed at very high levels in most of the 21 field-collected populations tested^4^. We also identified 12 glutathione-*S*-transferase genes in the *Cimex* genome, which was similar to the number identified previously by transcriptome studies^58^ (Supplementary Data 25).

The bed bug cuticle plays a substantial role in resistance to insecticides; this is thought to be due (at least in part) to changes in the expression of cuticle proteins in resistant strains^4^,^54^,^59^. Using the criteria established by Willis^65^, we identified 273 genes that encode putative cuticle proteins (Supplementary Note 6). Of these, 169 genes could be placed in one of eight families (CPR, CPRL, CPF, CPFL, CPAP1, CPAP3, TWD and Dumpy), with an additional 104 proteins consisting of repeated low-complexity sequences (AAPV/GGY) commonly associated with cuticle proteins but without a defining conserved domain (Supplementary Data 10). Approximately, 70% of bed bug cuticle protein genes were arranged in clusters ranging from 3 to 19 genes (Supplementary Data 11; Supplementary Fig. 6); clusters were largely type specific and emphasize the potential for regulatory changes that might influence the expression of the entire cluster.

As in other insects, the CPR family represents the largest single family of putative cuticle protein genes found in the bed bug genome. The 121 CPR-type genes we identified (Supplementary Fig. 7) are slightly more than in *Drosophila*^66^ but fewer than in the silkworm *Bombyx mori*^67^ or the malaria mosquito *Anopheles gambiae*^68^. We note a bed bug-specific expansion in this family consisting of a novel 10 gene cluster whose members encode two chitin-binding domains each; similar gene structures were not identified in the pea aphid or any of the dipteran genomes.

### Traumatic insemination

Among the >40 independent evolutionary events in different lineages leading to traumatic mating, bed bugs are among the best-studied cases^69^. Females evolved a novel organ that reduces the physical trauma of copulation by means of a dense aggregation of the super-elastic protein resilin^70^. Intriguingly, our genome analysis revealed a recent expansion in pro-resilin genes, with 13 such genes containing the pro-resilin characteristics of a chitin-binding domain and consisting of >20% glycine. The pro-resilin gene CPR57 is over 600 amino acids with >40% glycine (Supplementary Figs 7,8). A similar diversification of the resilin gene family is not seen in the related pea aphid (six genes even though there are a similar number of CPR-type cuticle proteins, ∼115 (refs 65, 66)) nor is it seen in other blood-sucking insects that experience the enormous stretching of the cuticle to accommodate the blood meal (*Aedes*, *Pediculus*, and *Anopheles*; 2–6 genes), indicating lineage-specific adaptive significance of the resilin gene family.

### Conclusions

**Figure 4:**
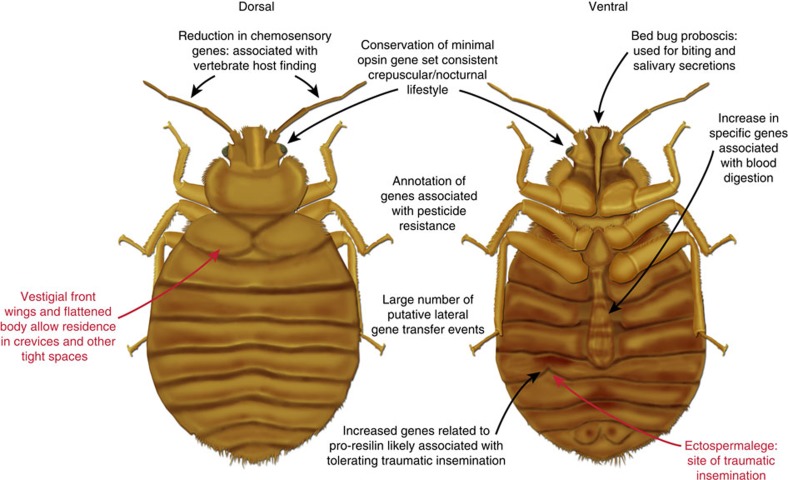
Synopsis of the contributions from the *C*. *lectularius* genome to understanding key biological processes. Red, general characteristics of bed bugs; black, key aspects identified and expanded by genome sequencing and manual curation.

The sequencing, assembly, annotation and manual analyses of the *C*. *lectularius* genome provide an important and timely resource for understanding the biology of this human ectoparasite, as summarized in Fig. 4. It also will serve as a gateway for the discovery of new targets for control of bed bug populations. This reference genome sequence is of a bed bug strain that is common in laboratory cultures and collected before the introduction of pyrethroid insecticides. What triggered the current bed bug resurgence, and did bed bugs originate from one or multiple sources? This genome sequence will facilitate the discovery of molecular markers and single-nucleotide polymorphisms that will enable research to address these questions. There are many related *Cimex* species that specialize on non-human vertebrate hosts. Comparative genomic studies should reveal specific chemosensory and digestive specializations that define anthropophagy in *C. lectularius*. Even host-associated differentiation within this species requires further genomic studies to understand why one lineage of *C. lectularius* prefers humans and another lineage prefers bats, and how the two remain genetically differentiated even within the same home.

Traumatic insemination has evolved multiple times in various unrelated taxa. The sequenced bed bug genome will serve as an important resource for studies on male-expressed gene networks that ensure sperm transfer despite the female’s immune response and other female-expressed pathways that may facilitate cryptic choice of mates. Most haematophagous arthropods have been implicated as vectors of human or animal pathogens, but bed bugs have not. Pathogenic organisms have been isolated from bed bugs, and bed bugs have been shown experimentally to be competent vectors, for example, of American trypanosomes. However, no evidence exists of disease transmission by bed bugs in the field. The sequenced genome will enable studies on mechanisms that actively hinder or do not support vertebrate pathogen survival, proliferation and transmission in bed bugs. Finally, allergenic proteins excreted by anthropophilic arthropods (for example, cockroaches and house dust mites) tend to serve as aetiologic agents of human allergic disease and asthma. Bed bug infestations reach densities of thousands of individuals per home, which may generate high levels of specific antigens. The sequenced genome will provide a platform for the identification and characterization of bed bug-produced allergens that may negatively affect the health and well-being of those whose economic status, unfortunately and almost certainly, ensures that humans and bed bugs will remain closely associated for the foreseeable future.

## Methods

### Bed bug rearing and RNA/DNA extraction

The bed bug colony was originally established from bed bugs collected in Fort Dix, New Jersey in 1973 and maintained by Dr Harold Harlan (hence Ft. Dix=Harlan strain). This strain is susceptible to all insecticides and has served as a reference strain for pesticide resistance assays, transcriptomic studies and many basic physiological and behavioural studies. The colony was maintained at 27 °C, 50±5% RH and a photoperiod of 12:12 (L:D). Insects were fed in the laboratory through a parafilm-membrane feeder with defibrinated rabbit blood heated to 37 °C by a circulating water bath. Bed bugs were prepared for DNA extraction and sequencing by passing them through six generations of full-sib mating. Only a single sibling pair at each successive generation was used to parent the next generation. This inbred line, now in its 23rd generation, is available upon request (coby@ncsu.edu).

Genomic DNA was isolated from individual female adults using DNeasy Blood & Tissue Kit (Qiagen Inc, Valencia CA). Total RNA was isolated from three females and three adult males separately using the TRI reagent (Molecular Research Center Inc., Cincinnati, OH). The RNA was treated with DNase I (Ambion Inc., Austin, TX). The residual DNase I was removed using resin (Ambion Inc., Austin, TX).

### Genome sequencing and assembly

The bed bug is 1 of 30 arthropod species sequenced as a part of the pilot project for the i5K arthropod genomes project at the Baylor College of Medicine Human Genome Sequencing Center. For all of these species, an enhanced Illumina-ALLPATHS-LG sequencing and assembly strategy enabled multiple species to be approached in parallel at reduced costs. For *Cimex lectularius*, we sequenced four libraries of nominal insert sizes 180, 500, 3 and 8 kb at genome coverages of × 62.4, × 77.9, × 44.42 and × 21.21, respectively (based upon the 650.47-Mb genome size of the assembled genome). These raw sequences have been deposited in the National Center for Biotechnology Information (NCBI) SRA: SRS580017, BioSamples ID: SAMN02649412 and SAMN02434893.

To prepare the 180- and 500-bp libraries, we used a gel-cut paired-end library protocol. Briefly, 1 μg of the DNA was sheared using a Covaris S-2 system (Covaris, Inc. Woburn, MA) using the 180- or 500-bp program. Sheared DNA fragments were purified with Agencourt AMPure XP beads, end repaired, dA tailed and ligated to Illumina universal adaptors. After adapter ligation, DNA fragments were further size selected by agarose gel and PCR amplified for six to eight cycles using Illumina P1 and Index primer pair and Phusion High-Fidelity PCR Master Mix (New England Biolabs, Ipswich, MA). The final library was purified using Agencourt AMPure XP beads and quality assessed by Agilent Bioanalyzer 2100 (DNA 7500 kit, Agilent Technologies) to determine library quantity and fragment size distribution before sequencing.

Long mate-pair libraries with 3- and 8-kb insert sizes were constructed according to the manufacturer’s protocol (Mate Pair Library v2 Sample Preparation Guide art # 15001464 Rev. A PILOT RELEASE). Briefly, 5 (for 2 and 3-kb gap size library) or 10 μg (8–10-kb gap size library) of genomic DNA was sheared to desired size fragments by Hydroshear (Digilab, Marlborough, MA), then end repaired and biotinylated. Fragment sizes between 1.8 and 2.5 kb (2 kb), 3 and 3.7 kb (3 kb) or 8 and 10 kb (8 kb) were purified from 1% low-melting agarose gel and then circularized by blunt-end ligation. These size-selected circular DNA fragments were then sheared to 400 bp (Covaris S-2), purified using Dynabeads M-280 Streptavidin Magnetic Beads, end repaired, dA tailed and ligated to Illumina PE sequencing adaptors. DNA fragments with adaptor molecules on both ends were amplified for 12–15 cycles with Illumina P1 and Index primers. Amplified DNA fragments were purified with Agencourt AMPure XP beads. Quantification and size distribution of the final library was determined before sequencing as described above.

Sequencing was performed on Illumina HiSeq2000s generating 100-bp paired-end reads. Reads were assembled using ALLPATHS-LG (v35218, http://www.broadinstitute.org/software/allpaths-lg/blog/) and further scaffolded and gap-filled using in-house tools Atlas-Link (v.1.0) and Atlas gap-fill (v.2.2) (https://www.hgsc.bcm.edu/software/). This yielded an assembly of size 650.47 Mb with contig N50 of 23.5 kb and scaffold n50 of 7.17 Mb. The assembly has been deposited in the NCBI: BioProject PRJNA167477.

### Automated gene annotation using a Maker 2.0 pipeline adapted for arthropods

The bed bug is 1 of 30 i5K pilot genome assemblies that were subjected to automatic gene annotation using a Maker 2.0 (http://www.yandell-lab.org/software/maker.html) annotation pipeline tuned specifically for arthropods. The pipeline is designed to be systematic, providing a single consistent procedure for the species in the pilot study, scalable to handle 100s of genome assemblies, evidence guided using both protein and RNA-seq evidence to guide gene models and targeted to utilize extant information on arthropod gene sets. The core of the pipeline was a Maker 2 instance, modified slightly to enable efficient running on our computational resources. The genome assembly was first subjected to *de novo* repeat prediction and CEGMA analysis (http://korflab.ucdavis.edu/datasets/cegma/) to generate gene models for initial training of the *ab initio* gene predictors (Supplementary Data 33). Three rounds of training of the Augustus (http://bioinf.uni-greifswald.de/augustus/) and SNAP (http://korflab.ucdavis.edu/software.html) gene predictors within Maker were used to bootstrap to a high-quality training set. Input protein data included 1 million peptides from a non-redundant (nr) reduction (90% identity) of Uniprot Ecdysozoa (1.25 million peptides) supplemented with proteomes from 18 additional species (*Strigamia maritima, Tetranychus urticae, Caenorhabditis elegans, Loa loa, Trichoplax adhaerens, Amphimedon queenslandica, Strongylocentrotus purpuratus, Nematostella vectensis, Branchiostoma floridae, Ciona intestinalis, Ciona savignyi, Homo sapiens, Mus musculus, Capitella teleta, Helobdella robusta, Crassostrea gigas, Lottia gigantea* and *Schistosoma mansoni*) leading to a final nr peptide evidence set of 1.03 million peptides. RNA-seq from *C. lectularius* adult males and females was used judiciously to identify exon–intron boundaries but with a heuristic script to identify and split erroneously joined gene models. We used CEGMA models for QC purposes: for *C. lectularius*, of 1,977 CEGMA single-copy orthologue gene models, 1,928 were found in the assembly, and 1,892 in the final predicted gene set. Finally, the pipeline uses a nine-way homology prediction with human, *Drosophila* and *C. elegans*, and InterPro Scan5 to allocate gene names. The automated gene set is available from the BCM-HGSC website (https://www.hgsc.bcm.edu/arthropods/bed-bug-genome-project) and at the National Agricultural Library (https://i5k.nal.usda.gov).

### Community curation of the bed bug genome

Thirty-two groups were recruited through the i5k pilot project to manually curate the MAKER-predicted gene set Clec_v0.5.3. These curators selected genes or gene families based on their own research interests. Manual curation occurred via the Web Apollo software, a web-based graphical user interface for gene model curation that allows curators to create and view changes to gene models in real time. A *C. lectularius* Web Apollo (http://genomearchitect.org/) instance was made available (https://apollo.nal.usda.gov/cimlec/jbrowse/) to display evidence included in the generation of the Clec_v0.5.3 gene predictions. This Web Apollo instance also incorporates aligned RNA-seq and transcriptome data sets provided to the scientific community by Zach Adelman (Virginia Tech) that were not included in the MAKER analysis. Curators were provided a webinar-based training session on the Web Apollo software (courtesy of Monica Muñoz-Torres, Lawrence Berkeley National Laboratory) and were asked to adhere to a set of curation rules (https://i5k.nal.usda.gov/content/rules-web-apollo-annotation-i5k-pilot-project). After the curation period, the manually curated models were exported in gff3 format and quality checked for formatting and curation errors, and then integrated with the MAKER-predicted gene models to generate a non-redundant official gene set OGSv1.1. A subsequent quality-control check using two separate gff3-checking pipelines (https://github.com/hotdogee/gff3-py/releases/tag/0.3.0; https://github.com/chienyuehlee/gff-cmp-cat) and custom scripts resulted in an updated OGSv1.2.

### Orthology/phylogeny analyses

The OrthoDB (http://orthodb.org/) resource was used to find shared orthologues among *C. lectularius* (v1.0) and seven other arthropods: *Daphnia pulex*, *Pediculus humanus humanus* (=*Pediculus humanus corporis*), *Acyrthosiphon pisum*, *Apis mellifera*, *Tribolium castaneum*, *Danaus plexippus* and *Drosophila melanogaster.* Custom Perl scripts were used to find the number of genes in each category (Fig. 1, single copy, present in all species and so on). For the phylogenetic analysis, only the single-copy orthologues were used to build a concatenated phylogenetic tree using RAxML (http://sco.h-its.org/exelixis/software.html). Briefly, a multiple sequence alignment was performed using MUSCLE (http://www.drive5.com/muscle/) for each orthologous cluster, separately. Then, the resulting alignments were trimmed using trimAI (http://trimal.cgenomics.org/) and these alignments were concatenated using the ‘seqret’ program from the EMBOSS suite (http://emboss.sourceforge.net/). This concatenated alignment was used to build the phylogeny using RAxML 7.6.6 with 100 bootstraps.

### BUSCO-based quality assessment

The completeness of genome assemblies can be measured by searching for the presence of conserved genes. Absence of such genes means that the assembly is incomplete to a greater or lesser degree depending on the fraction of missing genes. Moreover, if these conserved genes are also single copy, the assembly can also be tested for unexpected duplications, which is a sign of erroneous haplotype assembly. To this end, we used the BUSCO (http://busco.ezlab.org/)^18^, to measure the completeness of the bed bug genome as well as its set of predicted genes (Supplementary Data 3). We used the Arthropoda gene set, which consists of 2,675 single-copy genes that are present in at least 90% of Arthropoda.

### Lateral gene transfer identification

Putative LGT events in the assembled bed bug genome were computationally identified using two different python-based computational pipelines. The bed bug assembly was first analysed using the homology-based pipeline described in Wheeler *et al.*^43^ Because this pipeline outputs data on the best putative LGT candidate on each scaffold (best on e-value), along with the number and range of putative LGTs on the scaffold, it was most helpful for identifying scaffolds that appear to be of bacterial, not bed bug, origin (Supplementary Data 21). In addition, if the best putative LGT on each scaffold had a higher bacterial score than animal score, it was manually annotated using Blastn and Blastx analysis to the NCBI nr/nt database. In addition, 12 scaffolds with a high number of potential LGTS were manually searched for additional LGT candidates using Blastn similarity (e-value 1e−5 cutoff) on the NCBI nr/nt database.

Due to the vast number of putative LGT candidates, a second python script was used to break long scaffolds into 1,000-bp intervals and search each of them against the bacterial database. Any positive hits of the 1,000-bp regions were then searched against the animal database. The bacterial database contained ∼1,000 bacterial species and was masked for low-complexity regions using the NCBI Dustmasker function. The animal database contained transcripts from a representative from each of the following animal genera: *Anopheles, Apis, Drosophila, Xenopus, Tribolium, Nasonia, Daphnia, Strongylocentrotus, Mus, Homo sapiens, Aplysia, Caenorhabditis, Hydra, Monosiga* and *Acanthamoeba*. The significance e-value cutoff used was 1e−5 for both the animal and bacterial hits. Regions of bacterial similarity that fell from the end of one 1,000-bp interval to the adjacent interval were joined if they were <50-bp apart. Putative LGT regions that were ≥100 bp were used in the final analysis. As a further computational confirmation, junctions between candidate LGTs and flanking eukaryotic sequences were confirmed using mate-pair data from the different insert size libraries.

*Note added in proof:* Since this manuscript was submitted the genome of the closely related hematophagous hemipteran, *Rhodnius prolixus*, was published (Mesquita *et al*. Genome of *Rhodnius prolixus*, an insect vector of Chagas disease, reveals unique adaptations to hematophagy and parasite infection. *Proc. Natl Acad. Sci.* USA (early edition) doi/10.1073/pnas.1506226112) and has not been included in analyses associated with this study.

## Additional information

**Accession codes:** Data for the *Cimex lectularius* genome has been deposited in the GenBank/EMBL/DDBJ Bioproject database under the accession code PRJNA167477. Raw genomic sequence data is deposited in the GenBank/EMBL/DDBJ sequence read archive under the accession codes SRX498126, SRX498127, SRX498128, and SRX498129. The genome assembly has been deposited in GenBank under the accession code GCA_000648675.1. RNA-seq datasets used in gene prediction have been deposited to the in GenBank/EMBL/DDBJ sequence read archive under the accession codes SRX906994 and SRX907005.

**How to cite this article:** Benoit, J. B. *et al.* Unique features of a global human ectoparasite identified through sequencing of the bed bug genome. *Nat. Commun.* 7:10165 doi: 10.1038/ncomms10165 (2016).

## Supplementary Material

Supplementary Information Supplementary Figures 1-42, Supplementary Notes 1-22 and Supplementary References

Supplementary Data 1 Sequencing, assembly, annotation statistics and accession numbers

Supplementary Data 2 A summary of all curated genes, pseudogenes, mRNAs, and pseudogenic transcripts.

Supplementary Data 3 Antioxidant gene families and their function

Supplementary Data 4 Details of antioxidant genes identified from C. lectularius genome.

Supplementary Data 5 Primary and secondary antioxidants genes identified in the bed bug genome. Accession numbers of homologs from *Drosophila melanogaster*, *Rhondnius prolixus, Anopheles gambiae, Pediculus humanus* and *Tribolium casteneum* are shown.

Supplementary Data 6 Details of ClOr family genes and proteins.

Supplementary Data 7 Details of ClIr family genes and proteins.

Supplementary Data 8 Details of ClGr family genes and proteins.

Supplementary Data 9 Clock genes identified in the bed bug genome.

Supplementary Data 10 Number of putative cuticle protein genes per family in the bed bug genome.

Supplementary Data 11 Cuticle protein-encoding gene glusters in the bed bug genome.

Supplementary Data 12 Summary of the repertoire of digestive genes in Cimex lectularius.

Supplementary Data 13 Number of loci within the genomes of arthropod species encoding the five classes of histones.

Supplementary Data 14 Cimex autophagy associated genes.

Supplementary Data 15 Cimex heat shock associated genes.

Supplementary Data 16 Summary information for all annotated Hox genes.

Supplementary Data 17 putative prepropeptides and peptide receptors predicted from the bed bug genome.

Supplementary Data 18 putative aminergic receptors predicted from the bed bug genome.

Supplementary Data 19 B vitamin metabolic genes.

Supplementary Data 20 List of UDP-glycosyltransferase genes in Cimex lectularius genome

Supplementary Data 21 Bacterial Scaffolds in the C. lectularius assembly.

Supplementary Data 22 P450 genes identified in the genome.

Supplementary Data 23 ABC Transporters identified in the bed bug genome.

Supplementary Data 24 Carboxyl esterase identified in the bed bug genome.

Supplementary Data 25 Glutathione-s- transferases identified in the bed bug genome.

Supplementary Data 26 Nuclear receptors identified in the bed bug genome.

Supplementary Data 27 Genes related to development and reproduction identified in the bed bug genome.

Supplementary Data 28 BUSCO quality data from the bed bug genome.

Supplementary Data 29 Immune genes from the bed bug genome.

Supplementary Data 30 Sex determination genes.

Supplementary Data 31 Transcription factors with putative DNA binding motifs from the bed bug genome.

Supplementary Data 32 Transcription factors without putative DNA binding motifs from the bed bug genomes.

Supplementary Data 33 Cegma Statistics based on 248 CEGs suggest a high level of completeness for the bedbug genome.
