# Supplementary material for: Unique features of a global human ectoparasite identified through sequencing of the bed bug genome
Source: Nat Commun. 2016 Feb 2;7:10165. doi: 10.1038/ncomms10165 (PMC4740739; doi:10.1038/ncomms10165)
Supplement: Supplementary Information — Supplementary Figures 1-42, Supplementary Notes 1-22 and Supplementary References [file ncomms10165-s1.pdf]

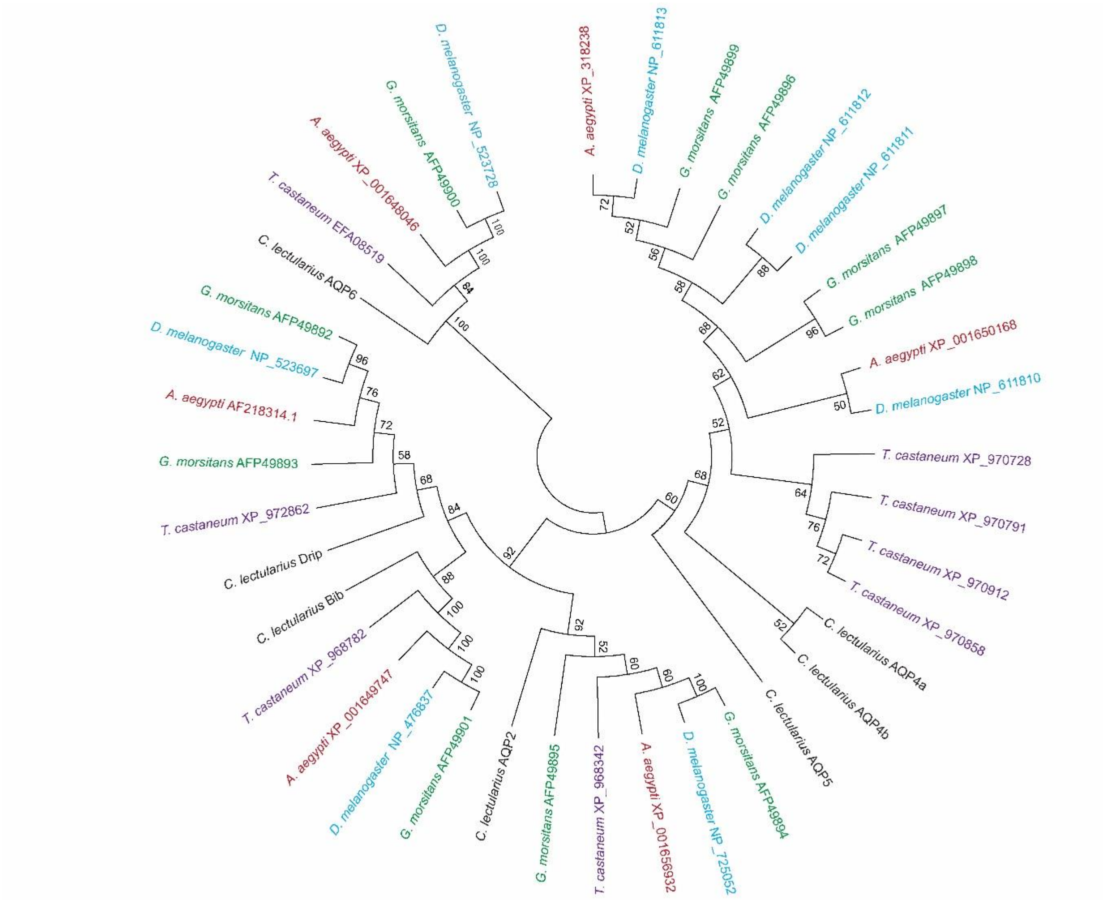

Supplementary Figure 1. Comparison of predicted bed bug aquaporins to aquaporins from other insects. Neighbor-joining tree was produced using MEGA6 using Dayhoff Model and pairwise matching; branch values indicate support following 1000 bootstraps; values below 50% are omitted.

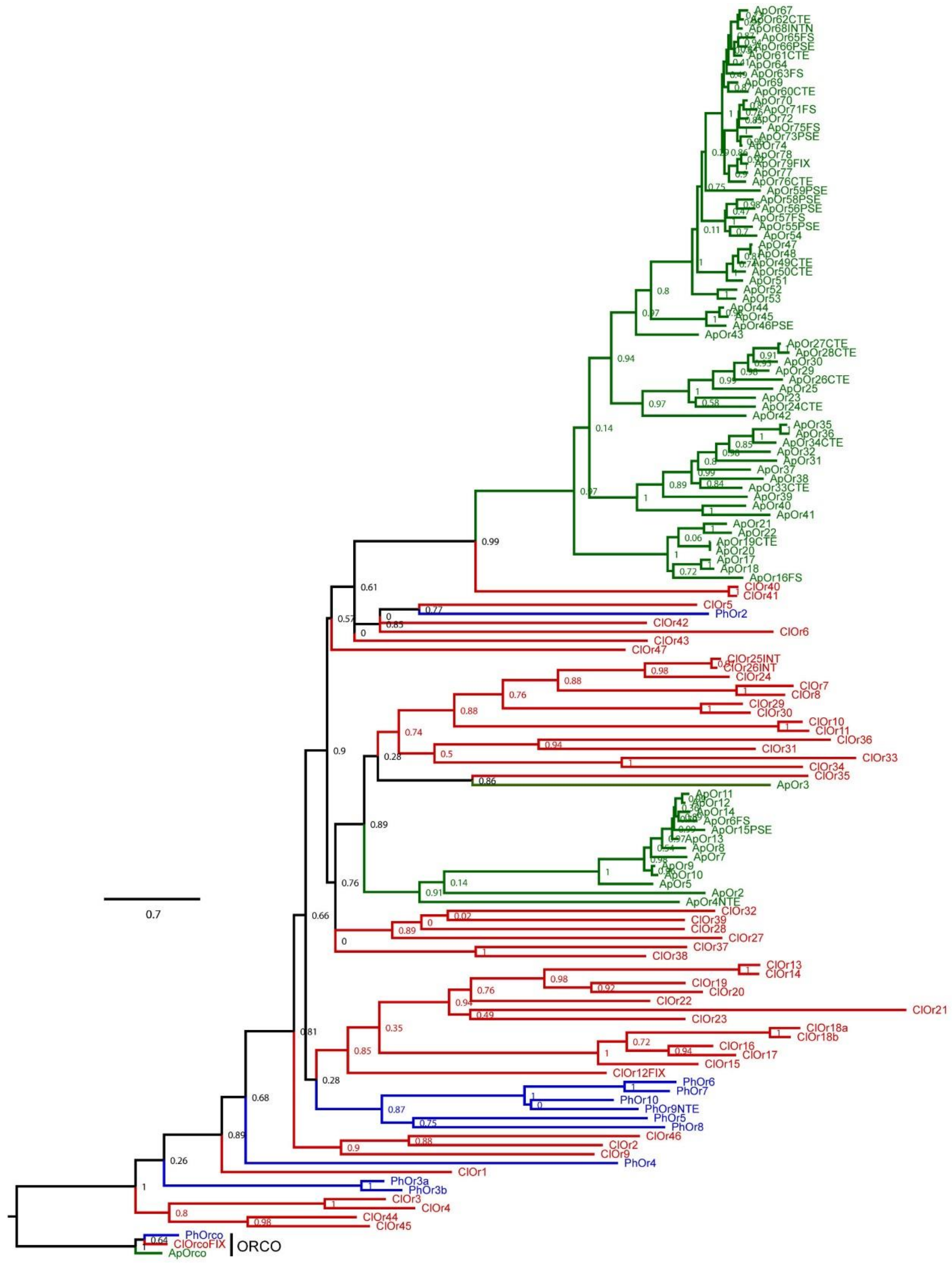

Supplementary Figure 2. Phylogenetic tree of the *Cimex*, *Acyrthosiphon*, and *Pediculus* ORs. The ORCO orthologs from each species were declared as the outgroup to root the tree. *Cimex*, *Acyrthosiphon*, and *Pediculus* gene/protein names are highlighted

in red, green, and blue, respectively, as are the branches leading to them to emphasize gene lineages. Suffixes after the gene/protein names are: PSE – pseudogene; NTE – N-terminus missing; CTE – C-terminus missing; INT – internal sequence missing; FIX – sequence fixed with raw reads; JOI – gene model joined across scaffolds; multiple suffixes are abbreviated to single letters.

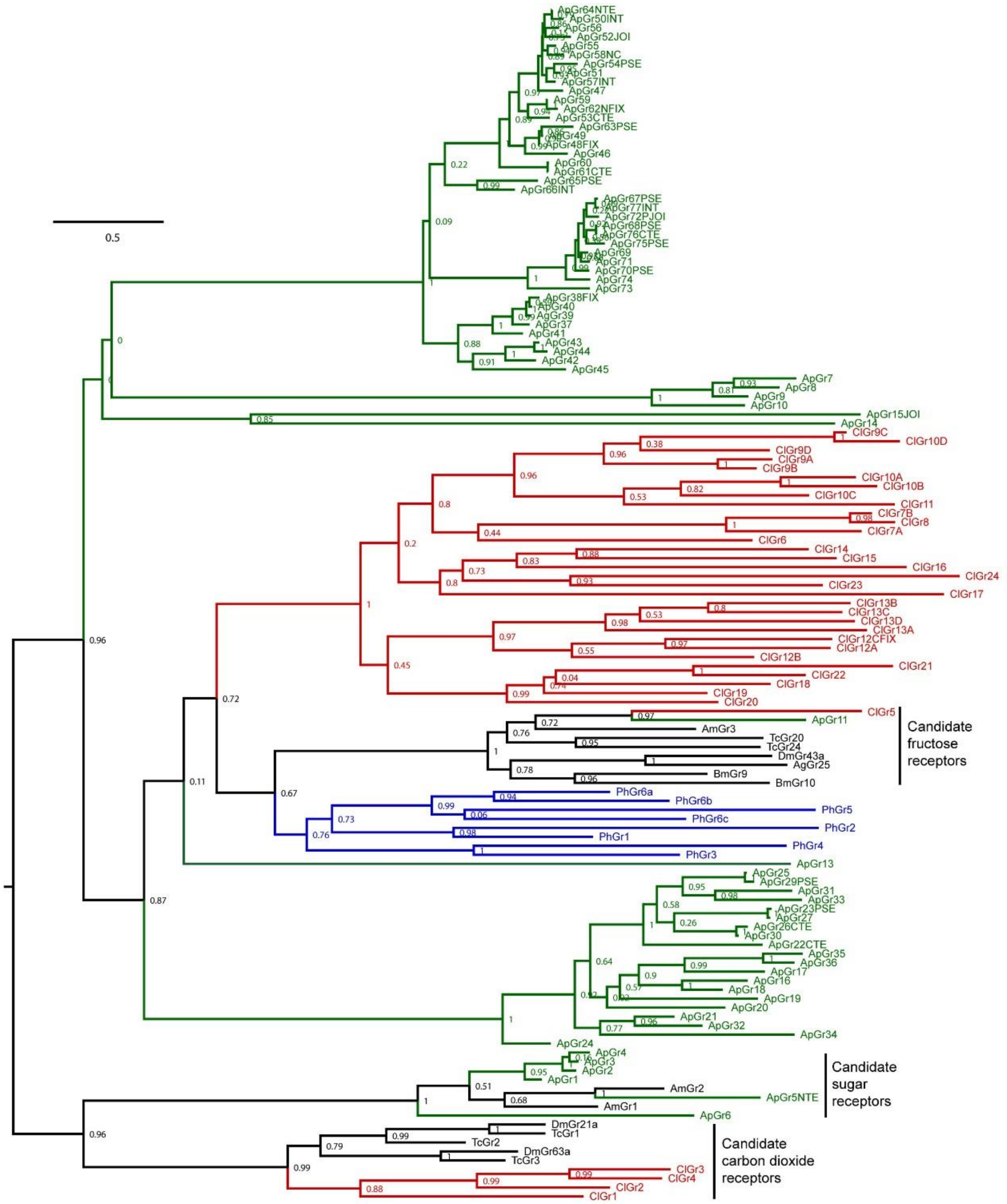

Supplementary Figure 3. Phylogenetic tree of the *Cimex*, *Acyrthosiphon*, and *Pediculus* GRs, and representative GRs from other insects. See Figure S2 legend for details. The tree was rooted with the candidate carbon dioxide and sugar receptors, both sets of conserved GRs that appear to be a basal lineage within the insect Grs. The other insect GRs are in black.

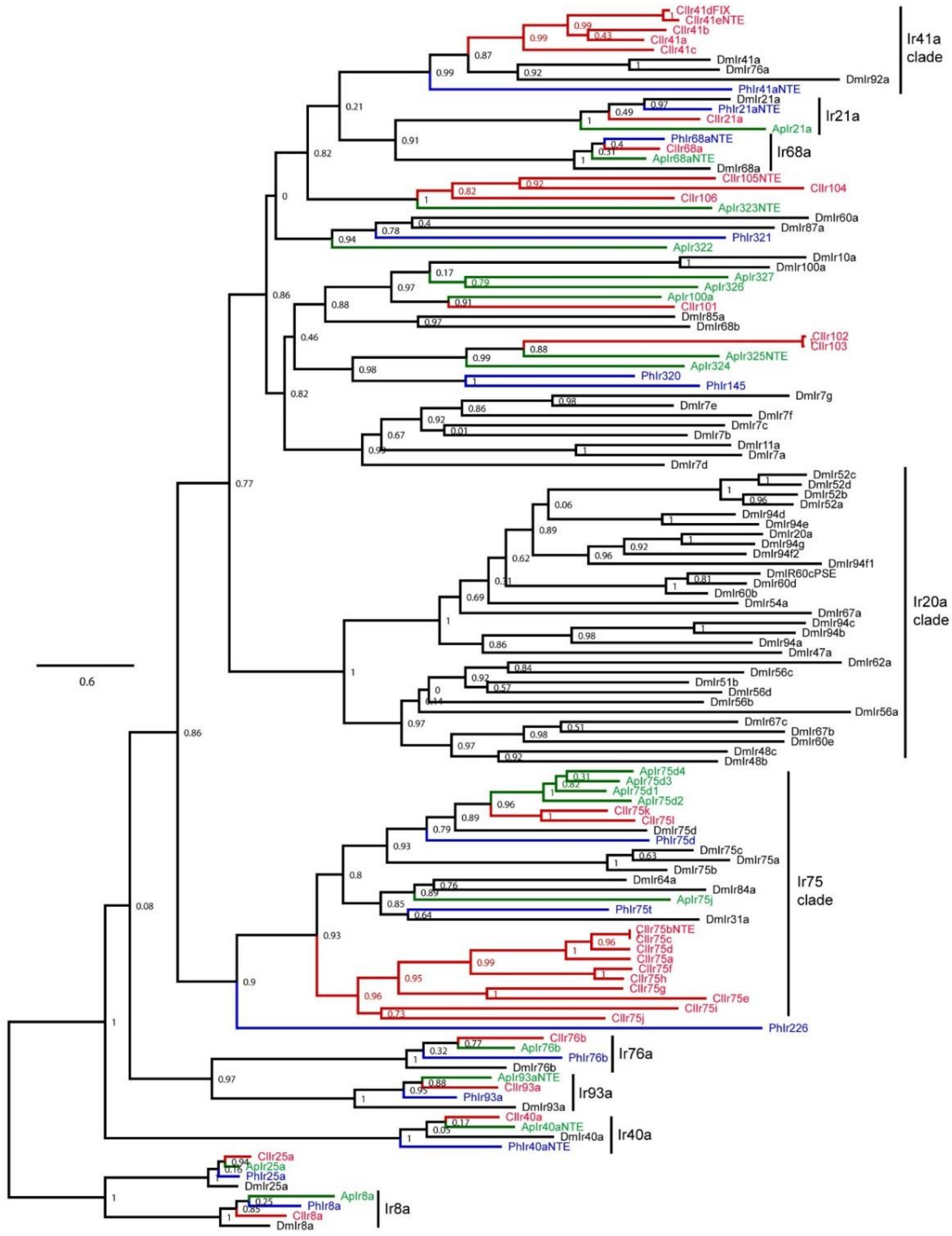

Supplementary Figure 4. Phylogenetic tree of the *Cimex*, *Acyrthosiphon*, *Pediculus*, and *Drosophila* IRs. See Supplementary Figure 2 legend for details. The tree was rooted with the Ir25a and 8a proteins that are convincing outgroups as they cluster with the ionotropic glutamate receptors in larger trees[1,2]. The *Drosophila melanogaster* IRs are in black.

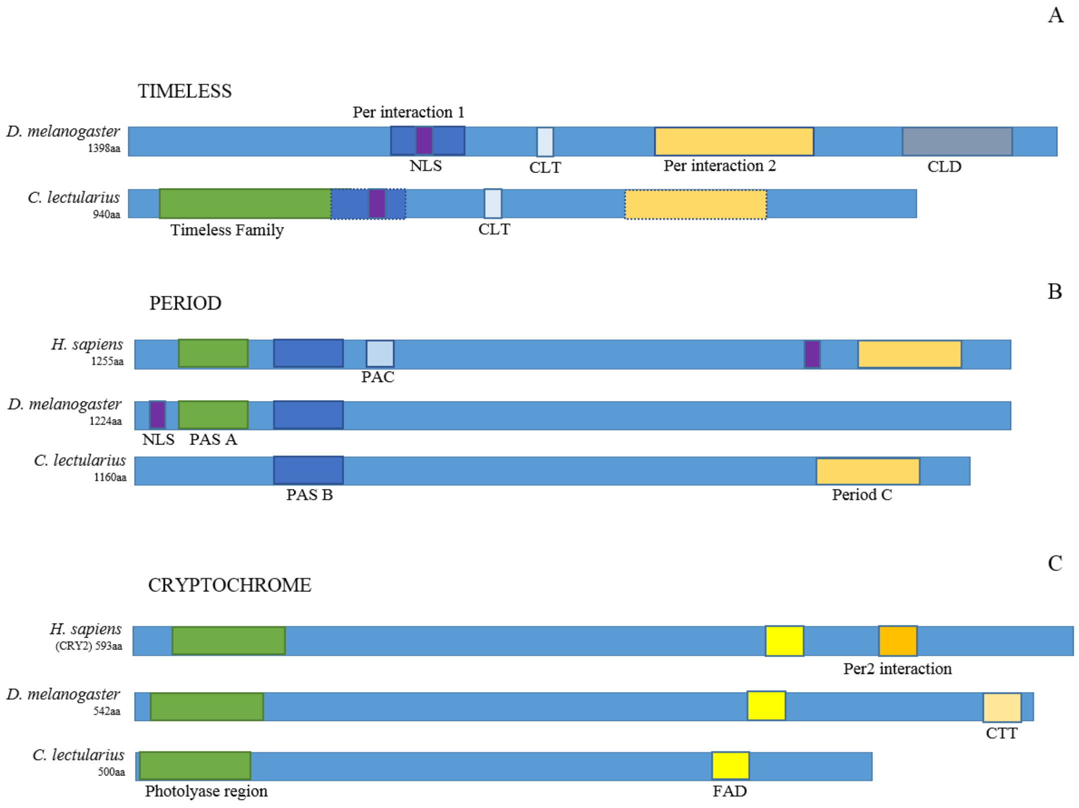

Supplementary Figure 5. Schematic representation of TIM (A), PER (B) and CRY (C) structures in *H. sapiens*, *D. melanogaster*, and *C. lectularius*. CTT: C-terminal tail; CLT: CTT like domain; NLS: Nuclear localization sequence; CLD: cytoplasmic localization domain; PAS: Per- Arnt- Sim domains; PAC: C-terminal to PAS; FAD: FAD binding domain.

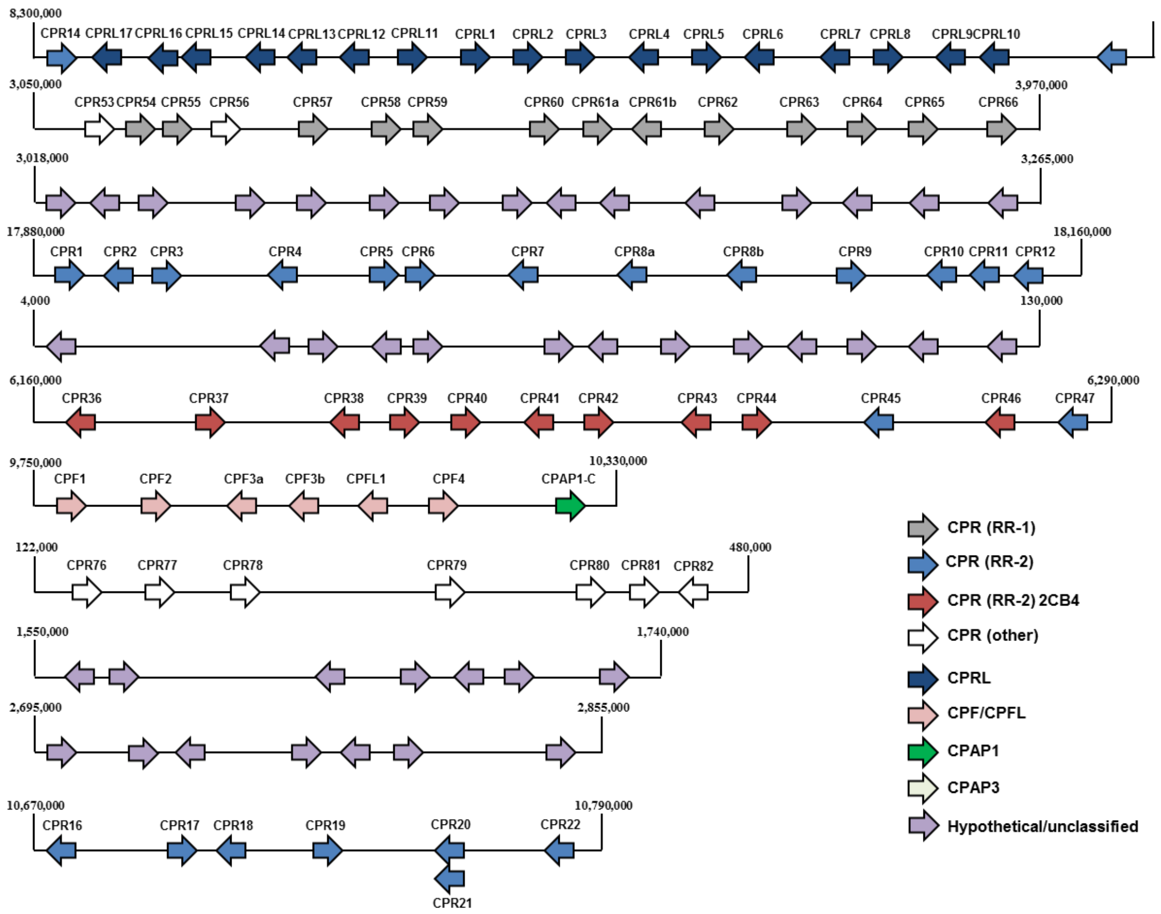

Supplementary Figure 6a. Bed bug cuticle gene clusters. Arrows indicate the orientation (but not gene structure) of each putative bed bug cuticle protein encoding gene. Scaffold (Scaf#) number as well as genomic coordinates are indicated (not to scale).

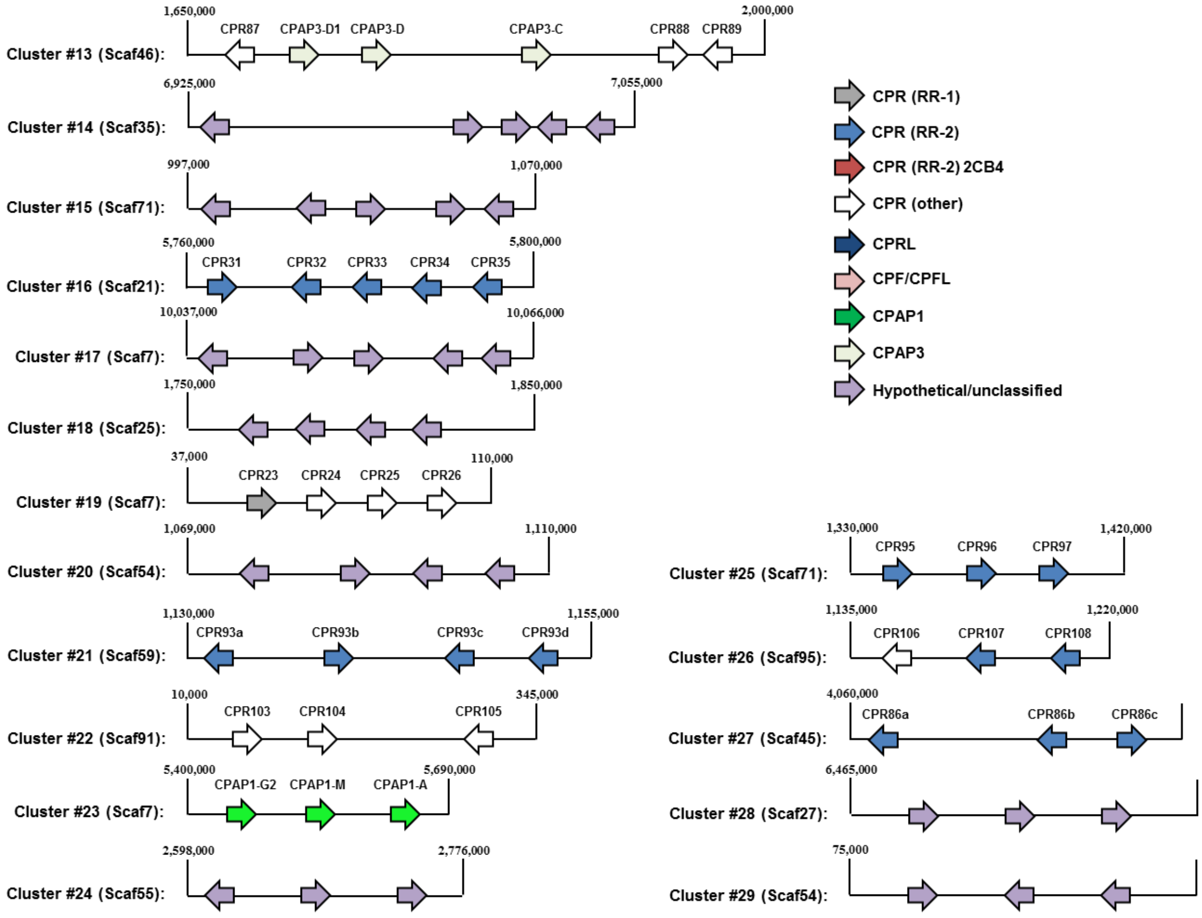

Supplementary Figure 6b. Bed bug cuticle gene clusters. Arrows indicate the orientation (but not gene structure) of each putative bed bug cuticle protein encoding gene. Scaffold (Scaf#) number as well as genomic coordinates are indicated (not to scale).

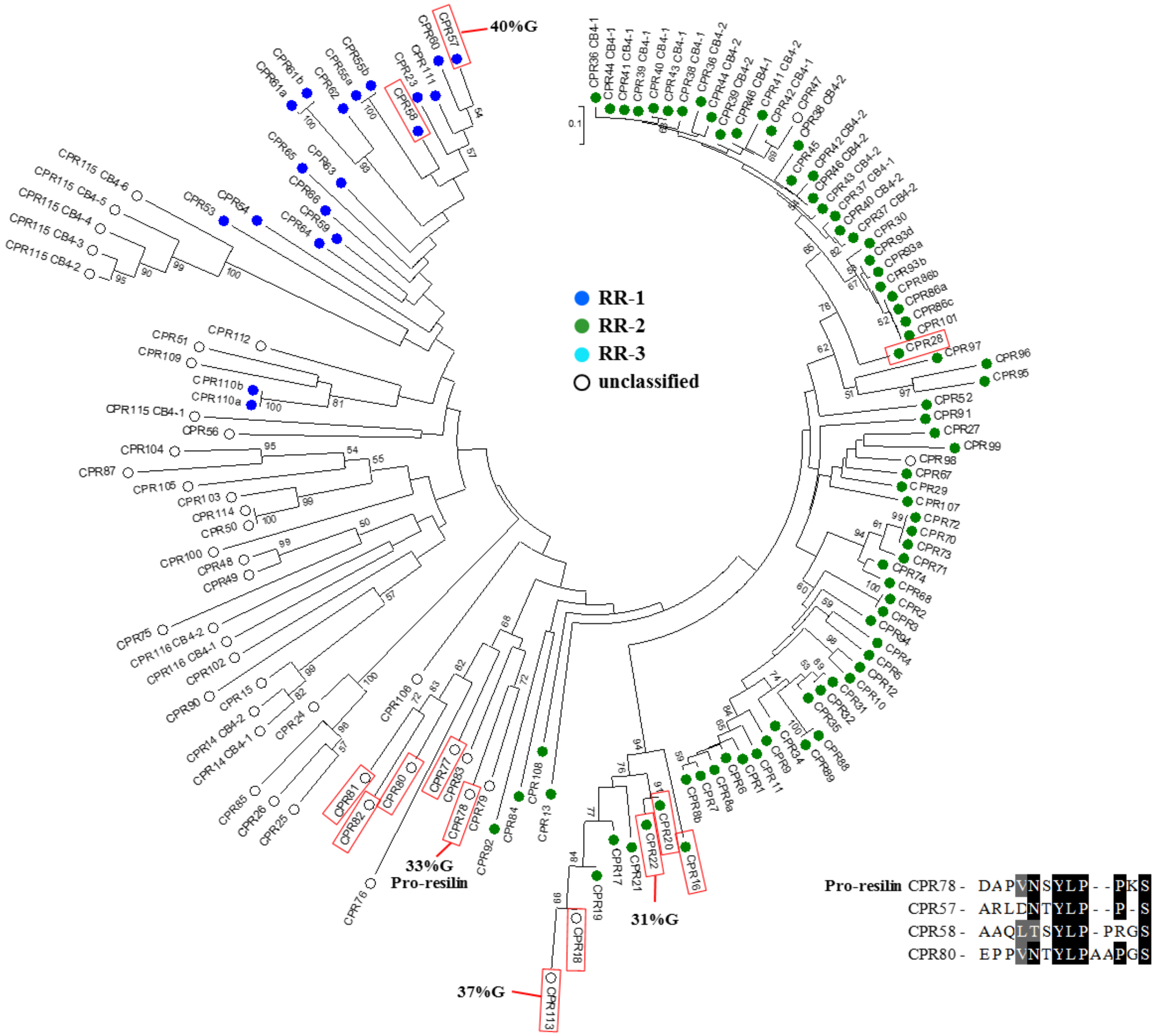

Supplementary Figure 7. Bed bug CB4 domains from CPR-type cuticle proteins. Neighbor-joining tree of CB4 (Pfam000379) chitin-binding domains extracted from each predicted protein sequence. Proteins classified as RR-1 or RR-2 by cuticleDB[3,4] are indicated. Red boxes indicate full protein sequences that contain >20% glycine; genes with >30% glycine content are indicated directly. Of these genes, four contained an N-terminal pro-resilin domain (indicated at bottom right). Tree was produced in MEGA6[5] after the initial ClustalW alignment was adjusted manually to account for known critical

residues in the CB4 domain. Branch values indicate support following 2000 bootstraps; values below 50% are omitted.

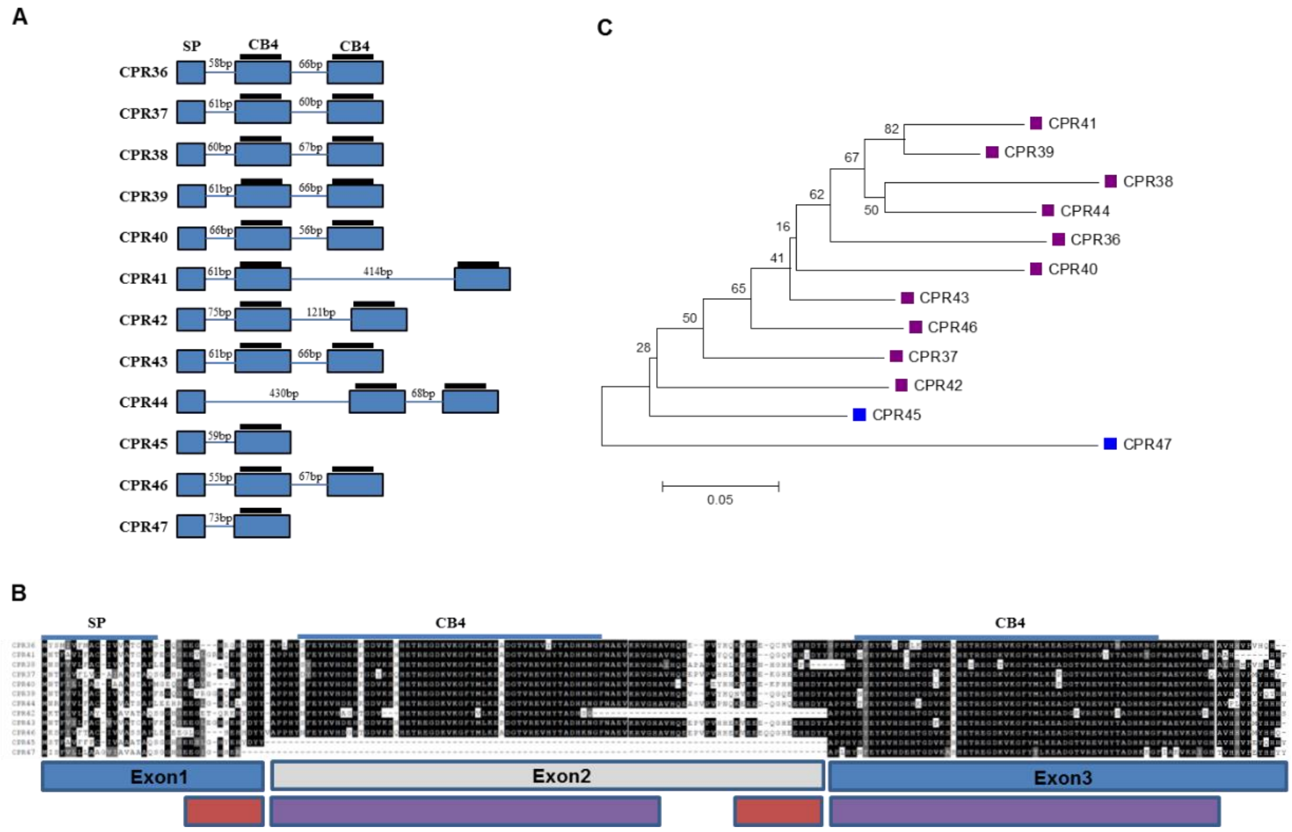

Supplementary Figure 8. A novel duplication event results in a new type of CPR containing two CB4 domains. (**A**) Gene structure of each of the 12 members of cluster #4. Boxes represent exons; lines represent introns. Black bars represent the CB4 domain. (**B**) Whole protein alignment of the 12 CPRs found in cluster #4. Residues identical (black) or similar (grey) in 70% of the sequences are highlighted. Red block indicates residues that are duplicated at the end of exons one and two; purple block indicates residues duplicated in exons two and three. (**C**) Neighbor-joining tree produced from the whole protein alignment of cluster 4 CPRs. Branch values indicate support following 2000 bootstraps; values below 50% are omitted.

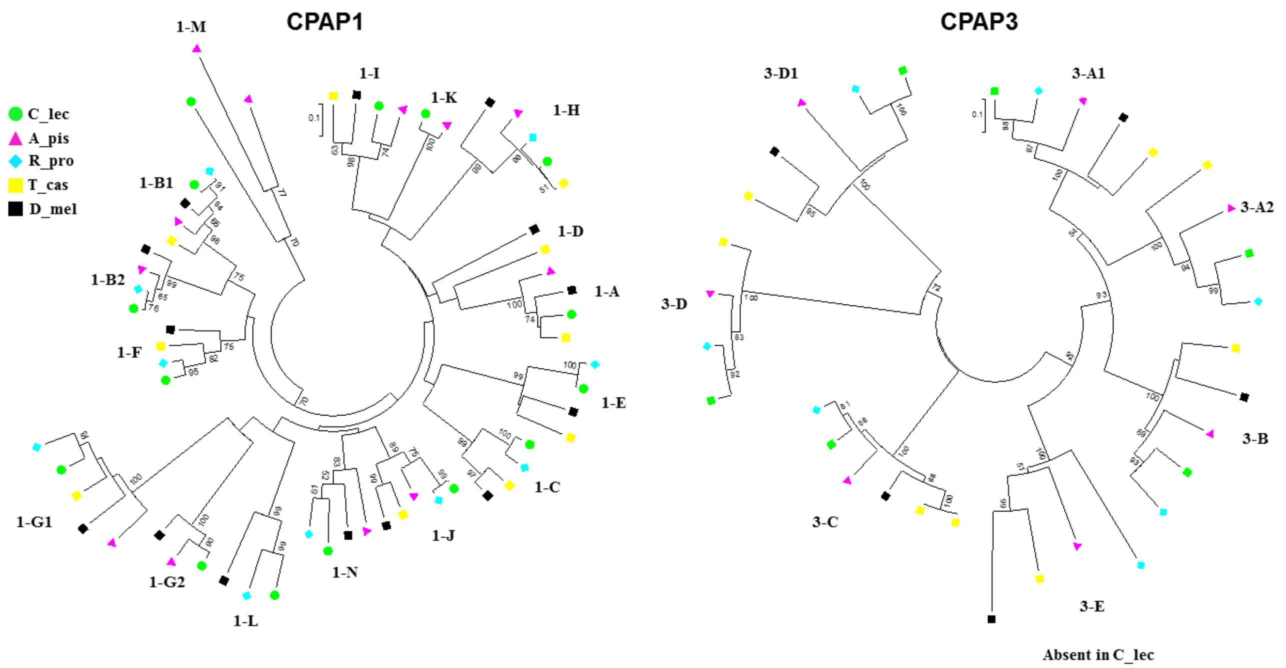

Supplementary Figure 9. Comparison of predicted bed bug CPAP proteins with other insects. Bed bug proteins (green) in the CPAP1 or CPAP3 families were compared with *R. prolixus* (blue), *A. pisum* (pink), *T. castaneum* (yellow) and *D. melanogaster* (black). Neighbor-joining tree was produced using MEGA6; branch values indicate support following 2000 bootstraps; values below 50% are omitted.

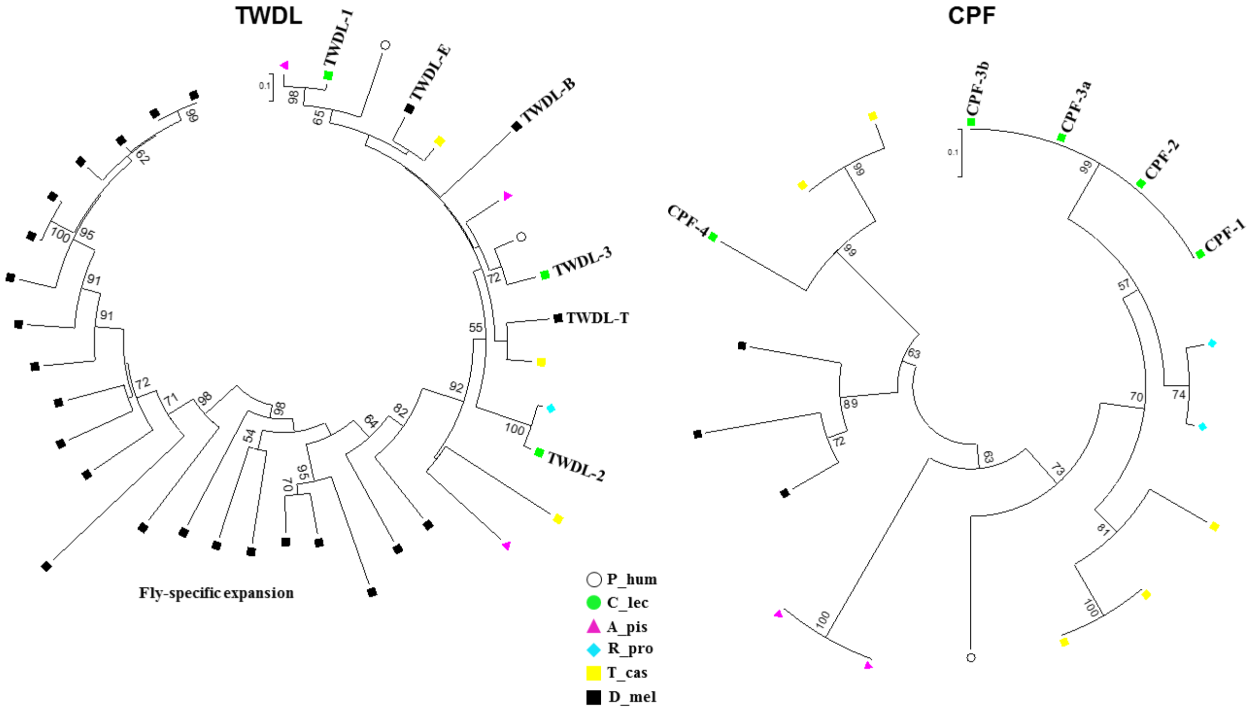

Supplementary Figure 10. Comparison of predicted bed bug Tweedle (Twdl) and CPF proteins with other insects. Bed bug proteins (green) in the CPAP1 or CPAP3 families were compared with *R. prolixus* (blue), *A. pisum* (pink), *T. castaneum* (yellow) and *D. melanogaster* (black). Neighbor-joining tree was produced using MEGA6; branch values indicate support following 2000 bootstraps; values below 50% are omitted.

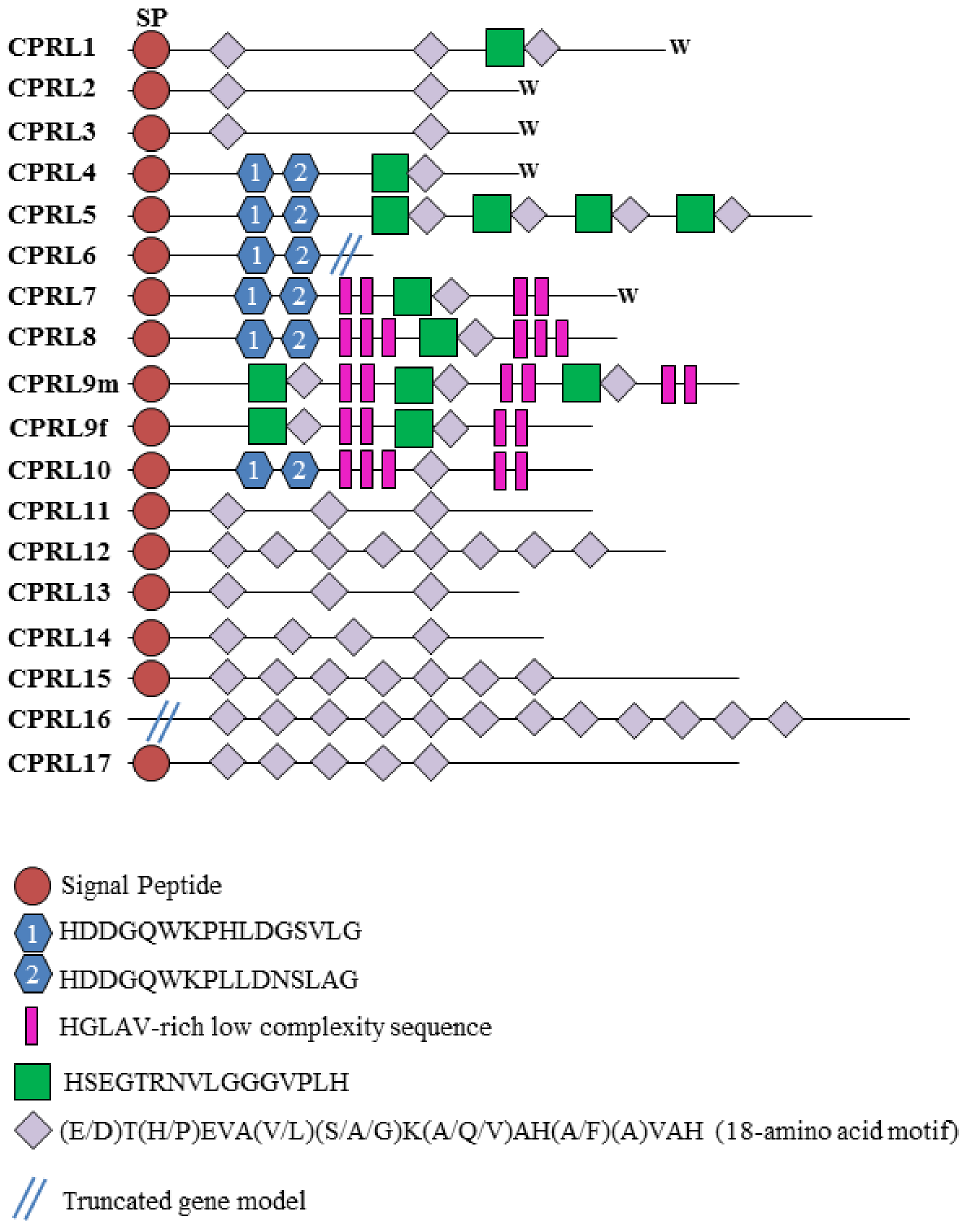

Supplementary Figure 11. Domain architecture of the bed bug CPRL family. Proteins ending in Trp (W) are indicated. Diagrams are not to scale.

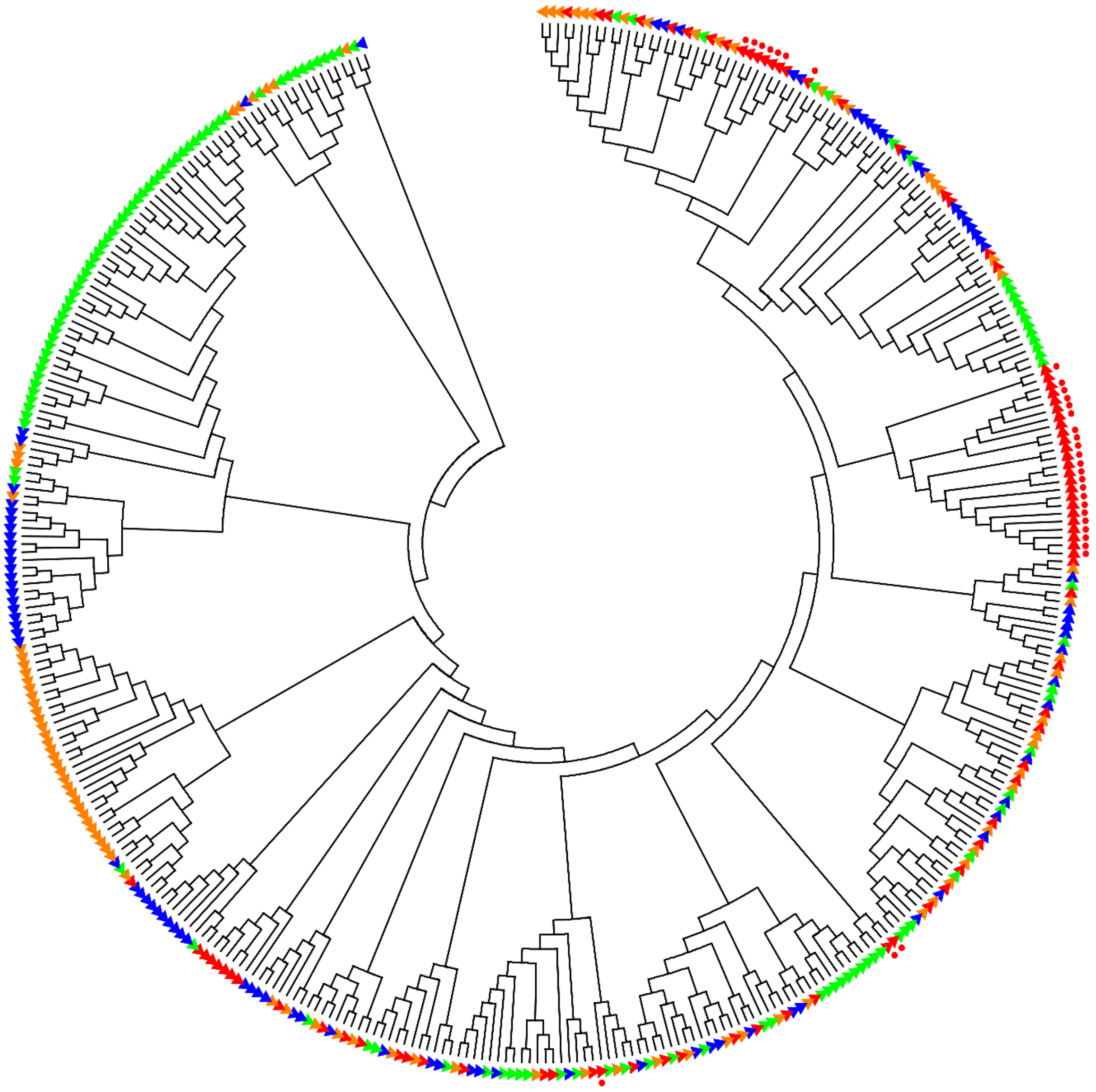

Supplementary Figure 12. The Neighbor-Joining inferred phylogeny of hemipteran putative serine proteases. Proteins derived from four insect species are respectively represented by triangles in four different colors: red (*Cimex lectularius*), green (*Acyrthosiphon pisum*), blue (*Nilaparvata lugens*), and orange (*Rhodnius prolixus*). Red dots indicate *C. lectularius* serine protease genes consisting of single exons.

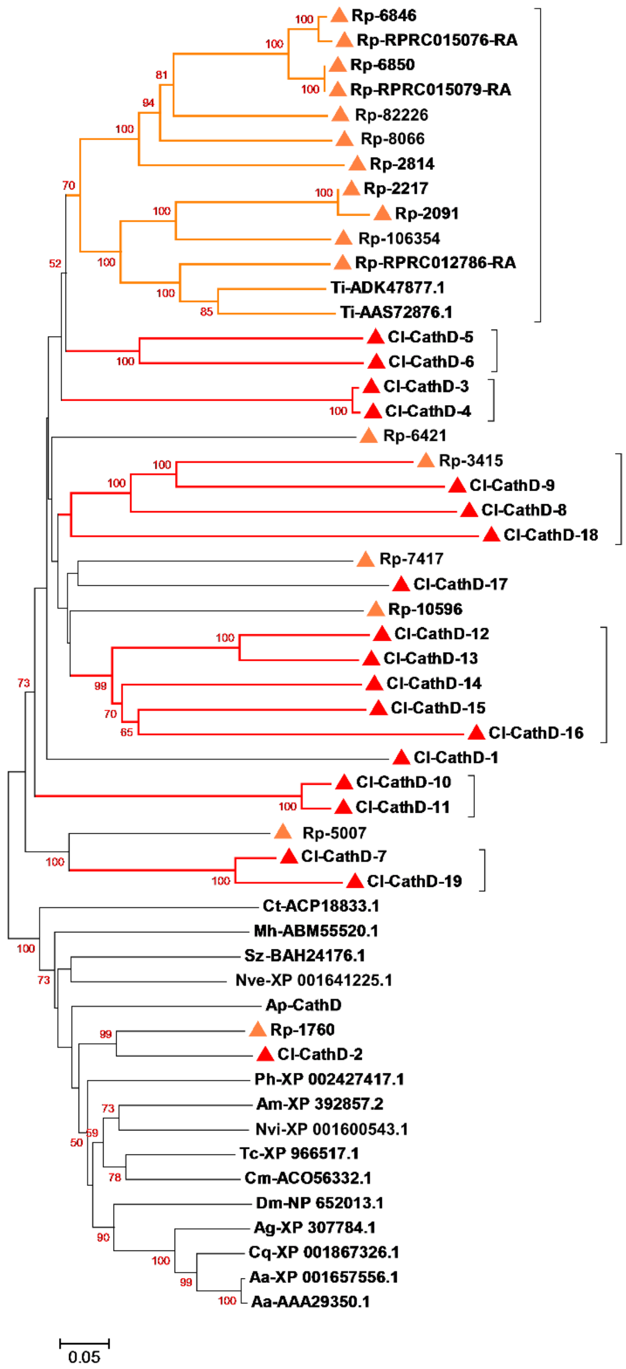

Supplementary Figure 13. The Neighbor-Joining inferred phylogeny of insect putative cathepsin D proteins. Sequences derived from *Cimex lectularius* (Cl) are denoted with red triangles and those derived from *Rhodnius prolixus* (Rp) are denoted with orange triangles. Other insect cathepsin D proteins represent those of *Triatoma infestans* (Ti), *Acyrthosiphon pisum* (Ap), *Anopheles gambiae* (Ag), *Drosophila melanogaster* (Dm), *Pediculus humanus corporis* (Ph), *Apis mellifera* (Am), *Nasonia vitripennis* (Nvi), *Tribolium castaneum* (Tc), *Callosobruchus maculatus* (Cm), *Sitophilus zeamais* (Sz), *Chrysomela tremula* (Ct), *Maconellicoccus hirsutus* (Mh), *Nematostella vectensis* (Nve), *Culex quinquefasciatus* (Cq) and *Aedes aegypti* (Aa).

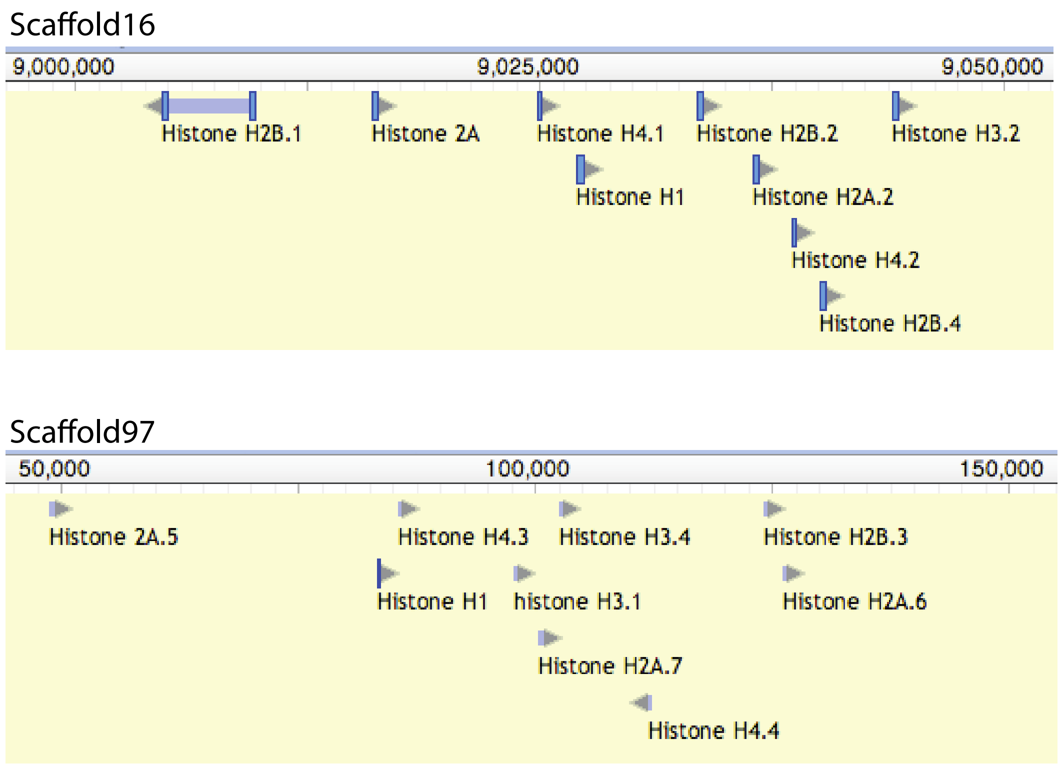

Supplementary Figure 14. Screenshots showing the histone loci complexes on scaffolds 16 and 97 of the *Cimex* genome.

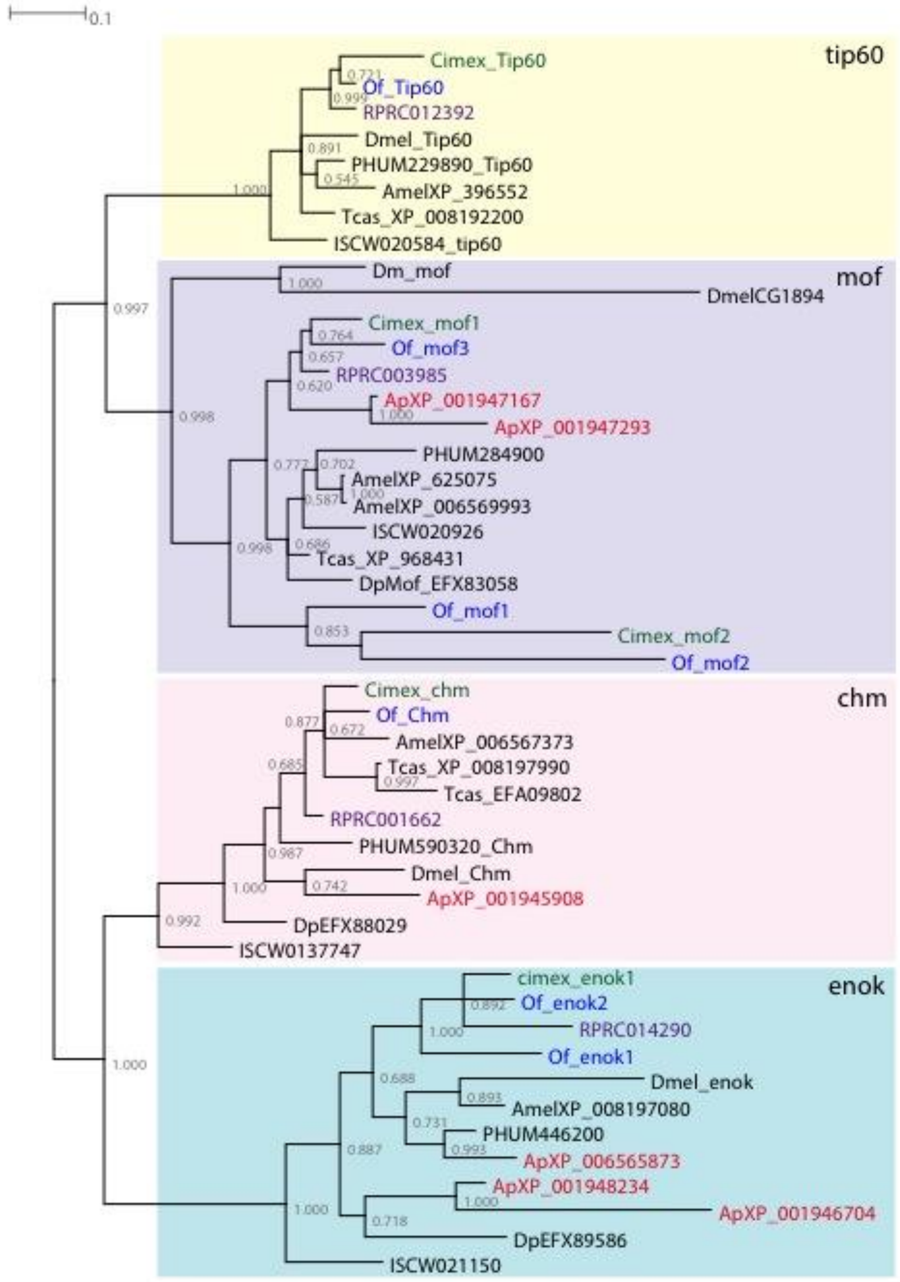

Supplementary Figure 15. Bayesian phylogeny of MYST histone acetyltransferases (posterior probabilities are shown at nodes). Protein sequences were subjected to Bayesian phylogenetic analysis using MrBayes[6]. Phylogenetic relationships were reconstructed using the WAG amino acid substitution model[6], which was found to be the most appropriate after preliminary investigations using mixed models. The first 25% of

trees were discarded as burn-in and the remaining trees summarized and visualized using Dendroscope[7].

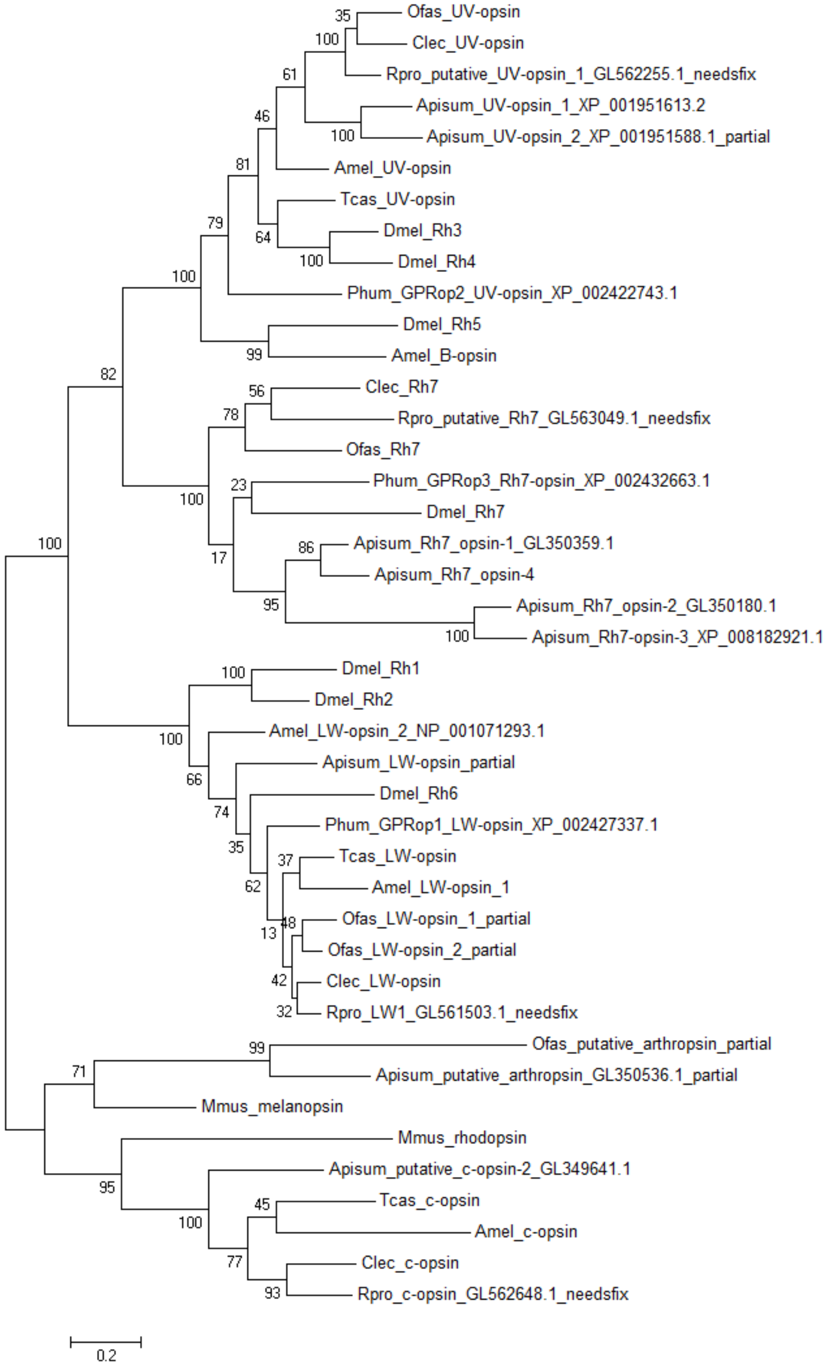

Supplementary Figure 16. Nonparametric bootstrap maximum likelihood tree of opsin genes from hemipteran, holometabolous insect species, and mouse. Species abbreviations: Amel = *Apis mellifera*, Apisum = *Acyrthosiphon pisum*, Clec = *Cimex lectularius*, Dmel = *Drosophila melanogaster*, Mmus = *Mus musculus*, Ofas = *Oncopeltus*

fasciatus, Phum = *Pediculus humanus*, Rpro = *Rhodnius prolixus*, Tcas = *Tribolium castaneum*.

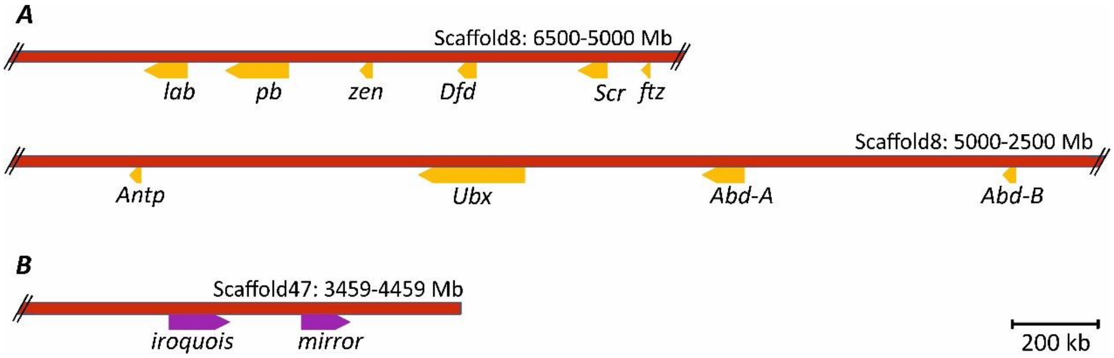

Supplementary Figure 17. Schematic representation of the Hox (A) and Iro-C clusters (B), shown to scale for gene loci and transcriptional orientation.

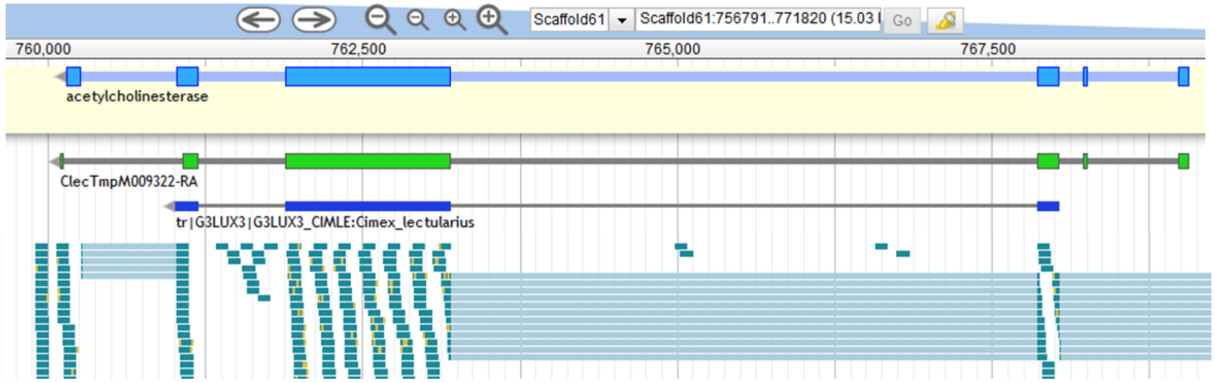

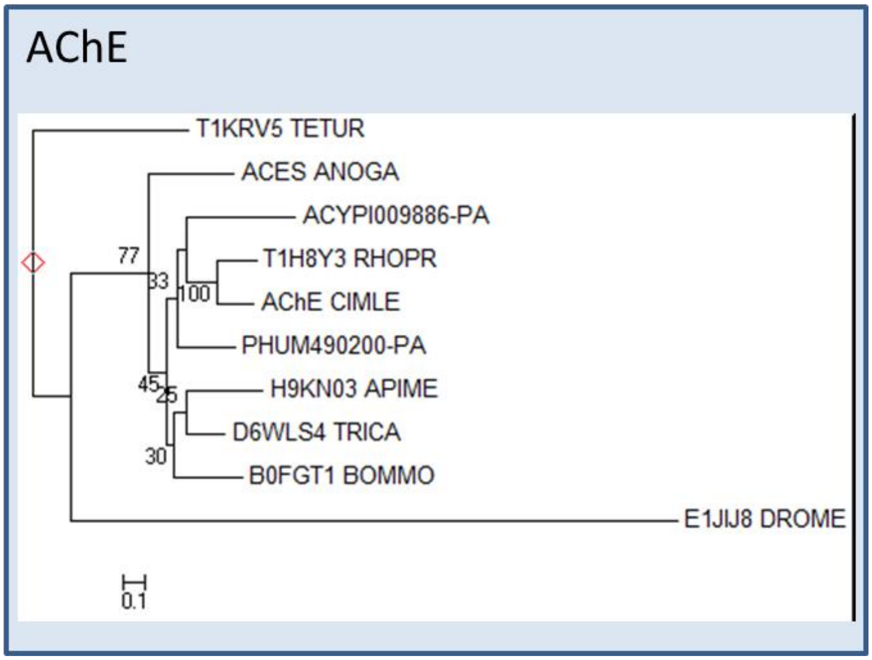

Supplementary Figure 18. A) Acetylcholinesterase manually annotated gene model (blue), predicted gene model (green) with the RNA-seq data below the models. **B)** Acetylcholinesterase phylogenetic tree with outgroups.

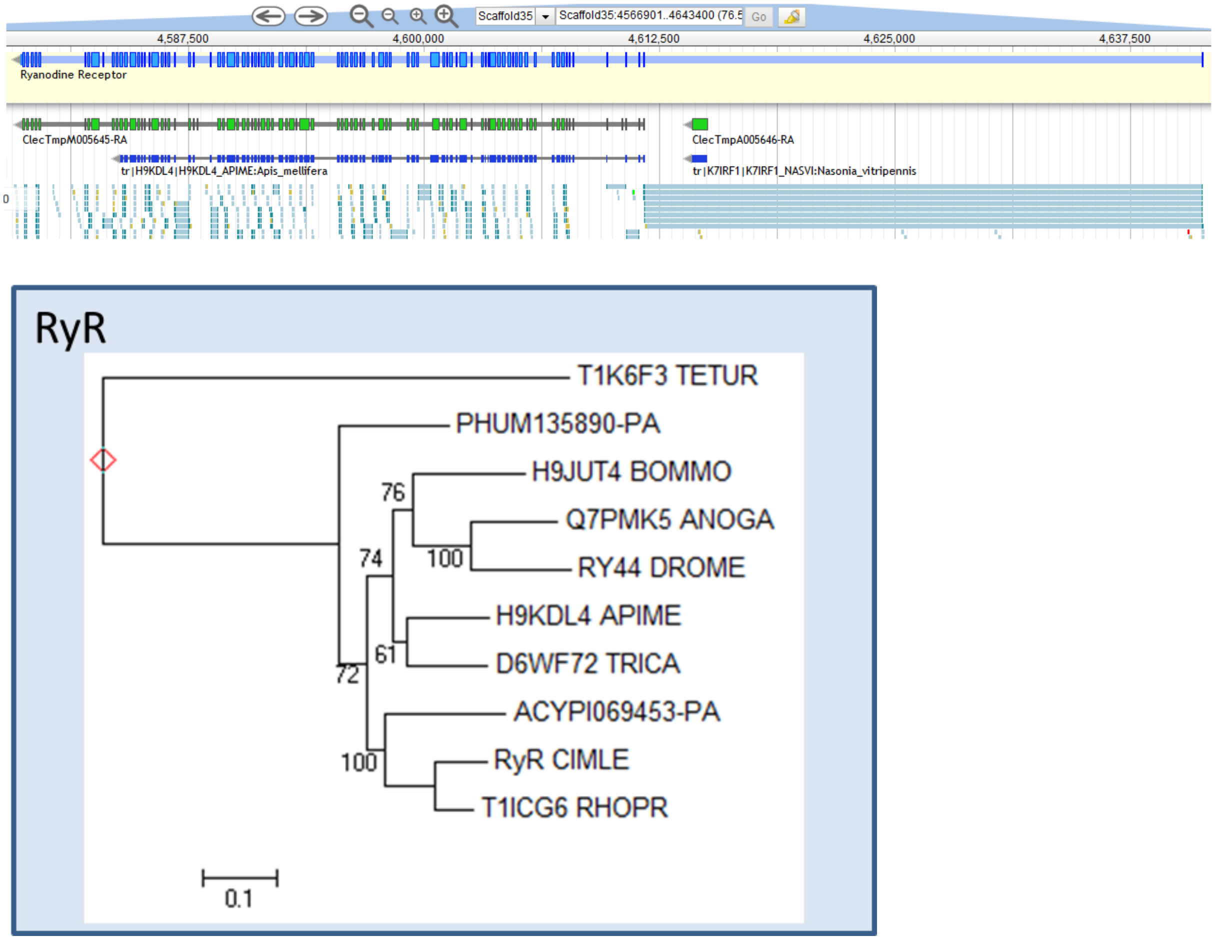

Supplementary Figure 19. A) Ryanodine manually annotated gene model (blue), predicted gene model (green) with the RNA-seq data below the models. **B)** Ryanodine phylogenetic tree with outgroups.

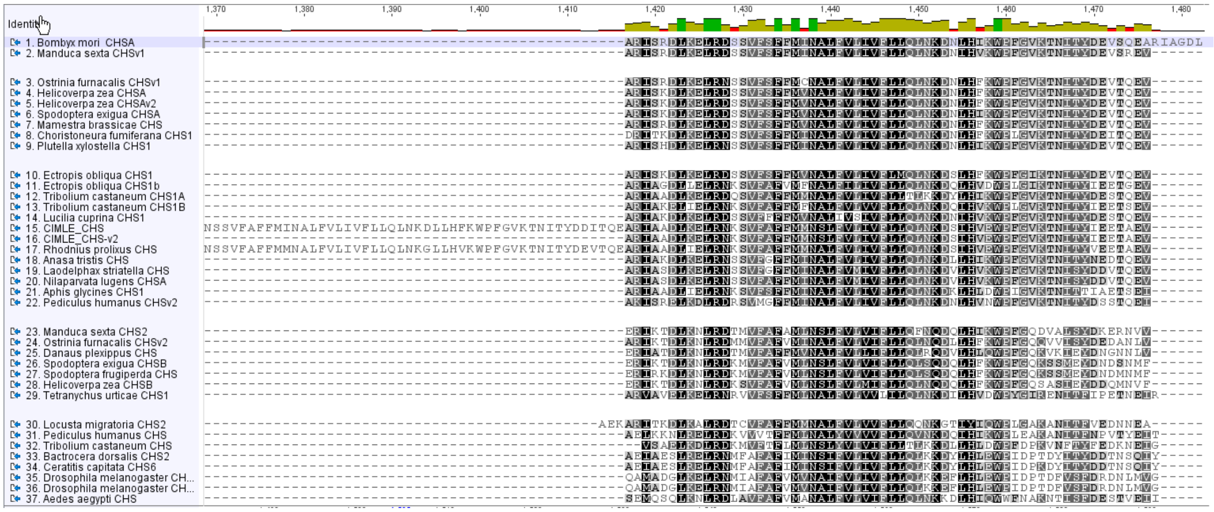
Identity
1. Bombyx mori CHSA
2. Manduca sexta CHSv1
3. Ostrinia furnacalis CHSv1
4. Helicoverpa zea CHSA
5. Helicoverpa zea CHSAv2
6. Spodoptera exigua CHSA
7. Mamestra brassicae CHS
8. Choristoneura fumiferana CHS1
9. Plutella xylostella CHS1
10. Ectropis obliqua CHS1
11. Ectropis obliqua CHS1b
12. Tribolium castaneum CHS1A
13. Tribolium castaneum CHS1B
14. Lucilia cuprina CHS1
15. CIMLE_CHS
16. CIMLE_CHS-v2
17. Rhodnius prolixus CHS
18. Anasa tristis CHS
19. Laodelphax striatella CHS
20. Nilaparvata lugens CHSA
21. Aphis glycines CHS1
22. Pediculus humanus CHSv2
23. Manduca sexta CHS2
24. Ostrinia furnacalis CHSv2
25. Danaus plexippus CHS
26. Spodoptera exigua CHSB
27. Spodoptera frugiperda CHS
28. Helicoverpa zea CHSB
29. Tetranychus urticae CHS1
30. Locusta migratoria CHS2
31. Pediculus humanus CHS
32. Tribolium castaneum CHS
33. Bactrocera dorsalis CHS2
34. Ceratitis capitata CHS6
35. Drosophila melanogaster CH...
36. Drosophila melanogaster CH...
37. Aedes aegypti CHS

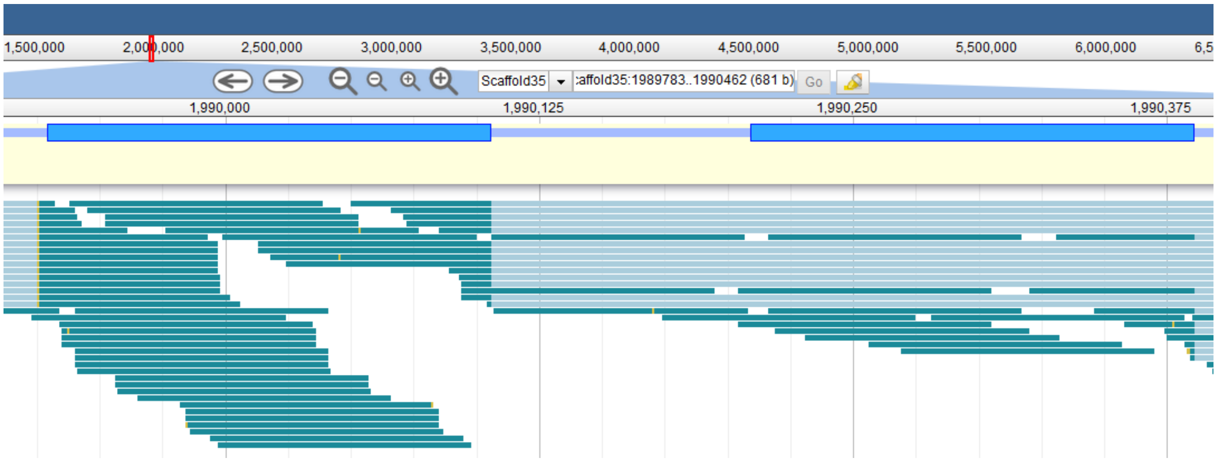
1,500,000
2,000,000
2,500,000
3,000,000
3,500,000
4,000,000
4,500,000
5,000,000
5,500,000
6,000,000
Scaffold35
Go
1,990,000
1,990,125
1,990,250
1,990,375

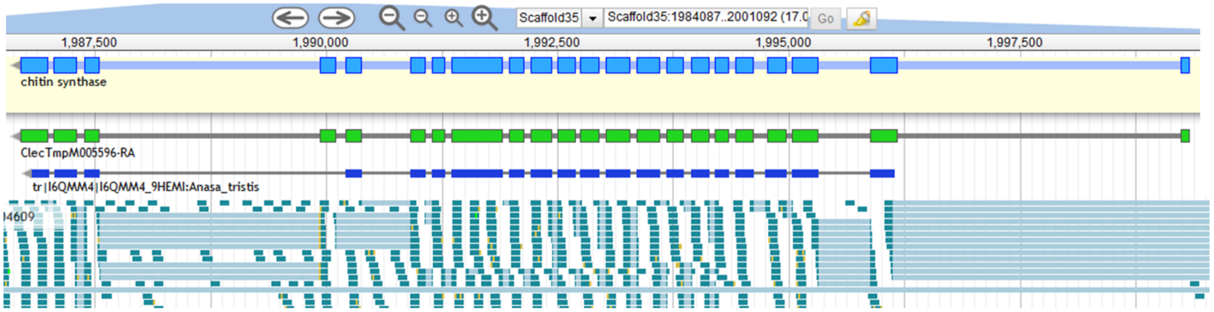
Scaffold35
Scaffold35:1984087..2001092
Go
1,987,500
1,990,000
1,992,500
1,995,000
1,997,500
chitin synthase
ClecTmpM005596-RA
tr|I6QMM4|I6QMM4_9HEMI:Anasa_tristis

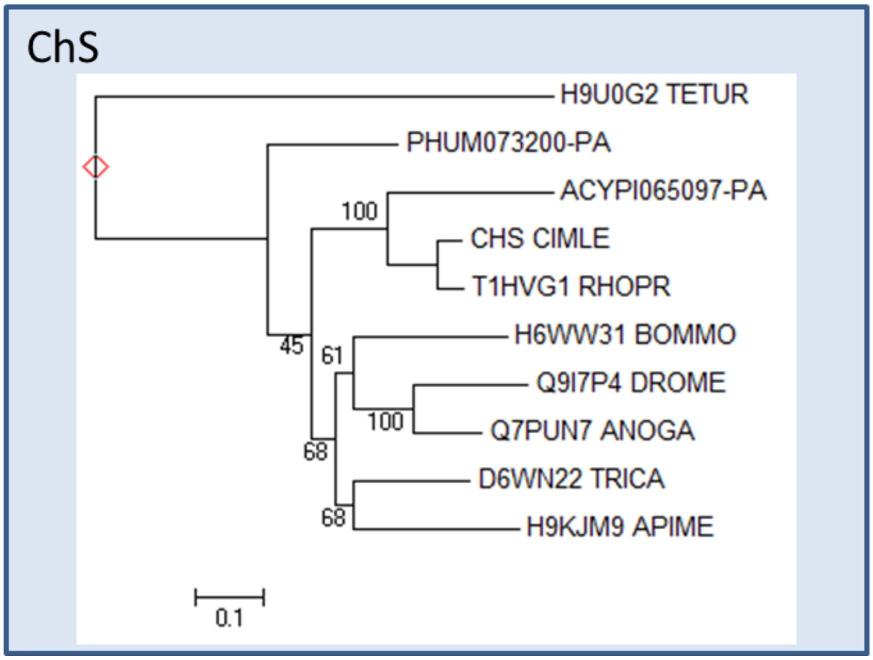

Supplementary Figure 20. **A)** Chitin manually annotated gene model (blue), predicted gene model (green) with the RNA-seq data below the models. **B)** Chitin phylogenetic tree with outgroups.

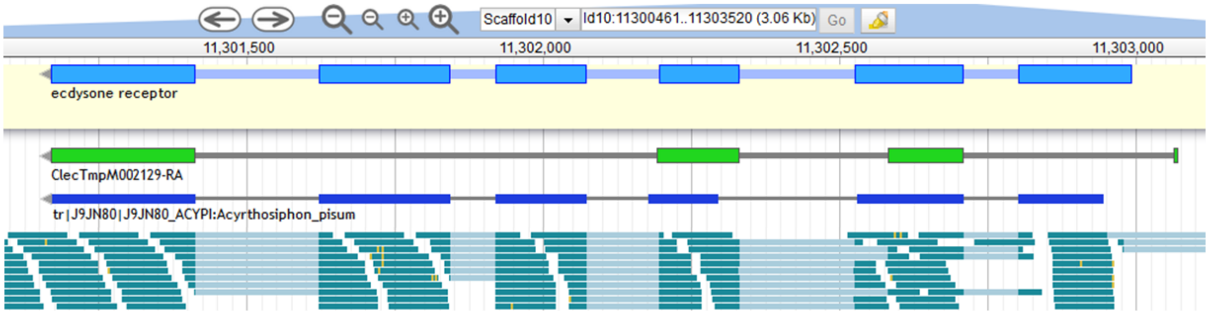

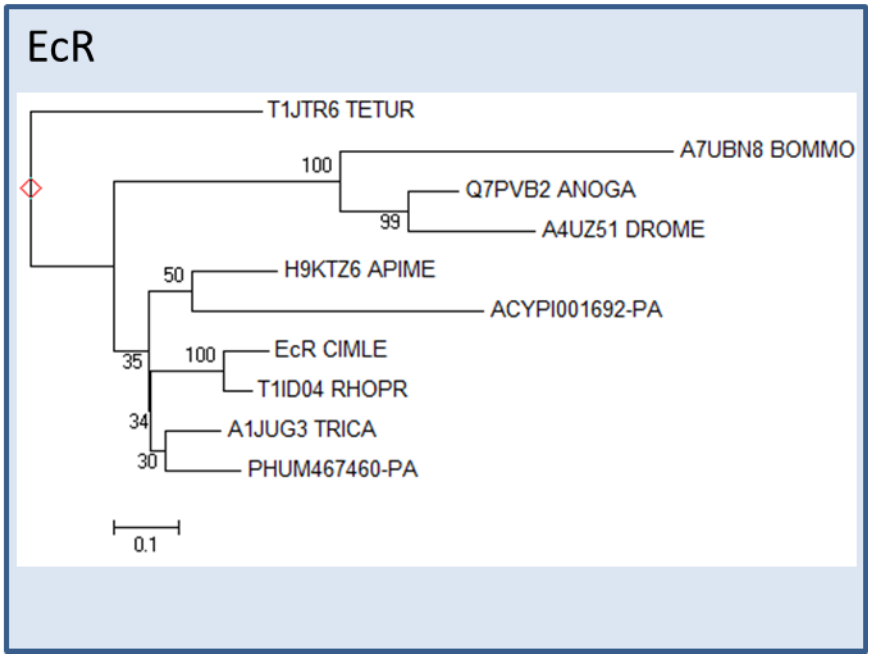

Supplementary Figure 21. A) Ecdysone receptor manually annotated gene model (blue), predicted gene model (green) with the RNA-seq data below the models. **B)** Ecdysone receptor phylogenetic tree with outgroups.

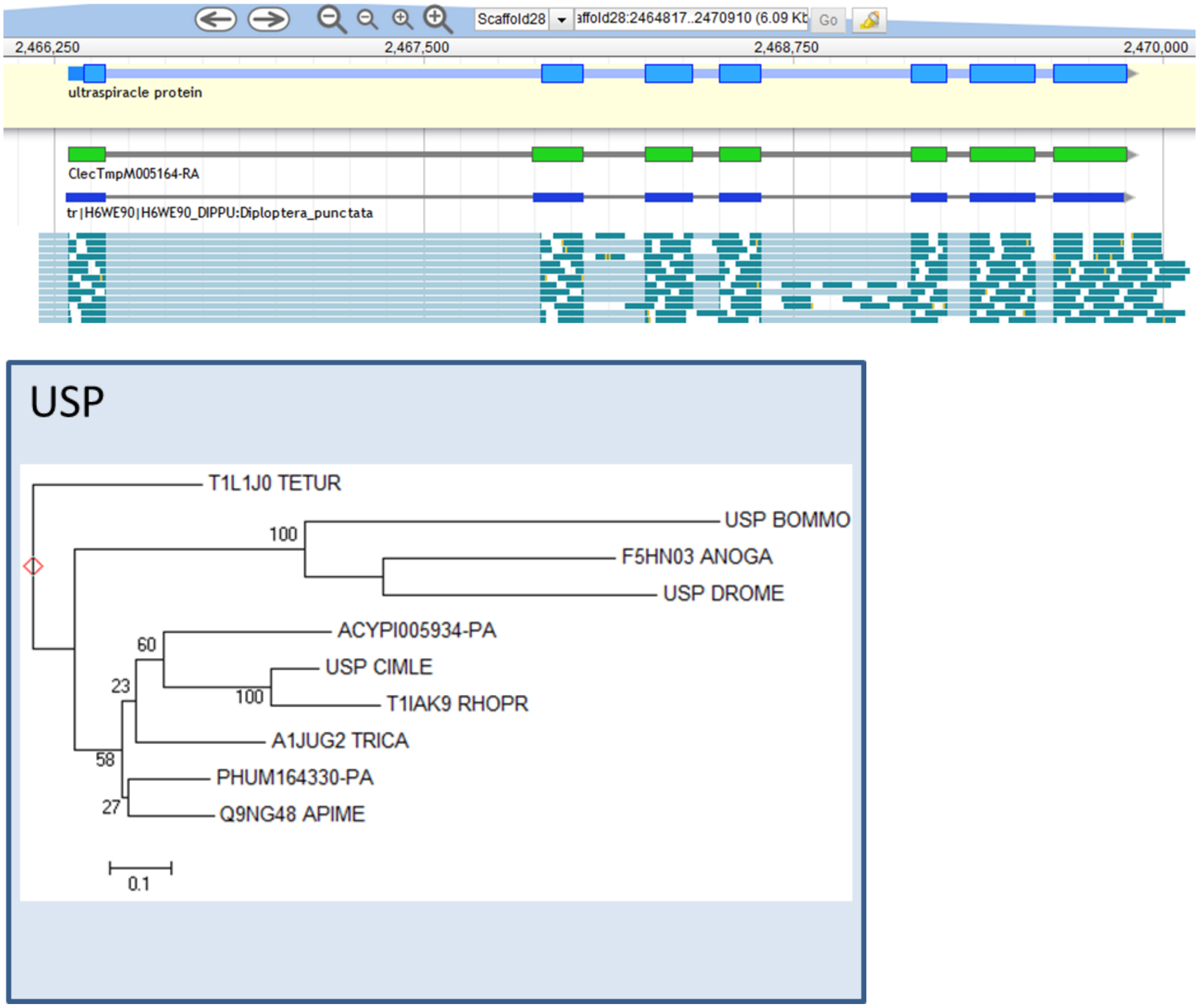

Supplementary Figure 22. **A)** Ultrapiracle manually annotated gene model (blue), predicted gene model (green) with the RNA-seq data below the models. **B)** Ultraspiracle phylogenetic tree with outgroups.

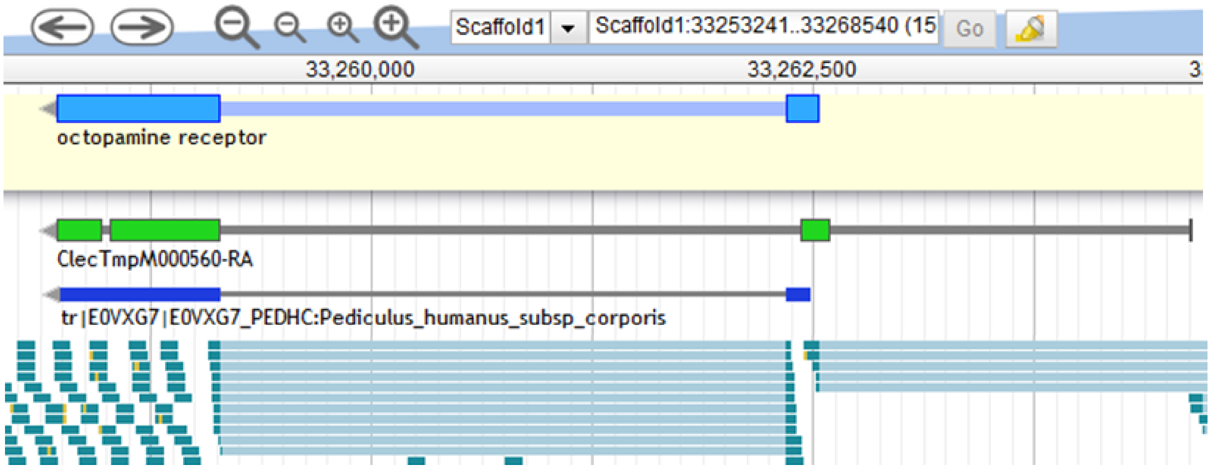

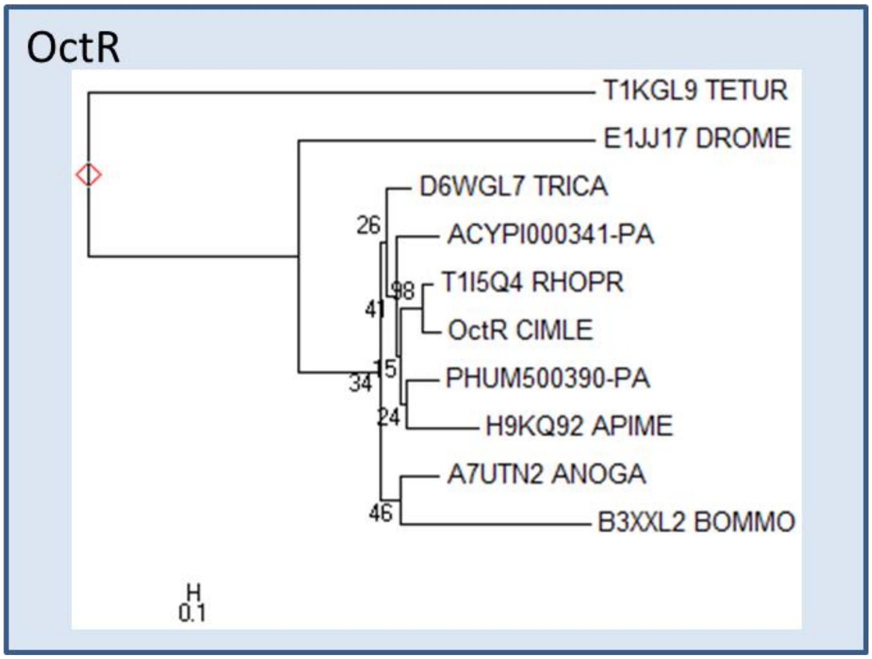

Supplementary Figure 23. **A)** Octopamine receptor manually annotated gene model (blue), predicted gene model (green) with the RNA-seq data below the models. **B)** Octopamine receptor phylogenetic tree with outgroups.

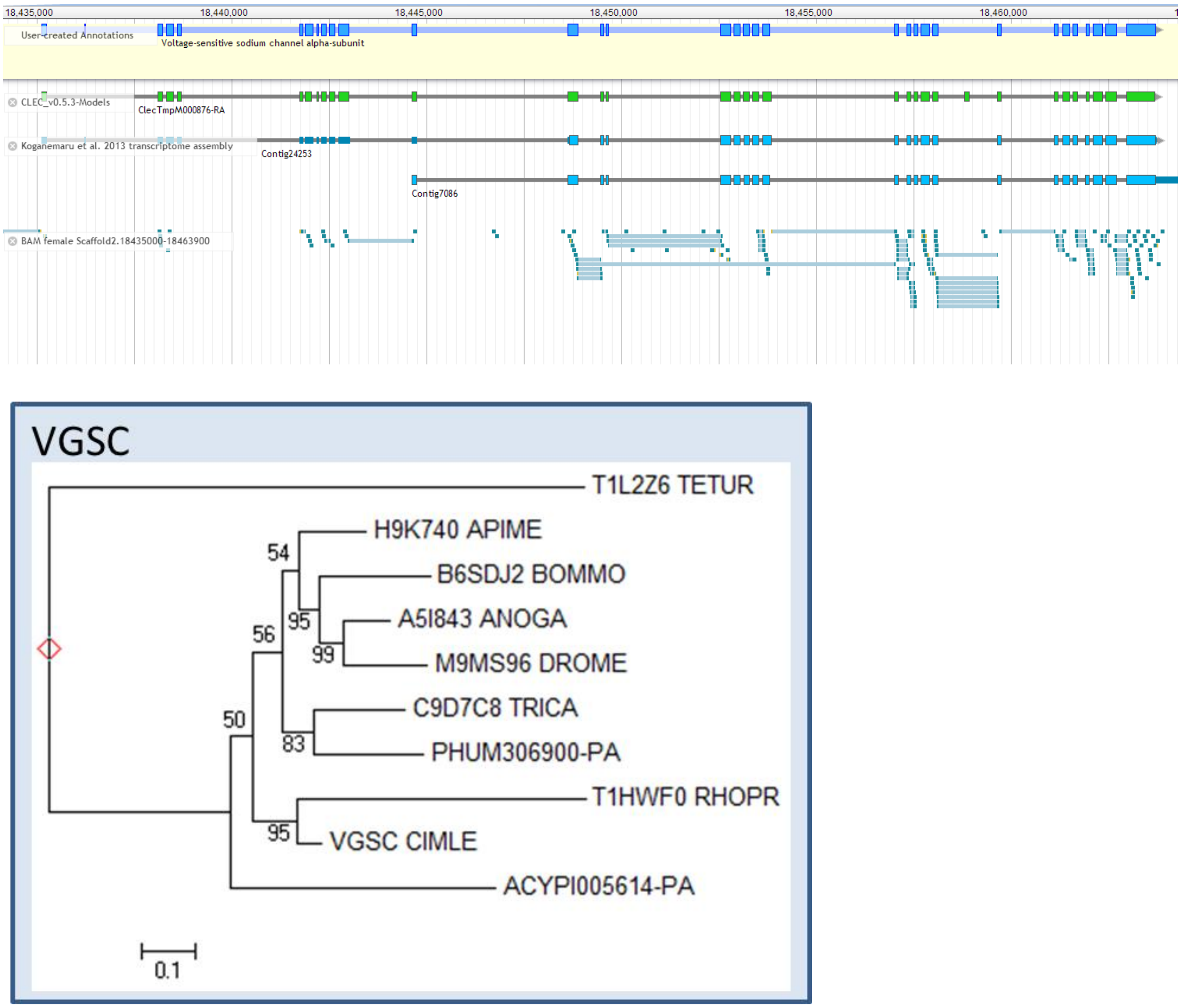

Supplementary Figure 24. A) Voltage-gated sodium channel manually annotated gene model (blue), predicted gene model (green) with the RNA-seq data below the models. **B)** Voltage-gated sodium channel phylogenetic tree with outgroups.

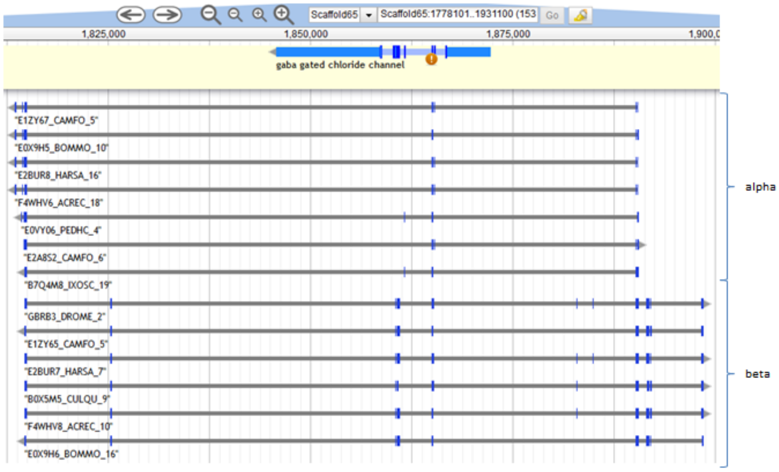

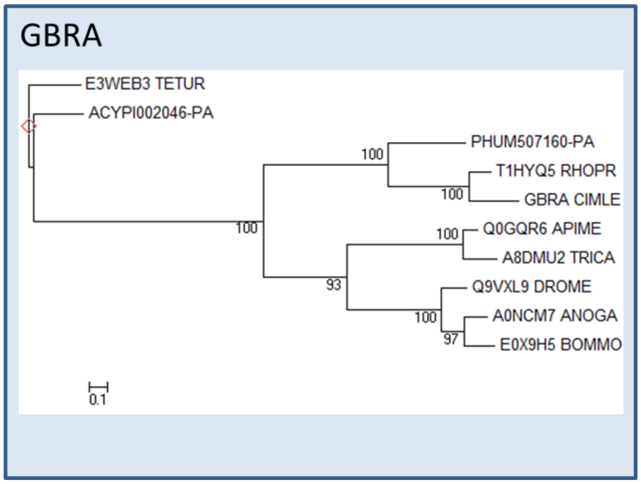

Supplementary Figure 25. **A)** GABA-gated chloride channel manually annotated gene model (blue), predicted gene model (green) with the RNA-seq data below the models. **B)** GABA-gated chloride channel phylogenetic tree with outgroups

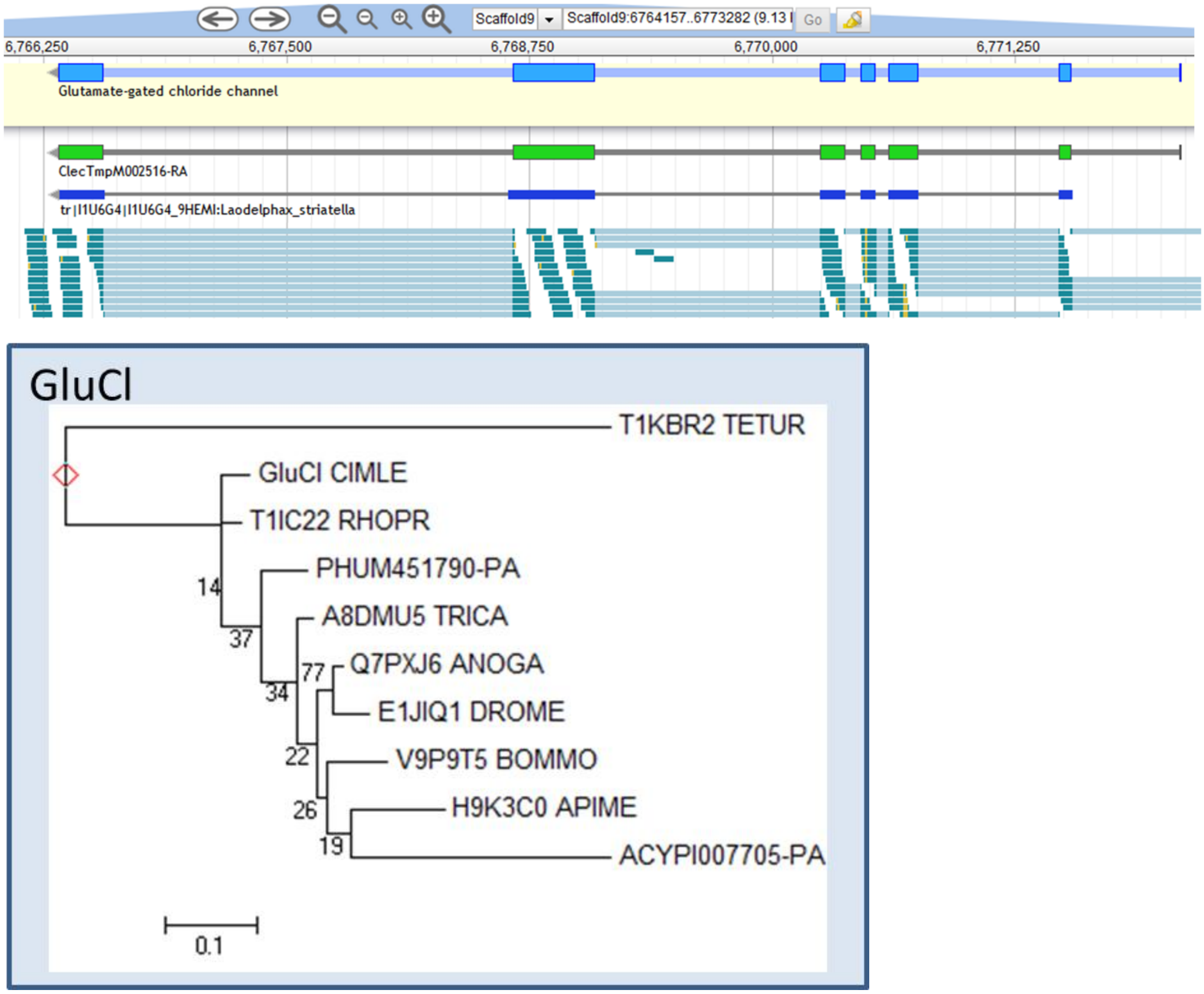

Supplementary Figure 26. **A)** Glutamate-gated chloride channel manually annotated gene model (blue), predicted gene model (green) with the RNA-seq data below the models. **B)** GABA-gated chloride channel phylogenetic tree with outgroups.

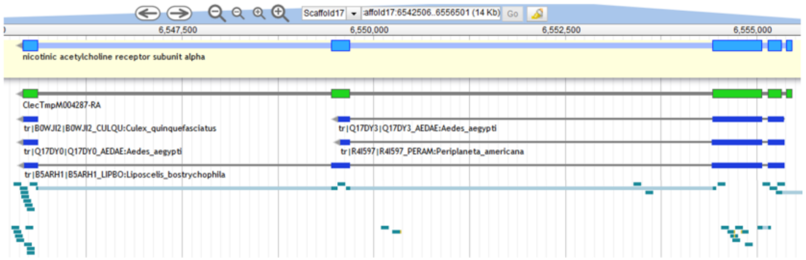

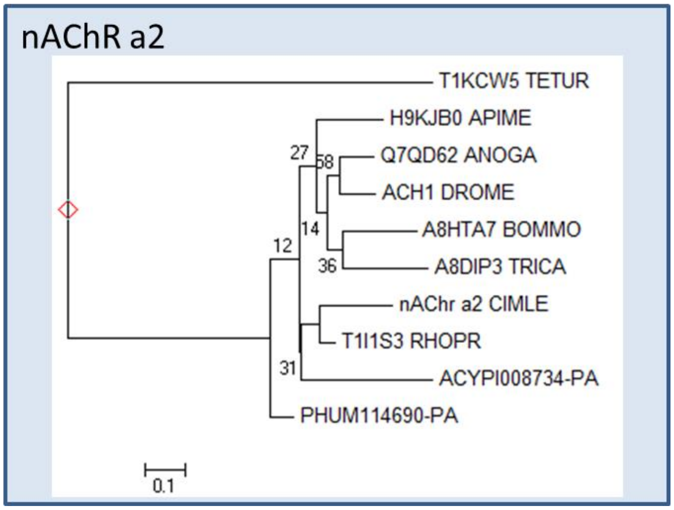

Supplementary Figure 27. **A)** Nicotinic acetylcholine receptor subunit alpha 2 manually annotated gene model (blue), predicted gene model (green) with the RNA-seq data below the models. **B)** Nicotinic acetylcholine receptor subunit alpha 2 phylogenetic tree with outgroups.

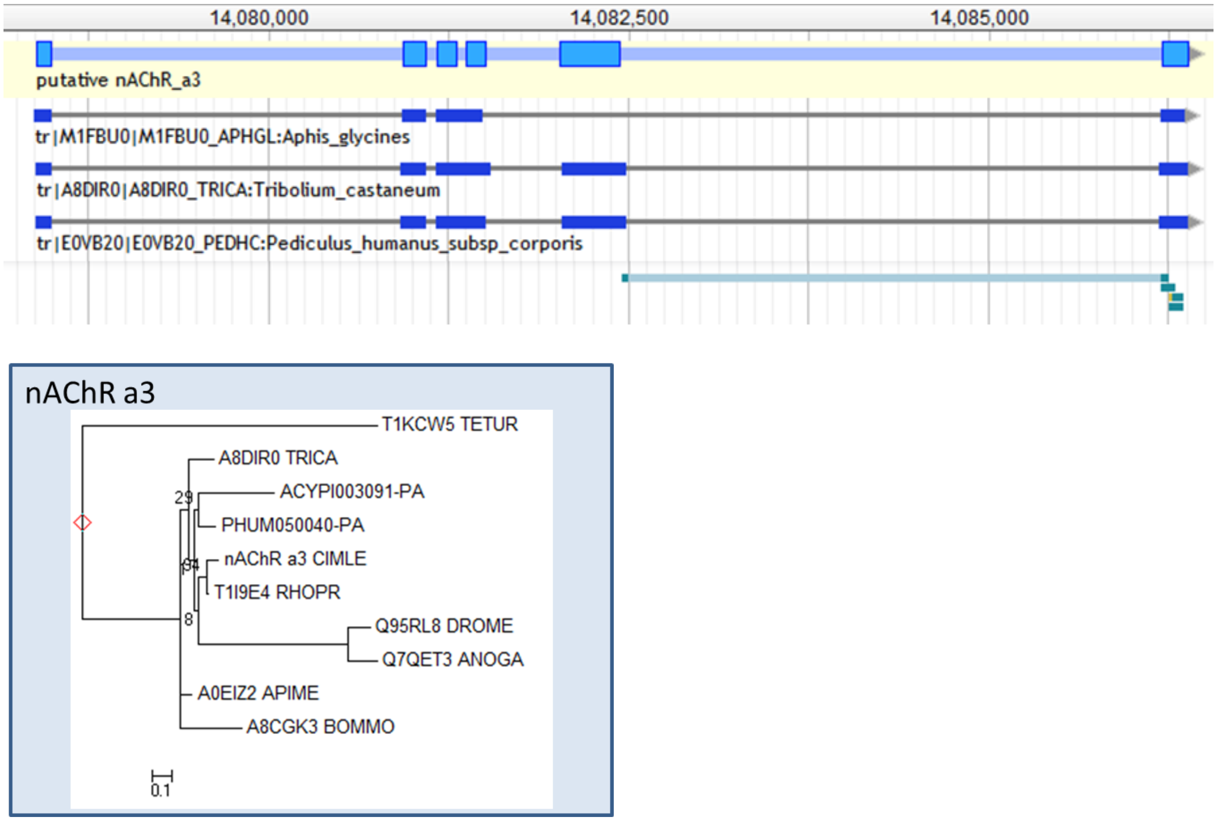

Supplementary Figure 28. **A)** Nicotinic acetylcholine receptor subunit alpha 3 manually annotated gene model (blue), predicted gene model (green) with the RNA-seq data below the models. **B)** Nicotinic acetylcholine receptor subunit alpha 3 phylogenetic tree with outgroups.

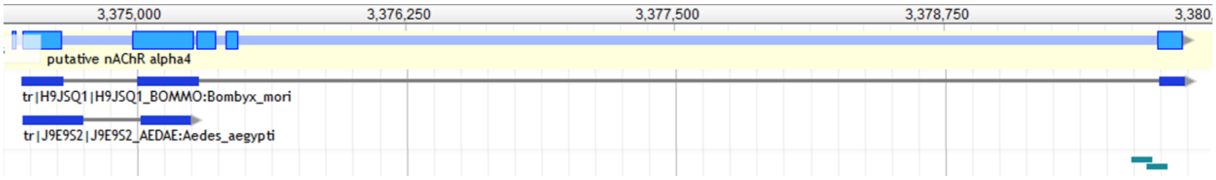

The annotated transcript is not backed by RNA-seq reads.

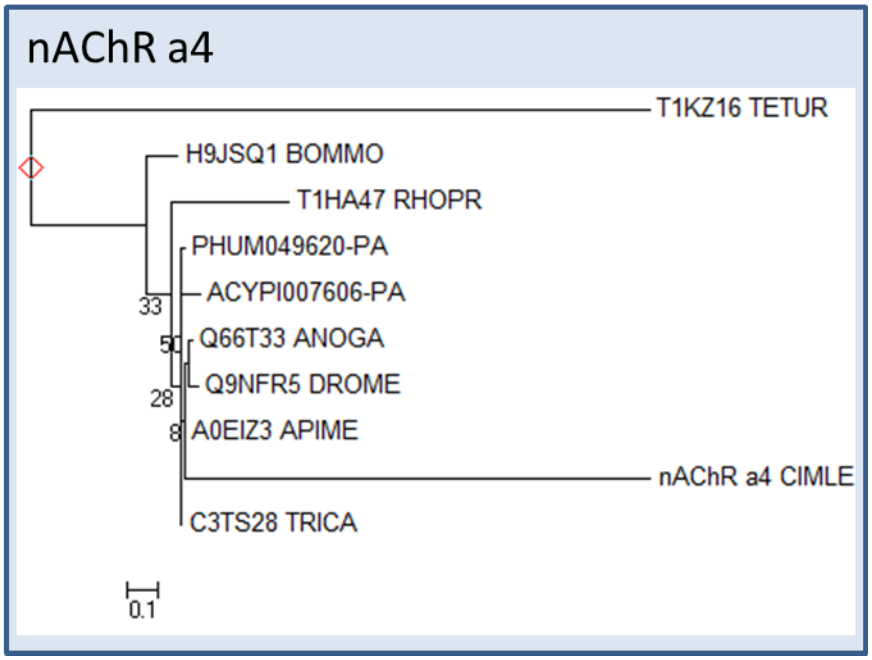

Supplementary Figure 29. A) Nicotinic acetylcholine receptor subunit alpha 4 manually annotated gene model (blue), predicted gene model (green) with the RNA-seq data below the models. **B)** Nicotinic acetylcholine receptor subunit alpha 4 phylogenetic tree with outgroups.

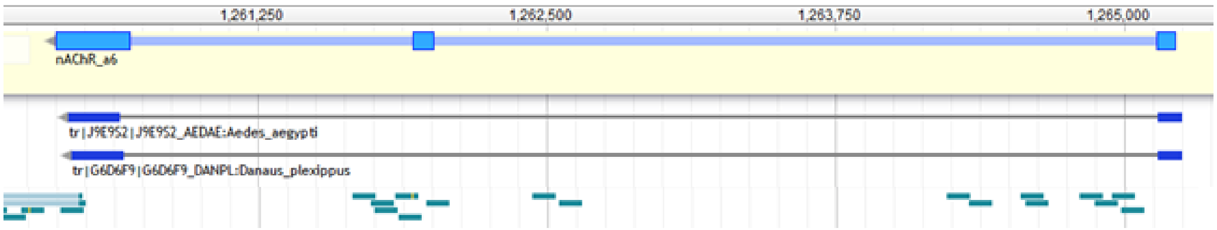

The few RNA-seq reads contradict the exon model which in turn is backed by blast alignments with other arthropod sequences and the exon-intron boundaries (GT-AG-rule).

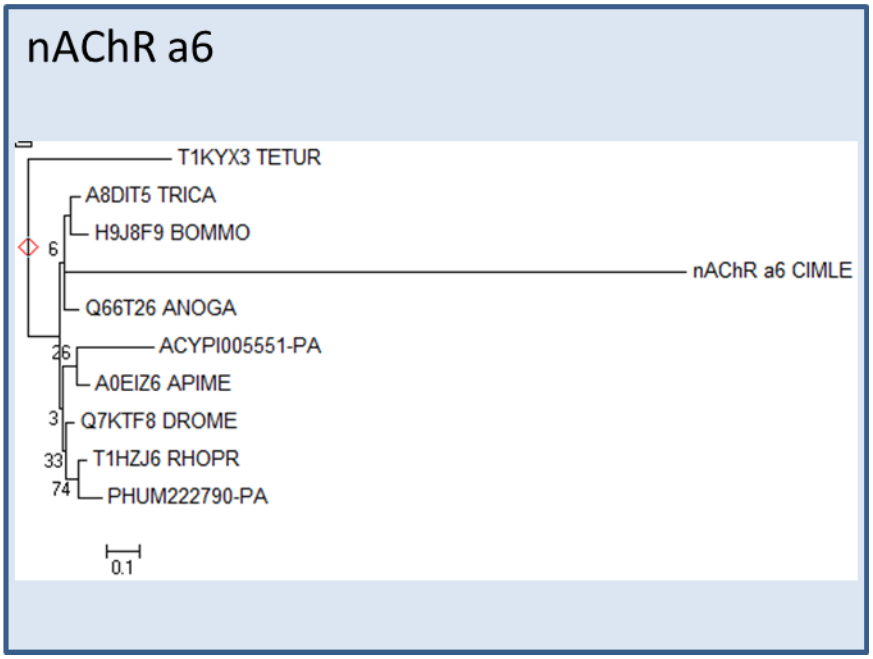

Supplementary Figure 30. A) Nicotinic acetylcholine receptor subunit alpha 6 manually annotated gene model (blue), predicted gene model (green) with the RNA-seq data below the models. **B)** Nicotinic acetylcholine receptor subunit alpha 6 phylogenetic tree with outgroups.

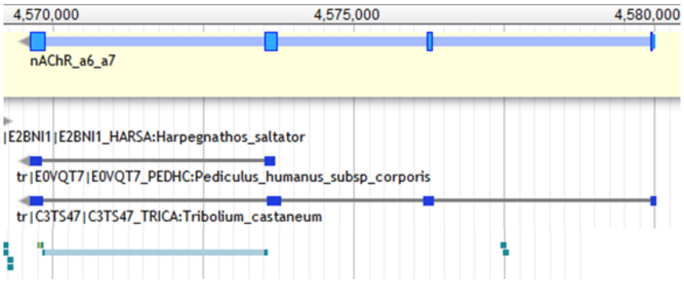

Very few RNA-seq reads support all modeled exons.

The transcript model is 5' incomplete as suggested by multiple alignment with other arthropod alpha subunits.

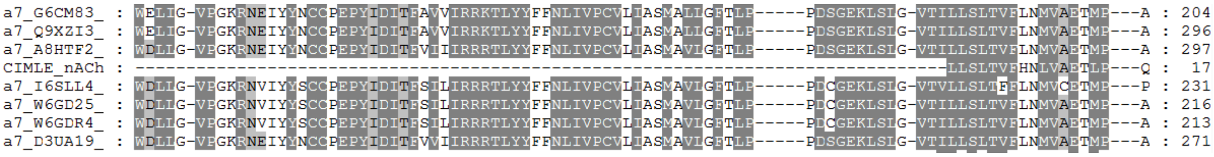

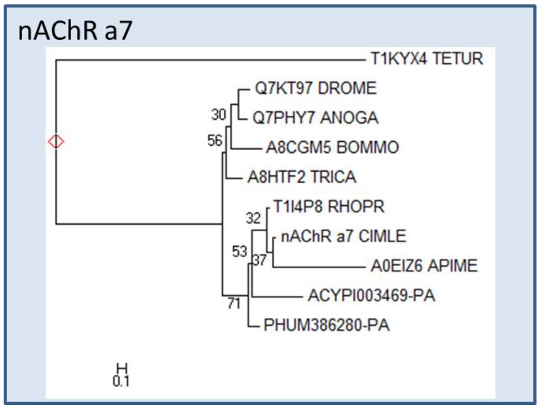

Supplementary Figure 31. A) Nicotinic acetylcholine receptor subunit alpha 7 manually annotated gene model (blue), predicted gene model (green) with the RNA-seq data below the models. **B)** Alignment of alpha 7. **C)** Nicotinic acetylcholine receptor subunit alpha 7 phylogenetic tree with outgroups.

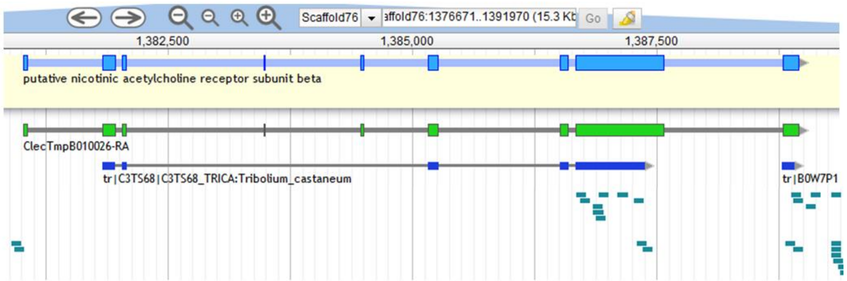

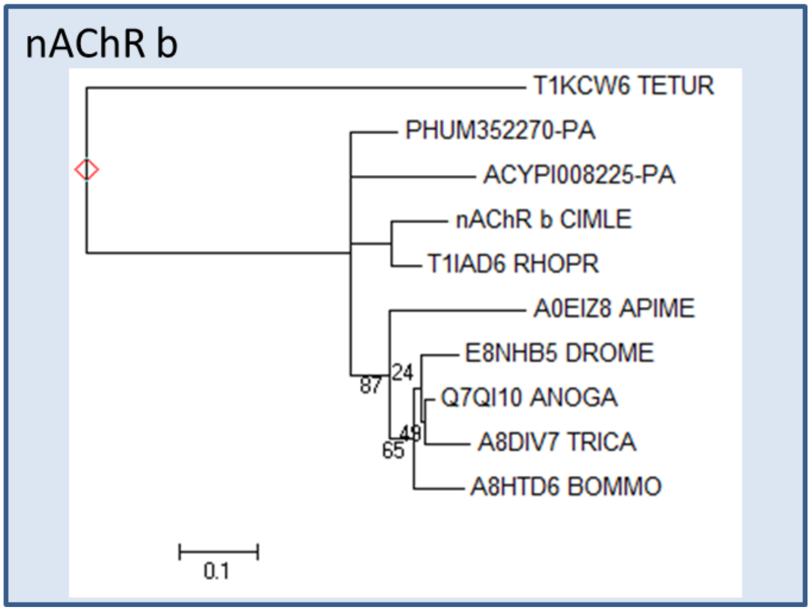

Supplementary Figure 32. A) Nicotinic acetylcholine receptor beta subunit manually annotated gene model (blue), predicted gene model (green) with the RNA-seq data below the models. **B)** Alignment of beta. **C)** Nicotinic acetylcholine receptor beta subunit phylogenetic tree with outgroups.

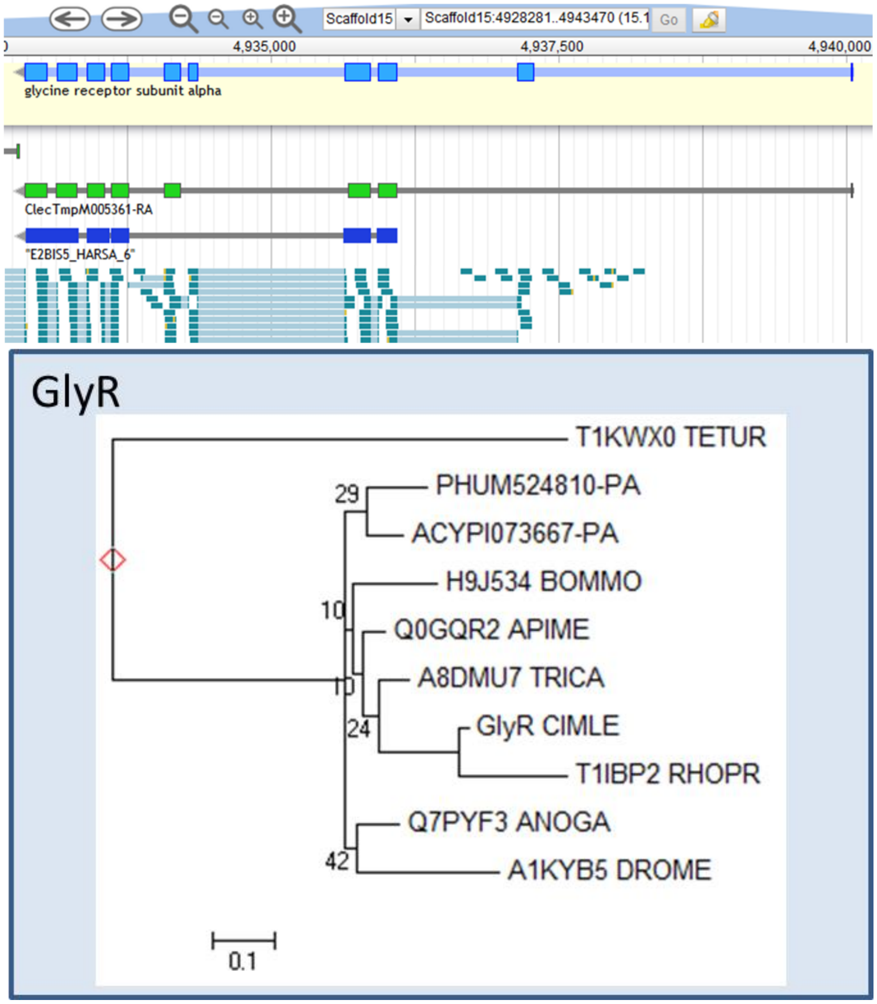

Supplementary Figure 33. **A)** Glycine receptor manually annotated gene model (blue), predicted gene model (green) with the RNA-seq data below the models. **B)** Alignment of glycine receptor. **C)** Glycine receptor phylogenetic tree with outgroups.

core set of insect peptides

Supplementary Figure 33. Core set of neuropeptides. Numbers indicate number of prepropeptide genes for the respective peptide family.

**neuropeptide**	Cimex	Rhodnius	Nilaparvata	Apis	Nasonia	Drosophila	Aedes	Bombyx	Tribolium	Acyrthosiphon
AKH	1	1	1	1	1	1	1	2	2	1
AST-C	1	1	1	1	1	1	1	1	1	1
AST-CC	1	1	1	1	1	1	1	1	1	1
Bursicon alpha	1		1	1	1	1	1	1	1	1
Bursicon beta	1		1	1	1	1	1	1	1	1
CCAP	1	1	1	1	1	1	1	1	1	1
CCHamide-1	1		1	1	1	1	1	1	1	1
CCHamide-2	1	1	1	1	1	1	1	1	1	1
Calcitonin-like DH	1	1	1	1	1	1	1	1	1	1
CRF-like DH	1	1	1	1	1	1	1	1	1	1
EH	1	1	2	1	1	1	5	1	1	3
ETH	1		1	1	1	1	1	1	1	1
ILP-B	1		1	1	1	5	6	38	2	7
ITP	1	1	1	1	1	1	1	1	1	1
Myosuppressin	1	1	1	1	1	1	1	1	1	1
Pyrokinin	1	1	1	1	1	1	1	1	1	1
SIFamide	1	1	1	1	1	1	1	2	1	1
sNPF	1	1	1	1	1	1	1	1	1	1
Tachykinin	1	1	1	1	1	1	1	1	1	1

Supplementary Figure 34. Core set of neuropeptides. Numbers indicate the number of prepropeptide genes for the respective peptide family.

**neuropeptide**	Cimex	Rhodnius	Nilaparvata	Acyrthosiphon	Apis	Nasonia	Drosophila	Aedes	Bombyx	Tribolium
ACP	yes		yes	no	no	yes	no	yes	yes	yes
Allatotropin	yes	yes	yes	yes	yes	yes	no	yes	yes	yes
AST-A	yes	yes	yes	yes	yes	yes	yes	yes	yes	no
Capa	yes	yes	yes	yes	yes	no	yes	yes	yes	yes
CNMa	yes	yes			yes	yes	yes	yes	no	yes
Corazonin	yes	yes	yes	no	yes	yes	yes	yes	yes	no
EFLamide	yes		no							
Elevenin	yes		yes				no		no	
FMRFamide	yes	yes	yes	yes	yes	no	yes	yes	yes	yes
GPA	yes		yes	yes	no	no	yes	yes	yes	yes
GPB	yes		yes	yes	no	no	yes	yes	yes	yes
ILP-A	no		yes	no	no	no	yes	yes	no	yes
ILP-C	?	?	yes	yes	yes	yes	yes	yes	no	yes
Inotocin	no		no	no	no	yes	no	no	no	yes
Kinin	yes	yes	yes	yes	no	no	yes	yes	yes	no
MIP	yes	yes	yes	yes	no	no	yes	yes	yes	yes
Natalisin	yes	yes	yes	yes			yes	yes	yes	yes
Neuroparsin	yes	yes	yes	no	yes	yes	no	yes	yes	yes
NPF	yes	yes	yes	yes	yes	yes	yes	yes	yes	no
NPLP-1	yes	yes	yes	yes	yes	no	yes	yes	yes	yes
NPLP-2	no		no	no	yes	no	yes	no	no	no
NPLP-3	yes		yes	no	yes	no	yes	no	no	no
NPLP-4	yes		yes	no	no	no	yes	no	no	no
Orcokinin	yes	yes	yes	yes	yes	yes	no	yes	yes	no
PDF	yes	yes	yes	no	yes	yes	yes	yes	yes	no
Proctolin	yes	yes	yes	yes	no	no	yes	no	no	yes
PTTH	yes	yes	yes	no	no	yes	yes	yes	yes	yes
RYamide	yes		yes	yes	yes	yes	yes	yes	yes	yes
Sulfakinin	yes	yes	yes	no	yes	no	yes	yes	yes	yes
Trissin	no		no				yes	yes	yes	

Supplementary Figure 35. Variable set of insect neuropeptides. Yes/no indicates occurrence or absence of respective prepropeptide gene.

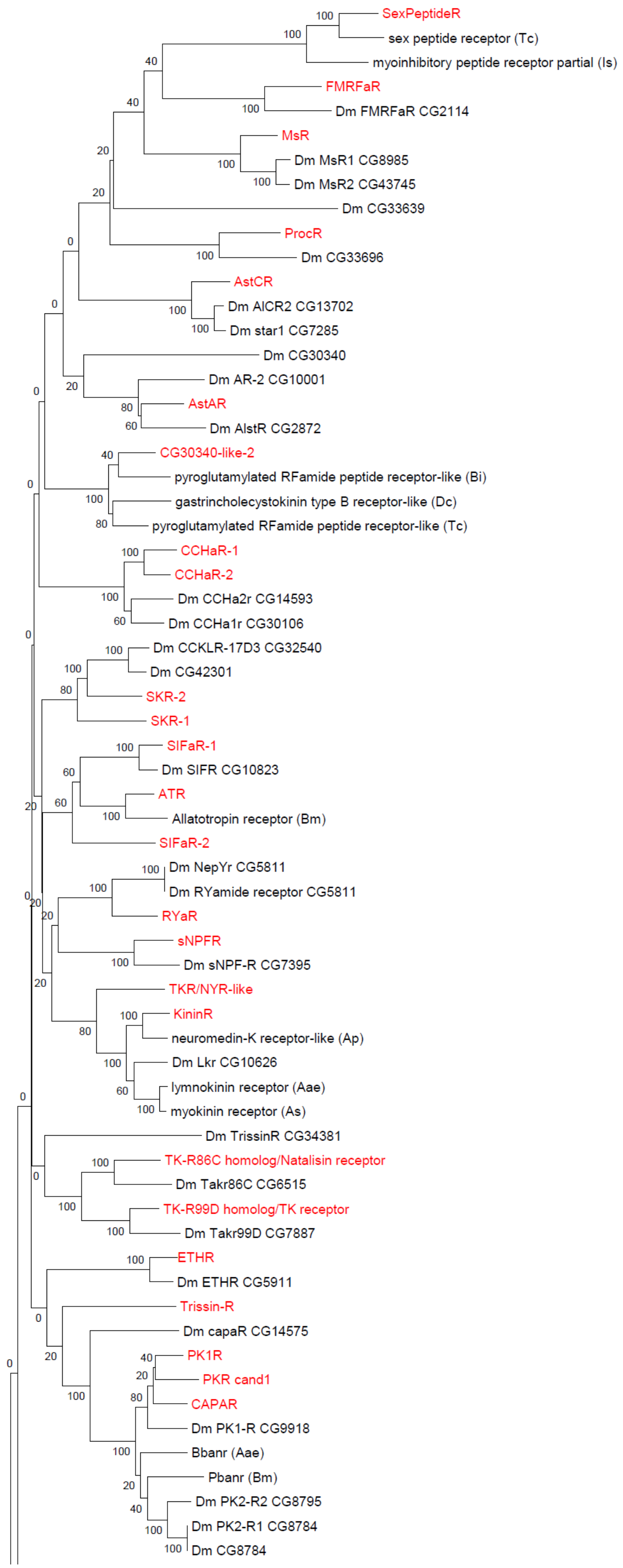
SexPeptideR
sex peptide receptor (Tc)
myoinhibitory peptide receptor partial (Is)
FMRFaR
Dm FMRFaR CG2114
MsR
Dm MsR1 CG8985
Dm MsR2 CG43745
Dm CG33639
ProcR
Dm CG33696
AstCR
Dm AlCR2 CG13702
Dm star1 CG7285
Dm CG30340
Dm AR-2 CG10001
AstAR
Dm AlstR CG2872
CG30340-like-2
pyroglutamylated RFamide peptide receptor-like (Bi)
gastrincholecystokinin type B receptor-like (Dc)
pyroglutamylated RFamide peptide receptor-like (Tc)
CCHaR-1
CCHaR-2
Dm CCHa2r CG14593
Dm CCHa1r CG30106
Dm CCKLR-17D3 CG32540
Dm CG42301
SKR-2
SKR-1
SIFaR-1
Dm SIFR CG10823
ATR
Allatotropin receptor (Bm)
SIFaR-2
Dm NepYr CG5811
Dm RYamide receptor CG5811
RYaR
sNPFR
Dm sNPF-R CG7395
TKR/NYR-like
KininR
neuromedin-K receptor-like (Ap)
Dm Lkr CG10626
lymnokinin receptor (Aae)
myokinin receptor (As)
Dm TrissinR CG34381
TK-R86C homolog/Natalisin receptor
Dm Takr86C CG6515
TK-R99D homolog/TK receptor
Dm Takr99D CG7887
ETHR
Dm ETHR CG5911
Trissin-R
Dm capaR CG14575
PK1R
PKR cand1
CAPAR
Dm PK1-R CG9918
Bbanr (Aae)
Pbanr (Bm)
Dm PK2-R2 CG8795
Dm PK2-R1 CG8784
Dm CG8784

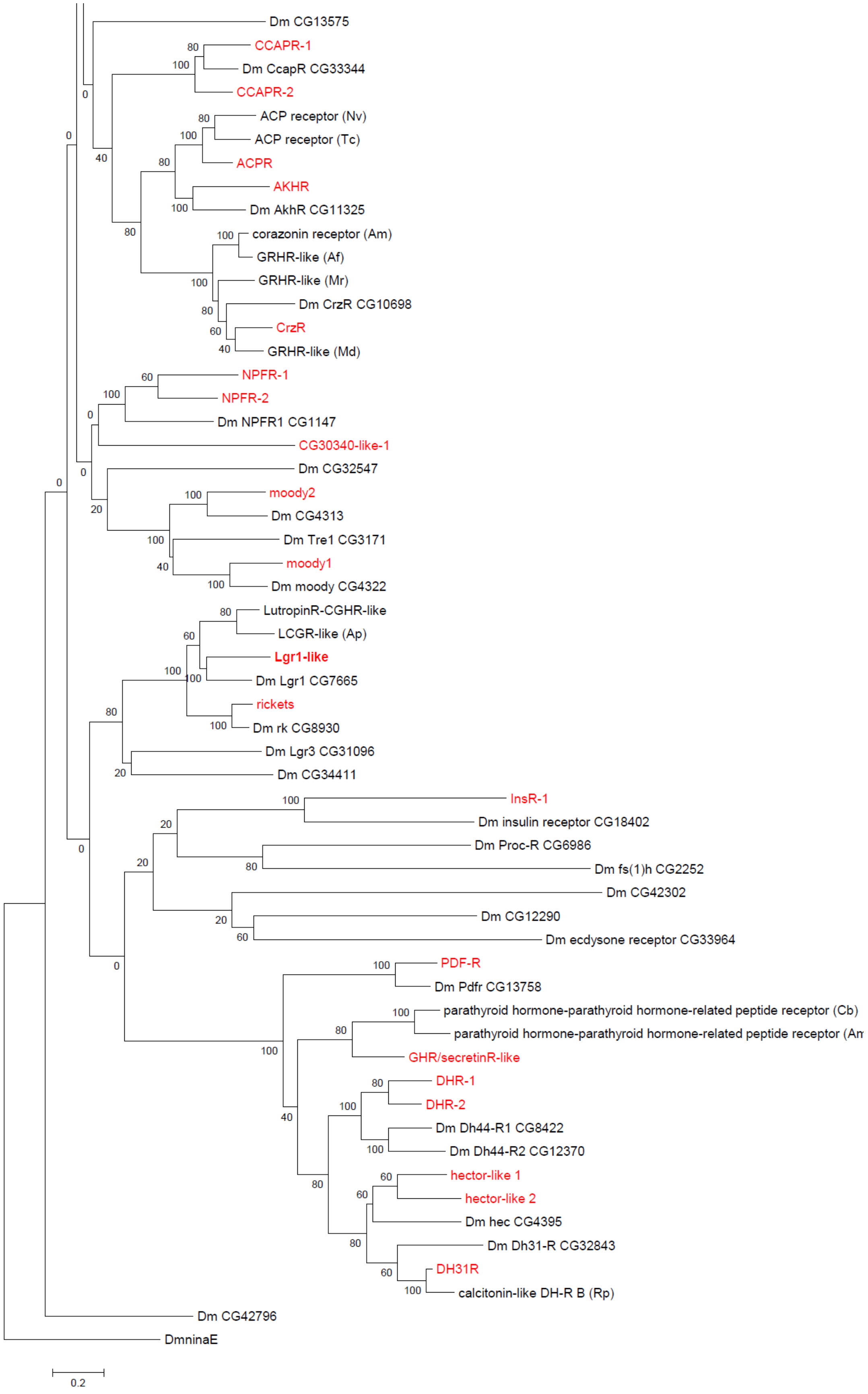
Dm CG13575
CCAPR-1
Dm CcapR CG33344
CCAPR-2
ACP receptor (Nv)
ACP receptor (Tc)
ACPR
AKHR
Dm AkhR CG11325
corazonin receptor (Am)
GRHR-like (Af)
GRHR-like (Mr)
Dm CrzR CG10698
CrzR
GRHR-like (Md)
NPFR-1
NPFR-2
Dm NPFR1 CG1147
CG30340-like-1
Dm CG32547
moody2
Dm CG4313
Dm Tre1 CG3171
moody1
Dm moody CG4322
LutropinR-CGHR-like
LCGR-like (Ap)
Lgr1-like
Dm Lgr1 CG7665
rickets
Dm rk CG8930
Dm Lgr3 CG31096
Dm CG34411
InsR-1
Dm insulin receptor CG18402
Dm Proc-R CG6986
Dm fs(1)h CG2252
Dm CG42302
Dm CG12290
Dm ecdysone receptor CG33964
PDF-R
Dm Pdfr CG13758
parathyroid hormone-parathyroid hormone-related peptide receptor (Cb)
GHR/secretinR-like
DHR-1
DHR-2
Dm Dh44-R1 CG8422
Dm Dh44-R2 CG12370
hector-like 1
hector-like 2
Dm hec CG4395
Dm Dh31-R CG32843
DH31R
calcitonin-like DH-R B (Rp)
Dm CG42796
DmninaE
0.2

Supplementary Figure 36. Phylogenetic tree of peptide GPCR constructed using neighbor-joining method implemented with MEGA5[5]. *C*. *lectularius* receptors are marked in red.

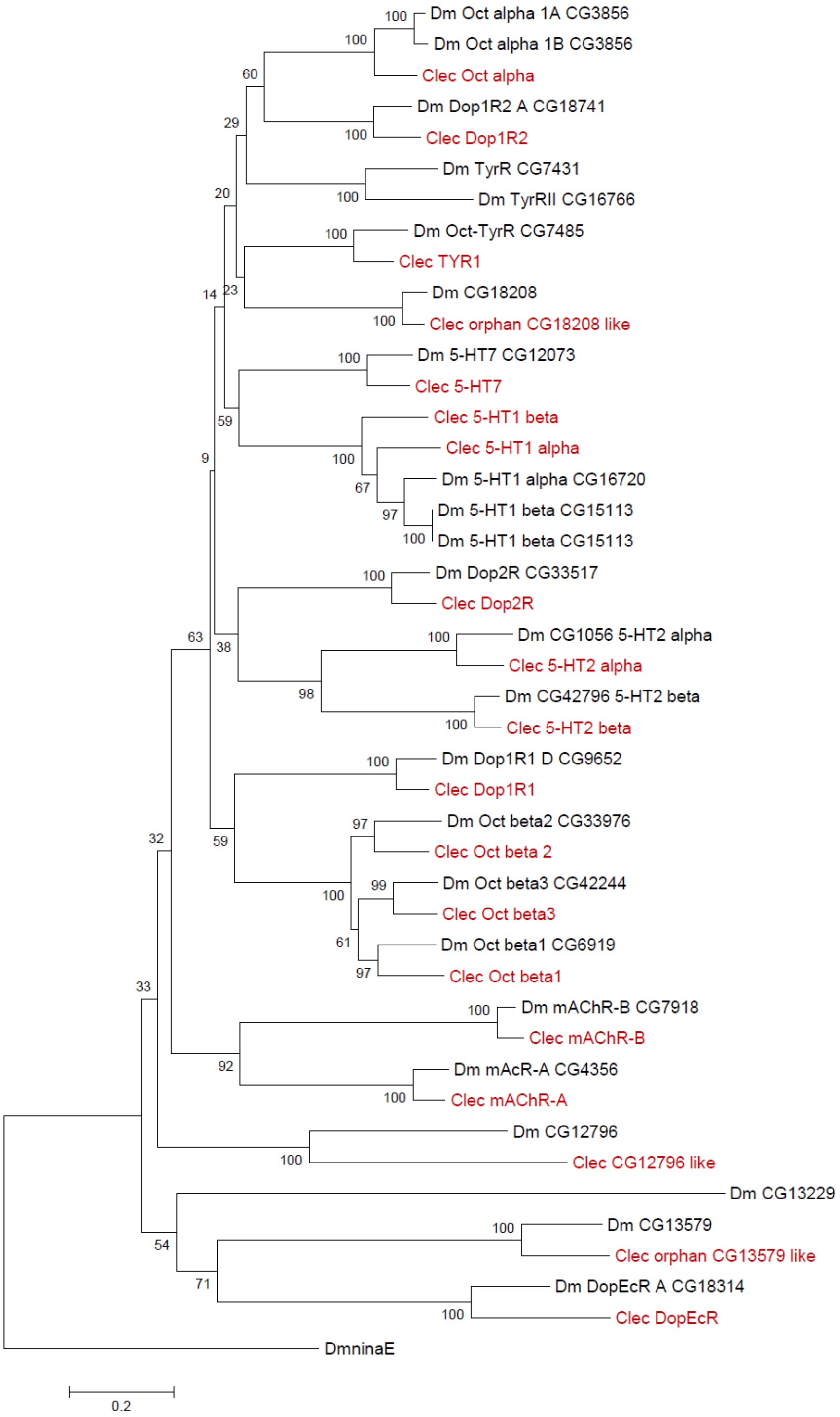

Supplementary Figure 37. Phylogenetic tree of biogenic amine GPCR constructed using neighbor-joining method implemented in MEGA5[5]. *C. lectularius* receptors are marked in red.

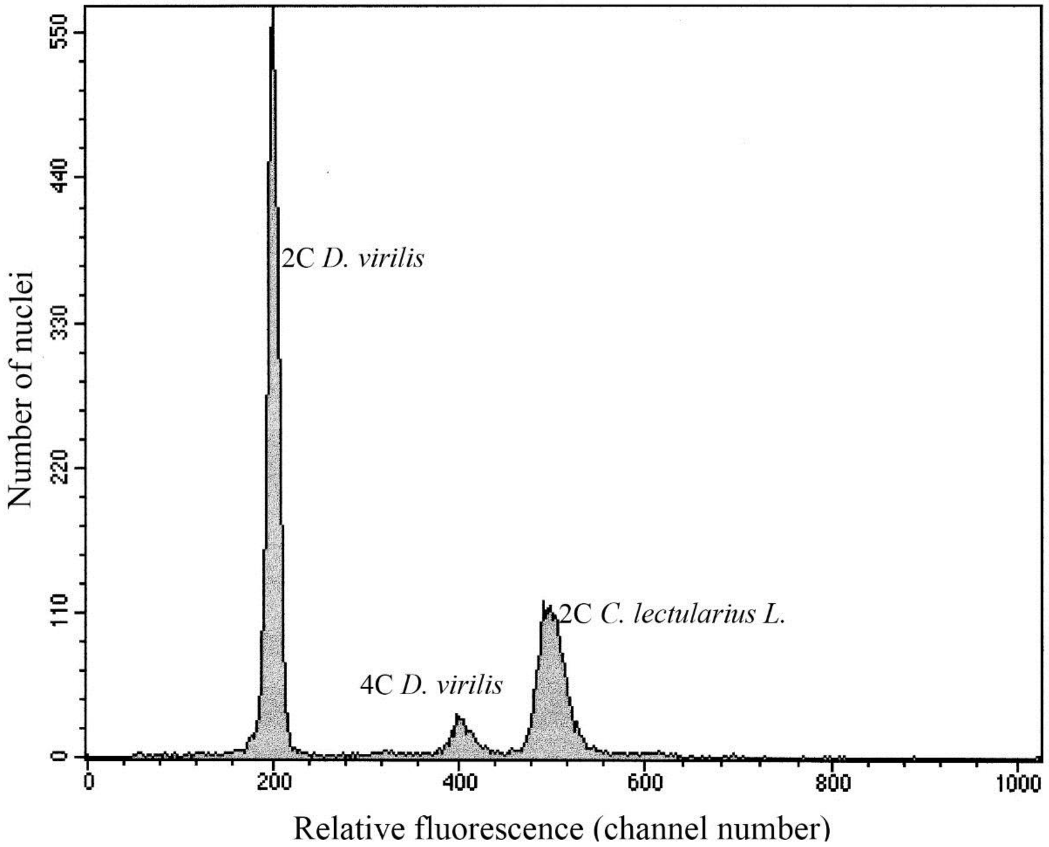

Supplementary Figure 38. Diagram showing differing levels of red fluorescence corresponding to binding of propidium iodide to 2C nuclei from female *Cimex lectularius* compared to 2C and 4C nuclei of *Drosophila virilis*.

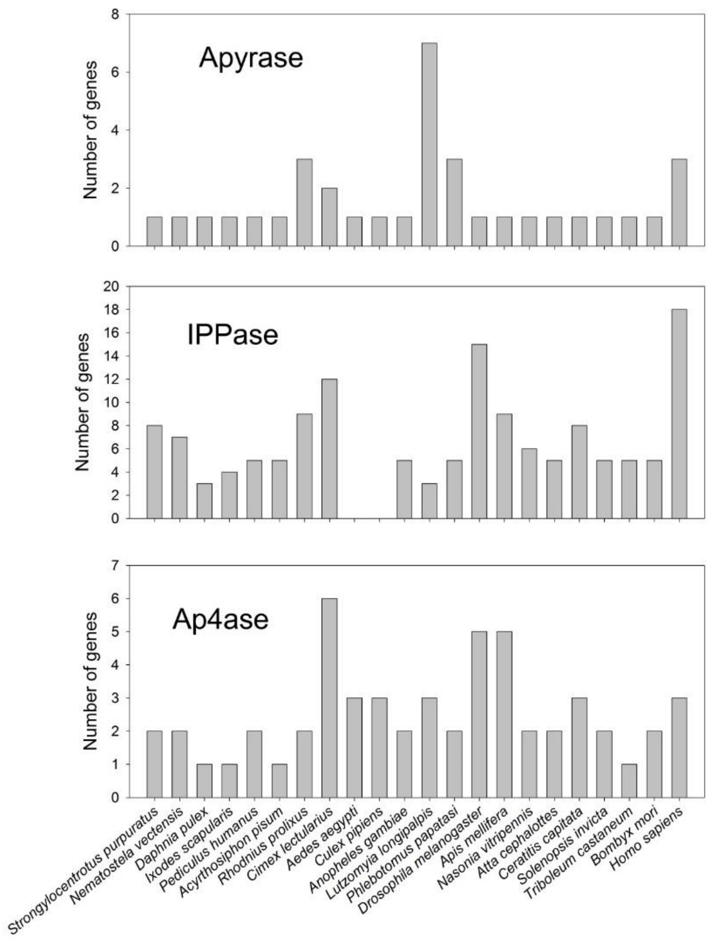

Supplementary Figure 39. Gene family expansions associated with salivary function in *Cimex lectularius*. The graphs indicate the number of genes coding for members of the *Cimex*-type apyrase, inositol polyphosphate phosphatase (IPPase) or diadenosine tetraphosphate hydrolase (Ap4Ase) in the indicated genomes.

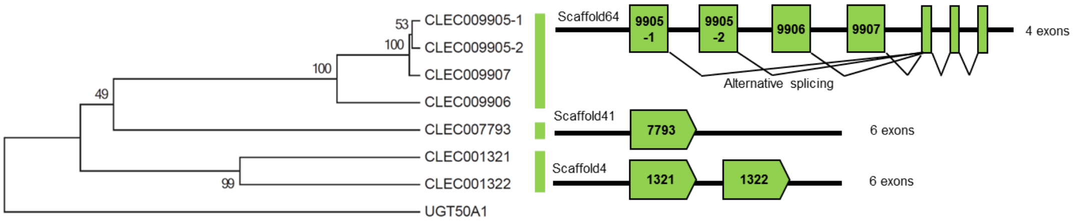

Supplementary Figure 40. The bed bug UGTs and the genomic orientation. The phylogeny was inferred by using the Maximum-Likelihood method based on the JTT matrix-based model. Bootstrap value was 1,000. UGT50A1 (NP_001243994.1) from *B. mori* was used as an outgroup.

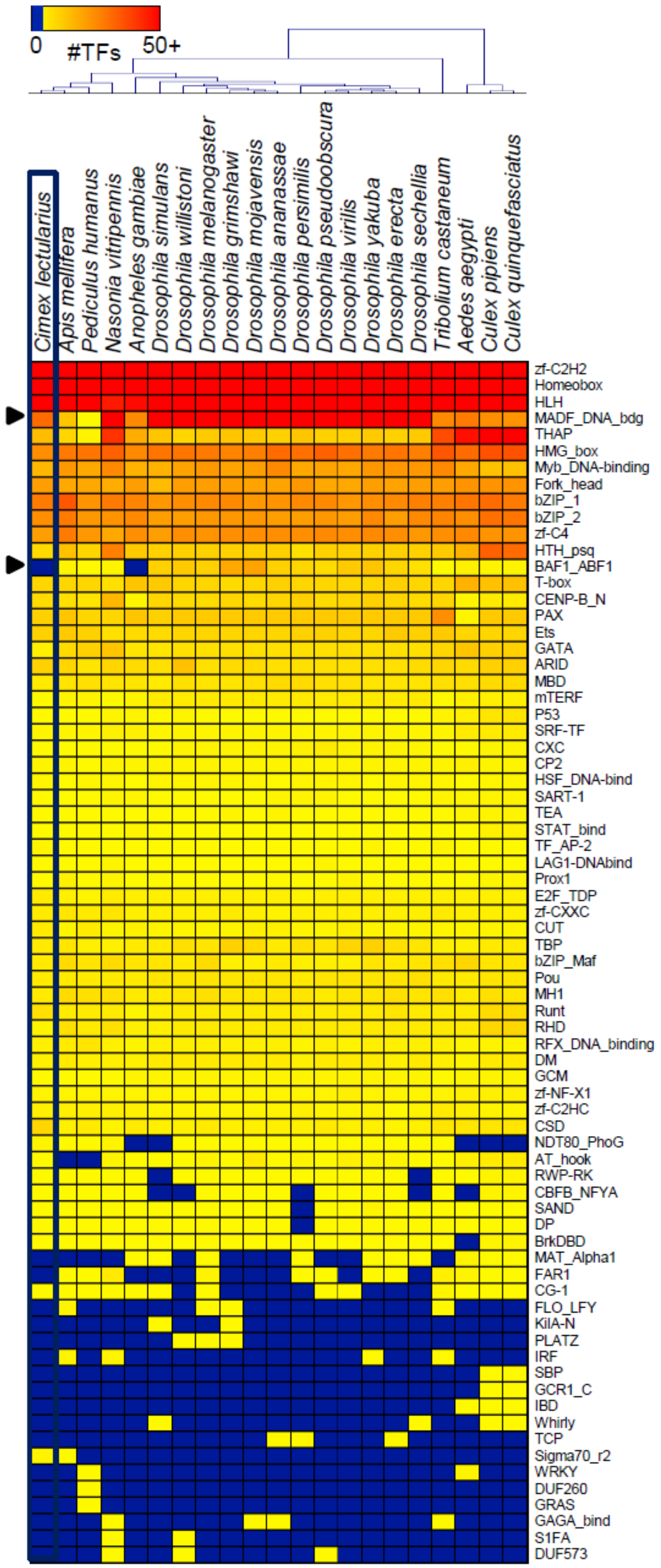

Supplementary Figure 41. Distribution of transcription factor families across insect genomes. Heatmap depicting the abundance of transcription factor (TF) families across a collection of insect genomes. Each entry indicates the number of TF genes for the given family in the given genome, based on presence of DNA binding domains. Color key is depicted at the top (blue means the TF family is completely absent). Species and TF families were hierarchically clustered using average linkage clustering. *C. lectularius* is boxed, and two TF families discussed in the text are indicated with triangles.

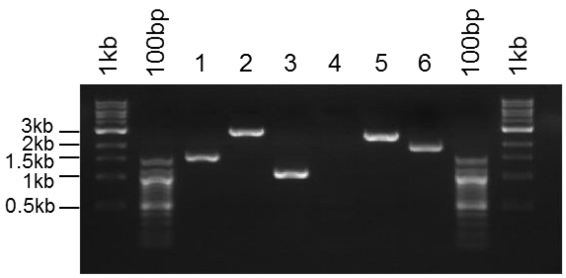

LGT region	Forward Primer	Reverse Primer	Predicted amplicon	Amplicon observed	Gel lane	Sequence confirmed?
Scaffold99:1354819..1357430	CAGTTAGATAGTTATTTCAAATGTGGAGG	CCTACTTAGGAGAAAGTGGCAGTTGAA	1.4kb	1.4kb	1	YES
Scaffold99:1655412..1655601	TAAATTTGAGTTGTGGACTGAGTAGACTTGG	CGCCGATTAGAATTTCTTCTGGTTGTTGG	2.9kb	2.9kb	2	YES
Scaffold36:1967220..1967393	CCTTATTCAGTGACACTTTCAACATCGCCAA	GCCGTCTTCTCCTAATAAGCCTCCTCCACC	1kb	1kb	3	YES
Scaffold45:578987..579332	GCCGTGACGCAAATGGGAAAGGAATC	CGCACAGGTGGCACAGTGAATGGCTGCG	2.5kb	-	4	No
Scaffold8:13305710..13306231	GTCTGTTTTGTGTGGGAAGACAGTGATTGC	CGACTGCCGATCAGTTACGACCACCAG	1.2kb	2.5kb	5	YES
Scaffold34:338260..338512	TGCACTGCAGAAATGCTGGATAATAG	CTACATTTGGAGAACTCCTCATTATAG	2kb	2kb	6	YES

Supplementary Figure 42. Confirmation of candidate Lateral Gene Transfers (LGTs). LGT regions were amplified using one of two procedures; either Phusion High-Fidelity DNA Polymerase (New England Biolabs; Ipswich, MA) or Phire Animal Tissue Direct PCR kit (Thermo-Fisher Scientific; Waltham, MA) and the indicated primers. Phusion PCR conditions were: 98°C, 30 sec; 98°C, 10 sec; 64.4°C, 30 sec; 72°C, 90 sec; 35 cycles; 72°C, 10 min using 100 ng of genomic DNA extracted from 10 Harlan strain *Cimex lectularius* females using the Nucleospin Tissue kit (Macherey-Nagel; Düren, Germany) as template. Phire PCR conditions were 98°C, 5 min; 98°C, 5 sec; 64.4°C, 5 sec; 72°C, 1 min; 40 cycles; 72°C, 1 min using the dilution protocol with legs of Harlan strain *Cimex lectularius* females. Amplicon sizes were observed by gel electrophoresis. Successful Amplicons were purified using the Nucleospin Gel and PCR Cleanup kit (Macherey-Nagel; Düren, Germany) and sequenced with the primers used for amplification.

These notes were provided to complement the manual annotation process. The sections have been edited minimally for formatting, but largely remain as is to convey information provided directly from each annotation group.

SUPPLEMENTARY NOTE 1

Community curation

A total of 1,352 gene models and 1,479 mRNA models were curated, in addition to 2 pseudogene models. Some models only received functional curation, which includes gene and mRNA names, symbols, descriptions, PubMed references, Gene Ontology categories, cross-references to other genes, and other comments explaining annotation actions as necessary; others were both structurally and functionally annotated. Curators handled a diversity of genes, although some gene families were dominant. For example, 146 cuticle proteins from the CPR family were curated, along with 102 unclassified cuticle proteins; 114 chemoreceptors were manually annotated, which includes 30 IRs, 36 GRs, and 48 ORs. Information about the *C. lectularius* genome project is available at its i5k Workspace@NAL organism page (https://i5k.nal.usda.gov/Cimex_lectularius). All tracks used by the general curation group, as well as the Official Gene Set, are publically accessible via the JBrowse genome browser[8] at the i5k Workspace@NAL (https://apollo.nal.usda.gov/cimlec/jbrowse/)[9]. The genome assembly and Official Gene Set can also be searched via BLAST+[10] (https://i5k.nal.usda.gov/webapp/blast/). Information on all manually curated genes is provided as a summary table (Supplementary Data 2).

SUPPLEMENTARY NOTE 2

Significance of antioxidant genes in bed bug biology

Blood meals are rich in pro-oxidants, and are known to contain high concentrations of compounds that lead to formation of reactive oxygen species (ROS). It is a well-known fact that digestion of hemoglobin, and specifically, heme, generates a plethora of ROS. A strong antioxidant enzyme system is therefore required to ameliorate and overcome blood meal-induced oxidative stress. We identified 36 genes belonging to 8 primary and secondary antioxidant gene families (excluding glutathione transferases) in the bed bug genome (Supplementary Data 3-5). Bed bugs possess all the antioxidant enzymes found in other blood-feeding insects like *Rhodnius, Pediculus*, and *Anopheles*. Interestingly, however, preliminary analysis shows that bed bugs have more catalase (Cat) and thioredoxin reductase (TrxR) genes than *A. gambiae, P. humanus and D. melanogaster*. Catalases are known to prevent the formation of free hydroxyl radicals by reducing hydrogen peroxide into water and oxygen and TrxRs are important for catalyzing the activation of the antioxidant enzyme, thioredoxin ($TrxS_2 \rightarrow TrxSH_2$). Previous research has shown that a strain of *Anopheles gambiae* refractory to *Plasmodium* infection exhibited differential expression of certain thioredoxin, catalase, and superoxide dismutase genes following blood feeding[11]. Analyzing expression of antioxidant genes in the bed bug before and after blood feeding would reveal significant genes associated with ROS detoxification, heme digestion, and immunity. Finally, three bacterial catalases were also annotated and could likely indicate contamination from endosymbiotic bacterial DNA as noted in *Apis mellifera* and *Drosophila* species genome sequencing projects[12,13].

SUPPLEMENTARY NOTE 3

Aquaporin genes in the bed bug genome

We have identified 7 aquaporin genes for bed bugs that include the typical Drip, AQP2, AQP4 (two sequences), AQP5, AQP6 and Bib (Supplementary Fig. 1). This number falls within the range of most insects (6-8) and *Cimex* has members of each group previously identified for insects[14]

SUPPLEMENTARY NOTE 4

<u>Supplementary text for the *Cimex lectularis* chemoreceptors</u>

The chemoreceptor (OR, GR, and IR) families were manually annotated. Briefly, TBLASTN searches of the genome assembly were performed using *Acyrthosiphon*, *Pediculus*, and *Drosophila* proteins as queries, and gene models were manually assembled in the text editor TEXTWRANGLER. Iterative searches were conducted with each new *Cimex* protein as query until no new genes were identified in each major subfamily or lineage. Additional searches included BLASTP and PSI-BLASTP searches[15] of both the MAKER and AUGUSTUS gene model proteins (the BLASTP searches of the AUGUSTUS proteins were most useful, with the subsequent PSI-BLASTP searches turning up only one additional divergent OR). All of the *Cimex* genes and encoded proteins are detailed in Supplementary Data 6-8. The gene models for these have been updated in the WebApollo genome browser.

Rather unusually there were no long pseudogenes in any of the three families, but there were several shorter fragments of genes that were not included in Supplementary Data 6-8 or the analyses because they encode less than 50% of a typical family member length. All *Cimex*, *Acyrthosiphon*, and *Pediculus* proteins in each family, as well as select other insect GRs, and all *Drosophila melanogaster* IRs, were aligned in CLUSTALX v2.0[16] using default settings, and problematic gene models were refined in light of these alignments. For the GRs, whole family alignments appeared unsatisfactory, so separate alignments of the sugar, carbon dioxide, fructose, and then remaining GRs by species were performed and then combined in profile alignment mode to obtain the final alignment. PhOr11 and 12 are too short to include and ApGr12 was removed as it is so highly divergent it disrupts the alignments.

For phylogenetic analysis, the poorly aligned and variable length N-terminal and C-terminal regions were excluded, as were any long internal length difference regions, for example between the longer ORCO proteins and most of the other ORs, and multiple regions within the IR alignment, using TRIMAL v4.1[17], with positions retained only if

present in 80% of the sequences. Phylogenetic analysis was carried out using maximum likelihood executed in PHYML v3.0[18] with default settings. Trees were colored and arranged in FIGTREE v1.4 (http://tree.bio.ed.ac.uk/software/figtree/), declaring roots as indicated in each figure legend.

All peptide sequences for the GRs, IRs, and ORs can be acquired by request from Hugh Robertson (hughrobe@uiuc.edu) or Joshua Benoit (joshua.benoit@uc.edu).

The odorant receptor family

The odorant receptor (Or) family of seven-transmembrane proteins in insects mediates most of insect olfaction (e.g. Su et al.[19], Touhara and Vosshall[20]), with additional contributions from a subset of the distantly related gustatory receptor (Gr) family, for example, the carbon dioxide receptors in flies[21-24], and a subset of the more recently described and unrelated ionotropic receptors (IRs)[25-28]. The Or family ranges in size from a low of 12 genes in the human body louse *Pediculus humanus*[29] to 400 in the ant *Pogonomyrmex barbatus*[30]. The other sequenced hemipteroid insect, the pea aphid *Acyrthosiphon pisum*, has 79 genes[31], which is an average size for insects. Although most of the 60 Or genes in *Drosophila melanogaster* are scattered around the genome (e.g. Robertson et al.[32]), with only a few in small tandem arrays, tandem arrays are more typical of other sequenced insects, especially those with large repertoires, from which it is inferred that these larger repertoires partly result from retention of gene duplicates generated in these tandem arrays by unequal crossing over (e.g. Robertson and Wanner[33]).

The ClOr gene set consists of 48 gene models, with one model encoding two proteins through alternative splicing, for a total of 49 proteins (Supplementary Data 6). All are intact, which is somewhat unusual for insect odorant receptors, where there are usually at least a few pseudogenes. Two genes are nevertheless incomplete because of gaps in the assembly, but they are likely to be intact in the genome. Two more required fixes of the assembly. ClOr12 has part of an exon missing in a gap and it was fixed with raw reads. More remarkably, the ORCO gene also required a fix to replace an in-frame stop codon in the third exon. The raw reads reveal that this is a “polymorphism” involving a 3-bp indel, which in the intact version introduces an extra amino acid as well (thus the

version in WebApollo is a readthrough of the pseudogenic allele). The exact nature of this situation is unclear, because approximately equal numbers of reads are present for each version in all libraries, including the long mate pair libraries that were generated from multiple individuals. It seems unlikely that a balanced polymorphism would have precisely 50% heterozygotes in the lab colony, unless it was a balanced lethal (presumably involving this gene and/or neighboring genes). Alternatively, both copies of this gene exist in the genome and their assembly was merged in the whole genome shotgun assembly. Detailed examination of the two haplotypes has not yet resolved this issue.

The MAKER set of gene models employed as the Official Gene Set was particularly depauperate for these Ors, with partial models for just three genes, however most of them were at least partially modeled in the AUGUSTUS set (Supplementary Data 6), with only 7 absent, although many required changes. The gene structures of these Ors share a few features with other insect Or genes, specifically the commonly present final three phase 0 introns and a preceding phase 2 intron, which was commonly preceded by a long first exon, however this long first exon was often interrupted by a variety of additional introns, up to a total of six in Or39 (Supplementary Data 6; Supplementary Fig. 2).

The phylogenetic tree reveals that the *Cimex* and *Pediculus* OR families contain mostly old lineages, with entirely species-specific expansions. In stark contrast, the *Acyrthosiphon* ORs consist of two large expansions where most genes are very young[31]. Most of the bed bug ORs have long branches, with only a few recent duplications, e.g. Or 7/8, 10/11, 13/14, 16/17, 18a/b, 25/26, 29/30, and 40/41, and most of these are tandem pairs in the genome (as are a few older pairs and one triplet – Supplementary Data 6). It appears therefore that the olfactory abilities of the bed bug and *Pediculus* have not changed much for a long time, while *Acyrthosiphon* has undergone enormous recent changes in its olfactory abilities[34].

The gustatory receptor family

The gustatory receptor (Gr) family of seven-transmembrane proteins in insects mediates most of insect gustation (e.g. Su et al.[19],Touhara and Vosshall[20], Vosshall and Hansson[35]), as well as some aspects of olfaction, for example, the carbon dioxide receptors in flies[21-24]. The Gr family ranges in size from a low of 6 genes encoding 8 proteins in the human body

louse[29] and 10 genes in the honey bee *Apis mellifera*[33] to 215 genes encoding 245 proteins in the flour beetle *Tribolium castaneum*[36]. The pea aphid *Acyrthosiphon pisum* has 77 Gr genes[36]. The Gr family is more ancient than the Or family, which was clearly derived from within it[32,37], and is found in the crustacean *Daphnia pulex*[38], the centipede *Strigamia maritima*[39], the tick *Ixodes scapularis* (HMR, unpublished), and many other animals (Saina et al.[40]; HMR, unpublished). This evolutionary history is reminiscent of the more recently described ionotropic receptors (Irs)[24,25,27], many of which also function in gustation[41,42].

The ClGr gene set consists of only 24 models, encoding 36 proteins, smaller than that of most other insects, except *Apis mellifera*[33], *Pediculus humanus*[29], *Ceratosolen solmsi*[43], and *Glossina morsitans*[2]. Like the Ors, there are no long pseudogenes, although a few highly degraded pseudogenic fragments are present in the genome. Five genes were modeled as being alternatively spliced, in the same fashion as several Grs in flies and some other insects, with alternative long first exons spliced into three shared short C-terminal exons, although in the absence of transcriptome evidence these models remain hypothetical. Some of these proteins are so divergent we were concerned about missing some, so in addition to TBLASTN searches, a final check for possible divergent genes/proteins was performed by PSI-BLASTP search of the AUGUSTUS modeled proteins with two iterations, which did not reveal any new models (the AUGUSTUS models more commonly included the existing Grs – see Supplementary Data 8). The AUGUSTUS modeling had access to all available insect Grs in GenBank, for comparative information, and succeeded in building at least partial gene models for 19 of these 24 genes (but not the alternatively spliced transcripts); however, only one of these was incorporated into the official gene set. Most of the AUGUSTUS models required at least one change. The basic gene structure for the entire ClGr set is a long first exon, followed by three short C-terminal exons separated by three phase 0 introns. The locations of these introns and their phases are the same as predicted by Robertson et al.[32] to be ancestral to the entire insect chemoreceptor superfamily, and are also shared with Gr genes in other animals (Saina et al.[40]; HMR unpublished). There were only a few exceptions: Gr5 has one additional intron while Gr1-4 has 2-3 additional introns, all interrupting the first long exon.

Cimex contains four genes encoding proteins related to the highly conserved

carbon dioxide receptors of flies, and these were named Gr1-4 (Supplementary Figure 3). This carbon dioxide lineage is absent from all Hymenoptera sequenced to date, as well as *Acyrthosiphon* and *Pediculus*, so they appeared to have been lost repeatedly. A large related subfamily expansion was discovered in the termite *Zootermopsis nevadensis*[44], indicating that this gene lineage is indeed ancient in insects, and this finding of them in *Cimex* confirms this inference. It remains to be shown that they indeed participate in perception of carbon dioxide.

Cimex also contains a gene encoding another conserved protein, named ClGr5, an ortholog of the DmGr43a protein that functions as a fructose receptor (Supplementary Fig. 3)[45]. Similar inferences have been made about the *Bombyx mori* Gr9 protein[46], so this entire lineage likely serves this role, with only *Pediculus* having lost it. There are, however, no obvious members of the sugar receptor subfamily (represented by AmGr1/2 and ApGr1-6 in Supplementary Fig. 3), a feature shared with other insects living strictly on vertebrate blood meals, including *Pediculus*[29] and tsetse flies[2].

The remaining *Cimex* GRs (6-24) are quite divergent from any of the conserved Grs, and form a distinct lineage in the tree. These include all of the alternatively-spliced models. As was true for most of the Ors, the long branches to most of these proteins are similar to those to the *Pediculus* proteins, and in stark contrast to most of the aphid Grs, which form two recently expanded gene subfamilies that reveal evidence of positive selection of amino acids indicative of adaptive divergence[31]. Most of the remaining *Drosophila* Grs are implicated in perception of bitter tastants, but it is hard to be confident of such a function for these bed bug Grs and their *Pediculus* relatives.

The ionotropic receptor family

In addition to the Or and Gr families in the insect chemoreceptor superfamily[32], there is a second completely different family of olfactory and gustatory receptors in insects, the ionotropic receptors[25,28], which clearly evolved from the ionotropic glutamate receptors involved in synaptic transmission[26]. These proteins are somewhat larger than the Ors and Grs, and have three transmembrane domains comprising a cation channel and an external ligand-binding domain. They function as obligate heterodimers or higher multimers. While some of these Irs are highly conserved, and have been implicated in olfaction, others are

highly divergent and some are implicated in gustation[28,41,42]. Like the Ors, all of which function as heterodimers with the highly conserved ORCO protein[40], most IRs function in complexes with some of the most conserved proteins, specifically IR8a and/or IR25a[27,28].

The ClIr gene set consists of 30 models, larger than *Acyrthosiphon* with 19 and *Pediculus* with 14 (Supplementary Fig. 4)[26,44]. This number is nevertheless considerably less than the 65 genes in *Drosophila melanogaster*[23,25,28], which has at least one large fly-specific expansion, and a lot smaller than the termite *Zootermopsis nevadensis*, which has 150 Irs[44]. Once again there are no large intact pseudogenes, although the N-terminus could not be identified for two genes (Ir41e and 75b), so they might be pseudogenes but these might also be genome assembly problems, while Ir41d contains a gap in the assembly that was repaired with raw reads (Supplementary Data 7). The AUGUSTUS modeling succeeded in building at least partial gene models for all 30 genes, and 14 of these were incorporated into the official gene set, although all 14 required modifications. Gene structures for the Irs vary enormously, from a typical number of 7 or 8 introns (although Ir101-103 have lost all their introns, presumably due to a recombination event with a cDNA copy in their common ancestor), to 18 in Ir93a (Supplementary Data 7).

Naming of the Irs is somewhat complicated. Following the example of Croset et al.[26], those with obvious simple orthologs in *Drosophila* were named for that gene/protein, despite these names having no significance for the bed bug, having been designated for their cytological location in *Drosophila melanogaster* (see also Terrapon et al.[44]). *Cimex* has two paralogous amplifications of receptors that are also multi-copy in *Drosophila* (DmIr41a/76a/92a and DmIr75a-d/31a/64a/84a), and these were named with lower case letters that do not imply orthology with the similarly named *Drosophila* genes (Ir41a and Ir75a-d). Finally, *Cimex*, like *Pediculus* and *Acyrthosiphon*, has a set of highly divergent IRs only weakly related to the divergent Irs of *Drosophila*, and these were named Ir101-106 to avoid any confusion with the *Drosophila* Irs, which only go up to Ir100a.

The ligand-specificity is known for only a few Irs in *Drosophila*, so relatively little can be said about possible ligands and roles for these *Cimex* Irs. Grosjean et al.[47] report that DmIR84a along with Ir8a is responsible for perception of phenylacetic acid and phenylacetaldehyde, but Ir84a has no simple hemipteran ortholog, albeit being part of the Ir75 expansions (Supplementary Fig. 4). In *Drosophila*, Ir75a-c along with Ir8a are

implicated in perception of propionic acid, while Ir76a (which is related to the Ir41a expansion), Ir76b (a reasonably conserved potential co-receptor), and the co-receptor Ir25a form a functional receptor for phenylethyl amine. Thus it is possible that the *Cimex* relatives of some of these lineages are involved in similar perception. The large expansion of the IR75 lineage into 12 genes is of particular interest and might be important in blood feeding. This lineage is, however, shrunk to two genes in *Pediculus*, which is also an obligate blood feeder, and also separately expanded to 17 genes in the termite *Zootermopsis nevadensis*[44], where they are presumably involved in some other aspect of chemical ecology.

SUPPLEMENTARY NOTE 5

Circadian clock genes in the bed bug genome

Circadian clocks have evolved to allow organisms to synchronize their metabolism, physiology, and behavior with the external environment. While the molecular mechanisms through which the molecular clocks of different organisms work are very well conserved, the clock proteins that compose them are quite varied. In Diptera the key players of the first feedback loop of the circadian clock are PER (PERIOD), TIM (TIMELESS), CLK (CLOCK) and CYC (CYCLE) (reviewed by Peschel and Helfrich-Förster[48]). In Hymenoptera TIM is not present at all and PER is known to heterodimerize with CRY2 (reviewed by Bloch[49]). CRY2 is homologous to the mammalian CRY: it does not function as photoreceptor but as transcriptional repressor[50,51]. This role is exploited in Diptera by PER[52,53]. If we further look at Lepidoptera we can find that both CRY (mammalian-like and *Drosophila*-like) are present, with CRY1 being photosensitive and CRY2 acting with TIM and PER as transcriptional regulators (reviewed by Reppert[54]).

In *C. lectularius* the first feedback loop of the clock seems to be relying on CRY2 (mammalian-like), PER and TIM (both *Drosophila*-like) (Supplementary Data 9). In all three proteins there are conserved domains compared to either the human or *Drosophila* homologs (Supplementary Fig. 5). TIM seems to be the most conserved protein, at least compared to its homolog in *Drosophila* (Supplementary Fig. 5). In *C. lectularius* TIM (ClecTIM) we could not identify a cytoplasmic localization domain (CLD). Nevertheless it was shown by Ousley et al*.*[55] that this particular sequence is also the least conserved among different *Drosophila* species. ClecPER presents differences from both the human and *Drosophila* proteins (Supplementary Fig. 5). We could not map the nuclear localization sequence (NLS) as well as the first PAS domain. Moreover, ClecPER has a Period C domain, necessary for binding CRY[56]. On the other hand, ClecCRY does not present the PER2 binding domain (typical of mammalian-like CRYs). Moreover, ClecCRY does not have a C-terminal tail (CTT domain) that has been shown to be essential for TIM/CRY interaction in *Drosophila*[57].

In the *Cimex* genome we did not find any sequences for either CRY1 (*Drosophila*-like) or JET (JETLAG) (Supplementary Data 9) both necessary in *D. melanogaster* for the light input pathway to the clock[58,59]. It is possible that in *C. lectularius* TIM acts simply by increasing PER stability or that in this molecular clock CRY2 acts as a blue light photoreceptor. It is known indeed that also mammalian-like Cryptochromes can be activated by light in living cells[60]. Whether *C. lectularius* CRY acts indeed as a photoreceptor or acts by repressing the activity of the two transcription factors CLK and CYC remains to be determined experimentally.

SUPPLEMENTARY NOTE 6

Cuticular proteins

It is well established that the bed bug cuticle plays a substantial role in resistance to insecticides; this is thought to be due (at least in part) to changes in the expression of bed bug cuticle proteins in resistant strains[61-64]. Identification and classification of bed bug cuticle protein genes is essential to our understanding of the genetic and physiological basis for penetration-based insecticide resistance. Using the criteria established by Willis[65], we identified 273 genes that encode putative cuticle proteins. Of these, 169 genes could be placed in one of 8 families (CPR, CRPL, CPF, CPFL, CPAP1, CPAP3, TWD, Dumpy), with an additional 104 proteins consisting of repeated low complexity sequences (AAPV/GGY) commonly associated with cuticle proteins but without a defining conserved domain (Supplementary Data 10). Of these, 195 (71.4%) were arranged in 29 clusters of 3 or more genes; altogether these clusters spanned approximately 5.8 Mb (Supplementary Data 11; Supplementary Figure 6a,b). Six clusters contained 12 or more genes; these clusters spanned 130-920 Kb each. Clusters were largely type-specific, with low complexity proteins, CPRLs, and CPR proteins containing the RR-1 or RR-2 type chitin-binding domain (CB4; pfam 000379) occupying separate clusters. However, there were three occasions where proteins from separate families were co-located in the same cluster: cluster 1 (CPR/CPRL); cluster 5 (CPF/CPFL/CPAP1); and cluster 9 (CPR/CPAP3). The fact that bed bug cuticle proteins (as in other insects) are arranged in gene clusters may accelerate the development of insecticide resistance, as genes within a cluster may be coordinately regulated; thus one regulatory change could affect the expression of many or all genes in the cluster. Alternatively, gene clusters are prone to expansion via unequal crossing over, which can be facilitated by the highly identical nature of the genes in the cluster.

As in other insects, the CPR family represented the largest single family of putative cuticle protein genes found in the bed bug genome, and these separated relatively neatly between RR-1 (soft cuticle) and RR-2 (hard cuticle) types, the latter of

which are far more abundant in the genome (Supplementary Fig 7). The 121 CPR-type genes we identified is slightly more than *Drosophila*[66] but less than the silkworm[67] or the malaria mosquito[68,69]; data for other hemipterans is not currently available. While the number of genes is not extraordinary, we note several interesting features of bed bug CPR genes. Virtually all CPR genes contain only a single CB4 domain, though each insect genome examined to date seems to contain a few exceptions. For the bed bug, these would be CPR115 (6 CB4 domains), CPR116 (2 CB4), and CPR14 (2 CB4), as well as an interesting cluster of 10 genes located on Scaffold 24. These genes all have an identical gene structure consisting of a signal peptide encoded by the first exon, and a CB4 domain encoded by each of the next two exons (Supplementary Fig. 7). Examination of the coding sequence suggests that these genes arose from an interesting duplication of CPR45 (located in the same cluster) to generate both donor and acceptor splice sites derived from different pre-existing parts of the ancestral gene. This event must have occurred relatively recently, as this cluster is not present in the other hemipteran genomes (*R. prolixus* and *A. pisum*), though homologs of the ancestral CPR45 gene are.

Adelman and colleagues previously identified the bed bug pro-resilin gene, a conserved CPR (now CPR78) containing an RR-2-like CB4 domain, an N-terminal consensus (EPPVNSYLPPKS) and a series of glycine-rich repeats[61]. Upon analysis of the full genome sequence, we identified four other CPR genes that cluster with CPR78 that each contain >20% glycine. Interestingly, in a second cluster of CPRs 5 out of 8 genes also contain >20% glycine, with CPR22 at 31%. What was most surprising was the identification of CPR57 and CPR58, both G-rich CPRs with a clear RR-1 consensus CB4 domain, but also a clear pro-resilin consensus at the N-terminus (Supplementary Fig. 8). In fact CPR57 is a protein of over 600 amino acids and is more than 40% glycine. This suggests that bed bugs may have expanded and diversified the resilin family, potentially to accommodate the stretching and reformation of the cuticle required during the acquisition of a blood meal.

Other cuticle protein families, such as Twdl, CPF, CPAP1 and CPAP3, are well-conserved between bed bugs and other insects; a slight expansion the alanine-rich CPF family (25-30% Ala) was observed, as bed bugs encode 5 such genes compared to *R. prolixus* (2) and *A. pisum* (2) (Supplementary Fig. 9,10). Finally, we identified a cluster of

17 bed bug genes that encode predicted proteins containing between 1-12 copies of the 18-amino acid motif identified by Nakato et al.[70] and Anderson et al.[71] from insect cuticles (Supplementary Fig. 11). As these genes are located in a cluster with CPR type genes, and share similar low-complexity regions with these same genes (as well as the 18-amino acid motif), we propose to name this family CPR-like, or CPRL. The defining features for this family would thus be the presence of 1 or more 18-amino acid motifs and the absence of a CB4 chitin-binding domain.

SUPPLEMENTARY NOTE 7

The repertoire of digestive genes in *Cimex lectularius*

A total of 10 gene groups were annotated that are potentially associated with digestion including serine proteases, cysteine proteases, aspartate proteases, carboxypeptidases, aminopeptidases, and lipases. Most of these putative proteins were characterized with secretory signal peptides in the N-termini, suggesting that they are secreted into midgut lumen and thereby participating in the breakdown of dietary proteins and lipids, which are important nutrient components of a blood meal. Compared to the number of other proteases within the *Cimex lectularius* genome, we observed serine proteases as the largest gene class consisting of 87 gene members in total (Supplementary Data 12). Although this is not as large as the numbers of serine proteases present in dipteran species, e.g. *Drosophila melanogaster* (204 gene copies) and *Anopheles gambiae* (305 gene copies), and coleopteran species, e.g. *Tribolium castaneum* (~160 gene copies), the number of serine proteases identified in *C. lectularius* seems to be the most abundant digestive enzymes. Furthermore, the repertoire of serine proteases within the *C. lectularius* genome is in tandem distribution on DNA scaffolds suggesting a linear expansion of these genes during evolution. Specifically, 13 serine protease genes, most of which contained 6-9 exons, are located in tandem within a 323-kb region on Scaffold 51. Strikingly, we further revealed a total of 32 serine protease genes that contain only a single exon and 22 of which clustered as a single subclade in our phylogenetic analysis (Supplementary Fig. 12). Most serine protease genes in hemipteran insects consist of multiple exons, for example, only 4 out of 90 serine protease genes in *Nilaparvata lugens* contain single exons[72]. Therefore, the abundant presence of single-exon serine protease genes in the *C. lectularius* genome and their phylogenetic relatedness indicate that they were recently expanded through gene duplication and/or suggest the rapid deployment of these genes during digestion. This expansion of the serine protease class of genes could

be attributed to the high demand of protein digestion after *C. lectularius* takes a (huge) blood meal.

In contrast to most other insects that have more cathepsin B and L (both are cysteine proteases) than cathepsin D (aspartic protease) genes, *C. lectularius* possesses more of the cathepsin D within its genome. For example, *Acyrthosiphon pisum* has 29 cathepsin B and 2 cathepsin L but only 1 cathepsin D while *C. lectularius* has 19 cathepsin D genes compared to 8 cathepsin B and 9 cathepsin L genes (Supplementary Data 12). However, this resembles another hemipteran blood-sucking insect, *Rhodnius prolixus*, whose digestive tract expressed 17 cathepsin D genes but only 2 cathepsin B and 6 cathepsin L genes[73]. Phylogenetic analysis suggests that cathepsin D genes probably expanded in *C. lectularius* multiple times during evolution independently from what occurred in *Rhodnius prolixus* (Supplementary Fig. 13). Cathepsin D is an aspartic protease that favors acidic pH values, a feature found in hemipteran insects, for optimal activity[74]. We observed, however, an unusually high number of Cathepsin D genes present in the *C. lectularius* genome. If these genes are expressed in *C. lectularius* midgut, similar as those found in *R. prolixus*, their duplication in the genome is likely an adaption to the digestion of a large blood meal.

SUPPLEMENTARY NOTE 8

<u>Comparison of epigenetic systems in *Oncopeltus* and *Cimex*</u>

It is not clear if *Oncopeltus*, *Cimex,* or *Rhodnius* have functional DNA methylation systems. An ortholog of Dnmt3 (the de novo methyltransferase) was not identified in *Oncopeltus*, *Cimex,* or in *Rhodnius prolixus*. This is suggestive of a loss of Dnmt3 in the lineage leading to this clade of insects. However, the *Oncopeltus*, *Cimex,* and *Rhodnius* genomes do encode copies of the maintenance methyltransferases (two copies in *Oncopeltus*, one in *Cimex*, two in *Rhodnius*). All three genomes encode an ortholog of Tet1 (putative demethylation enzyme).

Oncopeltus is unusual in that there is a very small number of genes encoding histone proteins and no loci could be detected that encode the linker histone, Histone H1. This seems specific to *Oncopeltus* as *Cimex* has a large number of loci encoding histone proteins, similar to *Daphnia* (Supplementary Data 13).

In *Drosophila* the histone genes are present in the genome in large numbers of quintet clusters, each cluster having one gene from each of the five classes of histones. This arrangement of genes is also observed in other insects such as the pea aphid[75]. In *Cimex* we see two-quintet cluster of histone genes (Supplementary Fig. 14). The remainder of the histone genes is only present as single copies on a scaffold, are interrupted by non-histone encoding genes, or are the result of recent gene duplications. *Oncopeltus,* unlike *Cimex*, does not have these quintet clusters. All of the histone genes are present as single copies on a scaffold.

Oncopeltus is unusual in that there are duplications of the MYST histone acetyltransferases *mof* (males absent on the first) and *enok* (enoki mushroom). Duplications of these genes have only previously been reported for *Acyrthosiphon pisum*[74]. *Cimex* also has duplications of these genes (*enok* and *mof*), but *Rhodnius* does

not. Phylogenetic analysis indicates that these genes have duplicated independently in the lineages leading to *Cimex* and *Oncopeltus* from the lineage leading to the pea aphid (Supplementary Fig. 15).

SUPPLEMENTARY NOTE 9

Visual genes / Light detection

Summary

Bed bugs are equipped with relatively small but canonically organized compound eyes that protrude prominently from the lateral head capsule[76,77]. In behavioral assays, beg bugs are attracted to darker objects and there is tentative evidence of object recognition, suspected to play a role in host habitat detection[78]. Consistent with this evidence for low resolution landscape vision, the bed bug genome contains a relatively small set of known light-sensitive G-protein coupled transmembrane receptor genes including one member each of the UV- and broadband long wavelength-sensitive rhabdomeric opsin subfamilies. This is in line with most other hemipteran genomes sequenced so far (Supplementary Fig. 16) but also with crepuscular insect species in general[89].

Annotation

The UV-opsin subfamily is conserved in Holometabola. UV-opsin(s) have been recovered in all Hemiptera, including *Cimex*, which has a singleton ortholog 2.

The B-opsin subfamily is a visual opsin that is conserved in Holometabola. This subfamily is missing in *Cimex* and was likewise not recovered in most Hemiptera (*Acyrthosiphon pisum, Cimex lectularius, Drosophila melanogaster, Oncopeltus fasciatus, Rhodnius prolixus*). However, singleton B-opsins were found in *Pachypsylla venusta* and *Frankliniella occidentalis*, so this loss likely happened at some point during hemipteran evolution.

The LW-opsin subfamily is visual opsin that is conserved in Holometabola. All hemiptera have a gene(s), including *Cimex*, which has a singleton ortholog.

The C-opsin subfamily is a non-retinal opsin that is conserved in many holometabola but not in Diptera. Most Hemiptera have C-opsin gene(s), including *Cimex,* which has a singleton ortholog.

The Rh7-opsin subfamily is conserved in holometabolous insects by has not been functionally characterized. All Hemiptera, including *Cimex*, have a singleton ortholog.

The arthropsin subfamily was recently discovered in *Daphnia* and other non-insect arthropods but has not been functionally characterized. Partial sequences for arthropsin genes were discovered in *Oncopeltus* and the pea aphid, but no genes were recovered from *Cimex.*

With only two visual opsins conserved, the *Cimex* opsin repertoire seems typical of that of highly crepuscular species such as the human louse *Pediculus humanus* or the red flour beetle *Tribolium castaneum*.

SUPPLEMENTARY NOTE 10

Autophagy Genes

The following autophagy genes were annotated: IAP1, VPS34, Atg6, Atg14, Fk506-bp1, Atg4, Atg3_Aut1, Atg9, Atg2, Atg13, Atg1 (named ULK2), and PSMDS (Supplementary Data 14). Atg8a and Atg8b both hit only one model indicating only a single Atg8 gene within *C*. *lectularius*. Overall, the autophagy gene group is conserved in comparison to other insect systems[79].

SUPPLEMENTARY NOTE 11

Heat shock Genes

Bed bug heat shock genes are similar to genes that encode heat shock proteins in other insects (Supplementary Data 15). Several match to the same gene model which could represent a reduction or lack of duplication events in bed bugs compared to other insects[80].

SUPPLEMENTARY NOTE 12

Hox cluster

Hox genes are a classic example of conservation across the Bilateria, both for the genomic organization and the developmental function of these transcription factors [81,82]. We were able to find all ten Hox genes (Supplementary Data 16) in the expected order and orientation (same transcriptional orientation for all genes, with the anteriormost gene at the 3' end of the cluster) on one of the largest scaffolds of the *Cimex* genome (Scaffold 8, 16.6 Mb: Supplementary Fig.17A). The cluster occupies a 3.5 Mb region. The difference in Hox cluster size seems to be proportional to the genome size compared to the coleopteran *Tribolium castaneum* (160 Mb genome with a 0.71 Mb cluster[83]) and the dipteran *Drosophila melanogaster* (120 Mb genome with a split cluster combined size of 0.65 Mb[84,85]). This increase in size is largely due to an increase in intergenic and intronic distances. At the same time, the previously observed trend in protein size is perpetuated: just as *Tribolium* Hox proteins are smaller than their *Drosophila* orthologues[82], several of the *Cimex* orthologues show up to 20% protein size reduction compared to *Tribolium* (Lab, Pb, Dfd, Ftz), while the diverged Hox gene *zen* [86,87] is the only one that encodes a larger protein (25% larger than in *Tribolium*). Compared to *Tribolium*, splice sites are also well conserved for most genes, although *zen*, and the other diverged Hox gene, *ftz*, is again the exception.

Iro-C cluster

Synteny is also conserved within the small Iroquois-Complex (Iro-C), a second family of homeodomain transcription factors that arose from ancient tandem duplications. Whereas

Drosophila has three family members[88-90], we find that, as in *Tribolium, Cimex* possesses just two: *mirror* (*mirr*), conserved across the Insecta, and *iroquois* (*iro*), which is the single gene ortholog corresponding to the tandem paralogs *araucan* (*ara*) and *caupolican* (*caup*) in *Drosophila*. As in *Tribolium*, the two Iro-C genes in *Cimex* occur in tandem with the same transcriptional orientation along the scaffold, with *iro* upstream of *mirr* (Supplementary Fig. 17B), although the *Cimex* Iro-C cluster is 2.3- to 3-fold larger than in *Tribolium* and *Drosophila*, respectively. Both Iro-C genes are also fairly well conserved compared to *Tribolium* at the level of gene structure (3-4 conserved splice sites out of 4-6) and protein sequence (≥58% identity for ≥69% sequence coverage)

Assembly quality/accuracy of automated annotation

There were Maker predictions available for all the genes annotated here. However, the maker predictions were fragmented and it was necessary to manually inspect and merge at least two Maker models to build complete models matching the protein queries. The reader should be aware of the possibility of fragmentation when looking at other automated gene models. Good to very good RNA-seq evidence was available for all gene models, which greatly facilitated the prediction of exon structure and UTR assignment. Only one possible misassembly issue was spotted: in the model for *Abd-B*. This model has three exons in *Cimex*, in contrast to *Tribolium* and other insects where *Abd*-B has only two exons. The intron between exons 1 and 2 is largely filled by two big gaps, and the end of exon 1 and beginning of exon2 show the exact same nucleotide sequence. A duplication in this region was not observed in homology alignments, and we also found no way to set splice boundaries so as to keep only one copy of the sequence. Therefore, we suggest that the duplication is a misassembly artifact, and that the gene model should only consist of two exons.

Supplement: methodology

We annotated the Hox genes by performing tblastn searches on the *Cimex lectularius* scaffolds with the corresponding *Tribolium* and *Oncopeltus* Hox gene protein sequences available in NCBI (the current official gene set, OGS, models). To confirm orthology, we then blasted our *Cimex* models back into NCBI. Homology, intron/exon boundary

assessments, and protein sequence completeness were identified by manual inspection and correction of protein alignments generated with ClustalW2 (http://www.ebi.ac.uk/Tools/msa/clustalw2/).

Splice site information for *Tribolium* was obtained for the OGS gene models as accessed from Assemblies 3.0 and 4.0 in the genome browser of the Stanke group, University of Greifswald (http://bioinf.637 uni-greifswald.de/gb2/gbrowse/tcas4/).

Possible gene loci duplications were determined by performing tblastn searches on the scaffolds using the protein sequences of completed annotation models as queries, and then re-blasting the resulting hit sequences into NCBI for Arthropoda hits.

SUPPLEMENTARY NOTE 13

Insecticide targets

Our task was to identify and annotate the following insecticide target relevant genes and gene families:

1. Acetylcholinesterase
2. Ryanodine receptor
3. Chitin synthase
4. Ecdysone receptor (USP)
5. Octopamine receptor
6. Voltage-gated sodium channel
7. GABA-gated chloride channel
8. Glutamate-gated chloride channel
9. Other Cys-loop receptors (5HT, nAChR, GlyR, ZAC)

Data

BCM supplies the following data:

- Genome assembly
- Female and male RNA-seq
- MAKER gene models

WebApollo was used as a common annotation platform. Via WebApollo further data were supplied, e.g. blast mappings against Annelida, Arthropoda, Atelocerata, Cephalochordata, Chelicerata, Cnidaria, Craniata, Crustacea, Echinodermata, Mollusca, Nemata, Nematomorpha, Onychophora, Parazoa, Placozoa, Platyhelminthes, Priapulida, Tardigrada, Tunicata. Mappings of RNA-seq data were also supplied.

Method of tree building

In order to compare annotated CIMLE sequences to other species, they were blasted versus Subsets (*Tetranychus urticae, Acyrthosiphon pisum, Anopheles gambiae, Rhodnius prolixus, Pediculus humanus subsp. corporis, Apis mellifera, Tribolium castaneum, Bombyx mori*) from the Uniprot-Database as of June 2, 2014 using the following parameters:-p blastp –e 1e-3.

The best Blast hits from the chosen model-organisms were aligned with the CIMLE sequence using ClustalW. The settings were: cost matrix BLOSUM62, gap open cost 10, gap extend cost 0.1.

Phyml was used to build phylogenetic trees with the following settings: substitution model: JTT, tree build method: maximum likelihood, outgroup *Tetranychus urticae,* resampling method: bootstrap 100 replicates.

Acetylcholinesterase

Scaffold61: 760151..769073 reverse

Genomic DNA: 8923 bp

CDS: 1932 bp

Protein: 643 aa

Exons: 6

Supplementary Figure 18

Ryanodine receptor

Scaffold35: 4578680.. 4641356 reverse

Genomic DNA: 62681 bp

CDS: 15183 bp

Protein: 5060 aa

Exons: 90

Supplementary Figure 19

The change over from Exon13 to Intron started with a gc not with a gt. The RNA.seq data confirm this.

Chitin synthase:

Scaffold35: 1986702..2010750 reverse

Genomic DNA: 24049 bp

CDS: 4881 bp

Protein: 1626 aa

Exons: 22

Supplementary Figure 20

Comparison with *Rhodnius prolixus* chitin synthase and particularly the alignment of RNA-seq reads from females suggest a possible alternative splicing of exons 18 and 19 which could be either split or linked.

Ecdysone receptor / Ultraspiracle protein

Scaffold10: 11301169..11302993 reverse

Genomic DNA: 1825 bp

CDS: 1134 bp

Protein: 377 aa

Exons: 6

Supplementary Figure 21

Ultrapiracle protein

Scaffold28: 2466298..2469883

Genomic DNA: 3586 bp

CDS: 1134 bp

Protein: 377 aa

Exons: 7

Supplementary Figure 22

Octopamine receptor

Scaffold1: 33258221..33262539 reverse

Genomic DNA: 4317 bp

CDS: 1119 bp

Protein: 372 aa

Exons: 2

Supplementary Figure 23

Voltage-gated sodium channel

Scaffold2: 18435119..18463718

Genomic DNA: 28600 bp

CDS: 6039 bp

Protein: 2012 aa

Exons: 32

Supplementary Figure 24

GABA-gated chloride channel

The discrimination of alpha and beta subunits of the GABA-gated chloride channel is ambiguous. Both have blast hits at the same regions of scaffold 1 and scaffold 65 which are only partially covered by few RNA-seq read alignments and which are on both strands spanning more than 77 kb.

Scaffold1: 1845843..1872325 reverse

Genomic DNA: 26468 bp

CDS: 1527 bp

Protein: 508 aa

Supplementary Figure 25

Glutamate-gated chloride channel

Scaffold9: 6766328..6772105

Genomic DNA: 5778 bp

CDS: 1110 bp

Protein: 369 aa

Exons: 7

Supplementary Figure 26

Other Cys-loop receptors (5HT, nAChR, GlyR, ZAC)

5HT3-receptor (5-hydroxytryptamine / serotonin receptor)

The best blast hits are in the same region of Scaffold76 as the putative nicotinic acetylcholine receptor subunit beta.

Nicotinic acetylcholine receptor

Nicotinic acetylcholine receptor subunit alpha 2

Scaffold17: 6545425..6555469 reverse

Genomic DNA: 10045 bp

CDS: 1353 bp

Protein: 450 aa

Exons: 5

Supplementary Figure 27

Nicotinic acetylcholine receptor subunit alpha 3

Scaffold8: 14078389..14086394

Genomic DNA: 8006 bp

CDS: 1200 bp

Protein: 399 aa

Exons: 6

Supplementary Figure 28

The putative nAChR alpha 3 subunit is hardly backed by RNA-seq reads. The annotated transcript seems to be 5' incomplete since a start with GTC (V) is rather unlikely. There is no ATG in the same frame until the next stop codon upstream.

Nicotinic acetylcholine receptor subunit alpha 4

Scaffold16: 3374428..3379863
Genomic DNA: 5436 bp
CDS: 765 bp
Protein: 254 aa
Exons: 6
Supplementary Figure 29

Nicotinic acetylcholine receptor subunit alpha 6

Scaffold87: 1260375..1265223 reverse
Genomic DNA: 4849 bp
CDS: 504 bp
Protein: 167 aa
Exons: 3
Supplementary Figure 30

Nicotinic acetylcholine receptor subunit alpha 7

Scaffold19: 4569611..4580023 reverse
Genomic DNA: 10421 bp
CDS: 675 bp
Protein: 225 aa

Exons: 4

Supplementary Figure 31

<u>Nicotinic acetylcholine receptor subunit beta</u>

Scaffold76: 1381017..1388950

Genomic DNA: 7938 bp

CDS: 1581 bp

Protein: 526 aa

Exons: 9

Supplementary Figure 32

<u>Glycine receptor</u>

Scaffold3: 6894919..6900492

Genomic DNA: 5574 bp

CDS: 1122 bp

Protein: 373 aa

Exons: 9

Supplementary Figure 33

ZAC (zinc activated ion channel)

No blast hits were obtained using ZACN_CANFA,ZACN_HUMAN,T0MFV4_9CETA, S7QCT4_MYOBR, L9KZA2_TUPCH, L5MDE8_YODS, L8HVW9_9CETA, L5KNC4_PTEAL, G5AQV7_HETGA as query sequences.

SUPPLEMENTARY NOTE 14

Peptidergic and aminergic signalling in the bed bug *Cimex lectularius*

Most developmental and physiological processes are hormonally regulated or orchestrated by regulatory peptides or biogenic amines which are produced by endocrine or neuroendocrine cells. Neuropeptides and biogenic amines also act as neuromodulators or neurotransmitters within the nervous system, and play a key role in controlling behavior. Of special interest for hematophageous insects such as the bed bug are peptides and biogenic amines that induce or terminate post-feeding diuresis, as well as peptidergic and aminergic signaling networks that control feeding behavior and digestion[91]. While biogenic amines are metabolites, bioactive peptides are produced by posttranslational processing of larger precursor molecules called prepropeptides[92]. Most regulatory peptide and amine signals are received and transduced by G protein-coupled receptors (GPCRs), also known as seven transmembrane domain receptors. Peptides, biogenic amines and especially their GPCRs represent attractive molecular targets for synthetic or naturally occurring insecticides[93,94].

Prepropeptide genes

In the bed bug genome, we identified 50 genes encoding putative prepropeptides (Supplementary Data 17) containing >100 putative bioactive peptides that were predicted based on sequence homology to biochemically confirmed or predicted peptides from other insect species and the presence of flanking prohormone convertase cleavage sites[95]. *C.*

lectularius possesses the core set of 20 regulatory peptides common to all insects characterized thus far (Supplementary Fig. 34). The other 30 prepropeptide genes cover most insect peptides that occur only in a subset of taxa, including the recently identified CNMamide, RYamides, elevenin, natalisin, EFLamide and the restrictively distributed ACP (Supplementary Fig. 35). Like the brown planthopper (*Nilaparvata lugens*), the hitherto best characterized hemipteran in terms of peptides, *C. lectularius* also lacks trissin (found in holometabolous insects and the body louse[96], as well as neuropeptide-like precursor 2 (NPLP2) which is lacking in most insect species[97]. NPLP3 and NPLP4 homologs were annotated as neuropeptide precursors in the *Tribolium* genome, but rather represent cuticular peptides/proteins. We were unable to find a gene for inotocin/arginine-vasopressin-like peptide, which is, however, present in the *Nilaparvata* transcriptome[98]. *C. lectularius* appears to have only two insulin-like peptide (ILP) genes: one ILP B ("insulin-like") and one with some similarities to both ILP B and ILP C ("IGF-like"). Other Hemiptera have several ILP Bs[98,99]. Unlike *Rhodnius*[100,101], the bed bug appears to have also only one *capa* gene encoding anti-diuretic hormones (periviscerokinins). Remarkably, *C. lectularius* seems to possess an unusual myosuppressin (MS) sequence, which is longer and even more derived from the insect consensus than the already unusual MS sequence found in *Rhodnius*[102,103]. It is noteworthy that a derived MS sequence is not a general feature of the Heteroptera, as pentatomid MS shows an insect consensus sequence[104]. Also unusual is the methionine residue in position 2 of the bedbug Arg^7-corazonin. While Arg^7-corazonin is common among insects[105] and was also found in the reduviid bug *Triatoma infestans*[106], Met^2 is a new variant. These predicted sequences, however, will need biochemical confirmation.

From a genomic perspective, it is interesting to note that the loci of several highly related peptide genes that are thought to have arisen from gene duplications lie very closely together. This holds true for AstC-AstCC, neuroparsin 2-4, Bursicons, tachykinin-related peptides – natalisin, the insulin-like peptides and the glycoprotein hormones. Moreover, prepropeptide genes seem to be unevenly distributed throughout the scaffolds, and may have a tendency to cluster at specific regions. For example, out of the 18 prepropeptides located on the largest ten scaffolds, 15 are located on either scaffold 3, 6, 8 and 9.

Peptide GPCR genes

For most of the predicted peptides, a GPCR was found as suggested by a sequence homology/phylogenetic tree analysis (Supplementary Data 17, Supplementary Fig. 36). This supports the occurrence of the predicted peptidergic signaling pathways. Noteworthy, *C. lectularius* seems to possess two different receptors each for CCAP, CRF-like diuretic hormone, sulfakinin and SIFamide. The functional importance of this finding remains unclear.

For quite a number of peptides, we were unable to identify a receptor. Though this may indicate the absence of these receptors in *C. lectularius*, it is more likely that these GPCRs have been overlooked and will become identifiable with increasing genome coverage. It is also conceivable that we failed to assign identified orphan receptors to identified peptide ligands. Noteworthy, besides the orphan receptors, there is only one identified receptor for which we did not find a ligand: the receptor homolog of the *Drosophila* trissin receptor CG34381[96]. Trissin peptides contain six Cys residues which form three intramolecular disulfide bridges. They where found in Diptera and Lepidoptera so far, but not in other insect taxa even though a rather similar peptide has been predicted in bees (see Caers et al.[107]). We were also unable to detect a homologous peptide in the bed bug.

Biogenic amine GPCR genes

We identified the expected set of GPCRs for octopamine, tyramine, dopamine, serotonin (5-HT) and acetylcholine, as well as some orphan "trace-amine" receptors (Supplementary Data 18, Supplementary Fig. 37[108]). This set is complete when compared to the honey bee and fruit fly[108], with the exception of a dedicated tyramine receptor (CG7431 and CG16766 in *Drosophila*, Am13 in the honeybee). As discussed for the peptide GPCRs above, this may not necessarily indicate the absence of this receptor type in *C. lectularius*.

From a more general perspective, the predicted peptidome and receptor repertoire of *Cimex* shows little peculiarities. This is expected, since all characterized insect peptidomes (with exception of the small parasitoid *Nasonia*[109]) are very similar to each other. In fact, most insect neuropeptide families also occur in the other arthropod

orders[110,111], and there is good evidence that not only aminergic but also many peptidergic signaling pathways are of ancient bilaterian origin[112].

In conclusion, we were able to identify the majority of genes for prepropeptides as well as peptide and amine GPCRs in the bed bug genome. Though most likely correct, the predicted peptide sequences need to be confirmed biochemically as it is not possible to predict posttranslational processing with absolute certainty. The availability of the bed bug genome and our prepropeptide gene annotation now provides a solid platform allowing and greatly facilitating an in-depth biochemical peptidomic characterization. Similarly, the inferred receptor specificities are likely to be correct in most cases, but also need to be tested e.g. by receptor expression in heterologous cell systems. Already now, the annotation of peptide and amine signaling genes opens the door to experimentally dissect the functions of peptides and biogenic amines in the regulation of physiological and developmental processes such as feeding, ecdysis, reproduction and diuresis by RNAi (e.g. Mamidala et al.[113]). It also allows to look for peptide and receptor expression profiles in time and cellular/tissue distribution pattern by PCR and *in-situ* hybridization and informs about the specificity of immunolabelings. Not the least, our analysis especially of the GPCRs can be informative for the development of new insecticides that help to control pyrethroid-resistant bed bug strains[114].

SUPPLEMENTARY NOTE 15

Odorant-binding proteins (OBP) and chemosensory proteins (CSP)

Odorant-binding proteins (OBP) and chemosensory proteins (CSP) were annotated: 11 OBP-coding genes and 16 CSP-coding genes were found, four of which are partial. There are fewer OBP genes in the bed bug genome than in other blood sucking insects such as mosquitoes or tsetse flies but higher than in the black-legged tick *Ixodes scapularis*. The number of CSP genes is lower than in mosquitoes *Aedes aegypti* (Aaeg) and *Culex quinquefasciatus* (Cqui) and higher than in the tsetse fly *Glossina morsitans* (Gmm) and mostly like those of the tick, *I. scapularis* and the human lice *Pediculus humanus*. There are gene duplications in both OBP and CSP gene families. Bed bug OBP genes form no cluster with those of any other insects and seem to be species-specific. On other hand, bed bug CSP genes are more conserved across blood sucking insects with homologous genes in all three mosquito species (Cqui, Agam and Aaeg).

SUPPLEMENTARY NOTE 16

Development and reproduction

Sex-lethal (Sxl) is the master regulator of sex determination cascade in *Drosophila melanogaster*. The SXL protein contains RNA Recognition Motif (RRM domain). Sxl is present in many other insect species. The homolog of Sxl has been identified in the *Cimex* genome and the pre-mRNA likely generates multiple alternatively spliced isoforms in male and females. Transformer (tra) has been identified in most insect species examined. The Tra protein contains Arg/Ser-rich domain. Sex-specific alternative splice forms of *Cimex*-tra have been identified in the genome. Doublesex (dsx) is the most downstream gene of the sex determination cascade. The DSX protein contains a DM (DNA binding domain) domain and OD (oligomerization domain). The pre-mRNA of dsx sex-specifically splices in a manner to generate sex-specific DSX proteins which are similar at the N-terminal regions but differ at their C-terminal (within the c-terminal region of OD domain) regions. Dsx genes and male- and female-specific isoforms have been identified in the genome. Transformer-2 (tra-2) is a partner gene of tra and has a characteristic domain structure consisting of an N-terminal RRM type RNA binding domain (involved in RNA binding) and a C-terminal serine-arginine (SR) domain involved in protein-protein interaction. A tra-2 orthologue has been identified in *Cimex* which could

produce multiple splice forms. Intersex (ix) is required for female sexual development in *Drosophila* as it acts in concert with DSXF at the terminal stages of the sex determination pathway. A male- and a female-specific transcript of ix orthologue have been identified from the *Cimex* genome. Fruitless (fru) is pre-mRNA spliced to produce multiple male- and non sex-specific mRNAs. fru codes for BTB domain containing Zn finger proteins which are responsible for male-specific courtship behavior in *Drosophila*. Fru is relatively less characterized gene outside dipterans. Multiple sex and non-sex specific splice forms of fru orthologue have been identified in *Cimex*.

Cimex goes through five nymphal stages prior to undergoing metamorphosis to the adult stage. Both nymphs and adults feed on human blood and each nymphal stage molts to the next stage only after taking a blood meal. Similarly, the final stage nymph undergoes metamorphosis to the adult stage only after taking a blood meal. Molting and metamorphosis are regulated by hormones including ecdysteriods and juvenile hormones. While ecdysteroids regulate molting, juvenile hormone prevents metamorphosis. Genes coding for ecysteroid biosynthesis (Halloween genes) and juvenile hormone biosynthesis including farnesyl pyrophosphate synthase, mevalonate kinase, phosphomevalonate kinase, diphosphomevalonate decarboxylase, acetoacetyl-CoA thiolase or acetyl-CoA C-acetyltransferase, Isopentenyl diphosphate isomerase or Isopentenyl-diphosphate delta isomerase, HMGCo-A synthase, 3-hydroxy-3-methylglutaryl coenzyme A synthase, HMG-CoA reductase, and 3-hydroxy-3-methylglutaryl-coenzyme A reductase have been identified in the *Cimex* genome. Genes coding for proteins known to be involved in ecdysone action including ecdysone receptor, ultraspiracle, hormone receptor 3, hormone receptor 4, E75, sevenup have been identified. Similarly genes coding for proteins involved in juvenile hormone action including its receptor methoprene tolerant, kr-h1, hairy and steroid receptor co-activator have been identified. These data suggest that *Cimex* employs ecdysteriods and juvenile hormone to regulate development. Nuclear receptors play important roles in regulation of development. Most nuclear receptors identified in *Drosophila* and *Tribolium* have homologs in the *Cimex* genome. The major difference is the absence of HR83 and ERR homologs in the *Cimex* genome. Two Knirps-like genes have been identified in *Cimex* compared to one present in *Tribolium*.

Vitellogenin is a central protein in female reproduction; three genes coding for vitellogenin have been identified in *Cimex*. One gene coding for vitellogenin receptor has been identified. In *Cimex* oocyte maturation occurs only after feeding and matting. Insulin-like peptides likely transduce nutritional signals. Two insulin-like peptides, insulin receptor, terminal transcription factor, FOXO and mTor homologues have been identified in the genome of *Cimex*.

SUPPLEMENTARY NOTE 17

Genome size determination

The colony of bed bugs used in this study originated from Columbus, Ohio in 2002, and has been maintained since then at 85% RH, 15 h:9 h light:dark, 22 °C to promote colony longevity[126]. Blood feeding was as described in Montes et al.[116]. Briefly, bed bug colonies were held within glass Mason jars (1 pint) on folded filter paper (10 cm diameter). Individuals were fed on chicken blood two times a week through a membrane (Parafilm M, Pechiney Plastic, Menasha, WI) that was maintained at 37°C with a circulating water bath. Females and males utilized in this study were two weeks post-eclosion.

Genome size was determined after Johnston et al.[117]. A single head of *C. lectularius* was placed into 1 ml of Galbraith buffer in a 2 ml Kontes Dounce homogenizer along with a single head of a *Drosophila virilis* standard strain, whose genome is 333 Mb by comparison against *D. melanogaster*[118]. Nuclei from the jointly prepared sample and standard were released by grinding with 15 strokes of the "A" pestle, filtered through 20 µm nylon filter and stained with 25 ppm propidium iodide for 30 minutes in the dark at 4°C. Relative fluorescence of nuclei from the sample and standard was determined using a BD

FacScan flow cytometer using 15 Mw illumination at 488 nm and a 590 nm long pass filter. DNA content was determined as the ratio of average fluorescence (channel number) of 2C nuclei of *C. lectularius* divided by the relative fluorescence of 2C nuclei of the *D. virilis* standard times 333 mbp. Replicates were produced for 5 males and 5 females of *C. lectularius*. The genome size of males and females was compared using the GLM procedure from SAS (NC).

The 2C nuclei from *C. lectularius* ran well, producing a peak whose average channel number was more than twice that of the 2C nuclei *of D. virilis* (Supplementary Fig. 38). The average genome size for the *C. lectularius* female was 1C = 864.5 ± 1.7 Mb; the average genome size of a male gamete is significantly smaller ($P < 0.01$) at 1C = 823.5 ± 3.7 Mb.

The limited intraspecific genome size variation and significant genome size difference between males and females of *C. lectularius* reported here is consistent with published cytology. A Berkeley strain of *C. lectularius* was determined to have 26 autosomal chromosomes, with a sex chromosome make-up of X_1X_2Y for males and $X_1X_1X_2$ X_2 for females. Strains with supernumerary X chromosomes were reported[119], but this chromosomal variation was primarily observed in European populations. Data for mtDNA, rDNA and chromosome number[119,120] show low levels of variation across the USA.

The bed bug genome, at 1C = 864 Mb is almost 8 times larger than that of the smallest fully sequenced arthropod (body louse 1C = 104 Mb[117]), yet is well within the range of other complete sequencing projects, being roughly 3/4 the size of *Aedes aegypti* (1C = 1120 Mb) and 1/3 the genome size of *Ixodes scapularius* (1C = 2262 Mb[121]). Establishing an accurate genome size for *Cimex lectularius* L. is essential in furthering molecular research on the bed bug.

SUPPLEMENTARY NOTE 18

Cimex lectularius sialogenome

Saliva of blood sucking animals contains a complex cocktail that disarms their hosts' hemostasis, the physiological process that prevents blood loss consisting of platelet aggregation, vascular responses and blood clotting. A previous bed bug sialotranscriptome (from the Greek sialo=saliva) followed by proteome analysis unraveled its complexity[122]. Several enzymes were found, including a previously described novel apyrase[123] that hydrolyses ADP and ATP agonists of platelet and neutrophil aggregation, diadenosine phosphatases that might hydrolyze nucleotides released by platelets, serine proteases that might be involved in fibrinolysis, esterases similar to acetylcholine esterase, as well as inositol phosphate phosphatases. Salivary serpins may account for the anti-clotting function found in *Cimex* saliva[124]. Small molecule binding proteins include the heme containing nitrophorins which transport nitric oxide, themselves members of the inositol phosphatase family, and salivary odorant binding proteins, with unknown properties. Products belonging to the antigen 5 family, ubiquitously found in

sialotranscriptomes, were also found, as well as several other secreted products of unknown functions.

Expanded gene families (usually found as tandem gene repeats) recruited to a blood sucking salivary function or the presence of single gene duplication events where one product is co-opted for salivary functions are commonly found. The current assembly of the bed bug genome provides insight into the evolutionary processes leading to the unique adaptations necessary for a blood-sucking mode of life. Supplementary Fig. 39 indicates the number of genes found in the bed bug genome for particular gene classes, compared to other genomes. The *Cimex* type apyrase protein was originally discovered in the bed bug salivary glands and shown to be ubiquitously distributed where it plays a cellular role possibly in driving glycosylation reactions. As an example of convergent evolution, this type of enzyme was co-opted as the salivary apyrase in *Rhodnius* (based on the unique calcium-dependence of this type of enzyme[125]) and sand flies[126], while a modified 5'-nucleotidase was co-opted in mosquitoes[127] and in *Triatoma infestans*[128]. All invertebrate genomes scanned in Supplementary Fig. 39 have a single copy of this gene family, except for *Cimex* (2 copies), *Rhodnius* (3 copies), and sand flies (3-7 copies), all of which co-opted this type of enzyme as salivary apyrases. The inositol polyphosphate phosphatase (IPPase) family is also expanded in *Cimex*, with 12 representatives, most on scaffold 13. Similarly, *Cimex* has 6 members of the Ap4A_hydrolase family of diadenosine tetraphosphate hydrolase, the largest number verified in the scanned genomes (Fig. 2; Supplementary Fig. 39). Three of these genes occur in tandem at Scaffold 36. The disclosure of the bed bug genome will help to identify its full sialome by providing a protein database to which proteomic approaches may be used.

<u>Supplemental:</u>

The genomic searches for gene motifs were done with Rpsblast[129] using the Conserved Domain Database (CDD) from the National Institute of Biotechnology Information (NCBI)[130], or from the PFAM database[131] as follows:

Cimex apyrase: gnl|CDD|191443 pfam06079, Apyrase, Apyrase. This family consists of several eukaryotic apyrase proteins (EC:3.6.1.5). The salivary apyrases of blood-feeding arthropods are nucleotide hydrolyzing enzymes implicated in the inhibition

of host platelet aggregation through the hydrolysis of extracellular adenosine diphosphate. Threshold eval = 1e-20.

Inositol phosphate phosphatase: gnl|CDD|197308 cd09074, INPP5c, Catalytic domain of inositol polyphosphate 5-phosphatases.

Inositol polyphosphate 5-phosphatases (5-phosphatases) are signal-modifying enzymes, which hydrolyze the 5-phosphate from the inositol ring of specific 5-position phosphorylated phosphoinositides (PIs) and inositol phosphates (IPs), such as PI(4,5)P2,PI(3,4,5)P3, PI(3,5)P2, I(1,4,5)P3, and I(1,3,4,5)P4. Threshold leval = 1e-20.

Ap4 hydrolases: gnl|CDD|239520 cd03428, Ap4A_hydrolase_human_like, diadenosine tetraphosphate. (Ap4A) hydrolase is a member of the Nudix hydrolase superfamily. Threshold level = 1e-9.

SUPPLEMENTARY NOTE 19

Vitamin metabolism

A comparison of known vitamin metabolism genes from *D*. *melanogaster* and the scaffolds available for *C*. *lectularius* revealed high levels of similarity between these two species and other insects (Supplementary Data 19). Protein sequences from *D*. *melanogaster* were blasted against transcripts from *C*. *lectularius* to find the highest e-value match, and these transcripts were then blasted against the *C*. *lectularius* genome (scaffolds) to find their location (scaffold #). The identified genes were then blasted against databases for *Anopheles*, *Pediculus*, *Tribolium*, and *Pediculus* to find specific orthologs in those species. Of 83 genes studied, only 5 did not have a close ortholog in *C*. *lectularius* (CG7560, CG32099, CG8446, CG12237, CG10581). Several genes that are unique in *D*. *melanogaster* in related areas (e.g. folate production) mapped to a single gene in *C*. *lectularius*, implicating duplication events in *D. melanogaster*. No genes were identified that were unique to only *D*. *melanogaster* and *C*. *lectularius*.

SUPPLEMENTARY NOTE 20

Cimex lectularius immune response analysis

The predicted protein set was queried using a recently curated set of insect immune proteins[132], via BLASTP and high stringency (e 10^-7). Hits were then matched reciprocally to this gene set, and aligned to determine protein completeness. Several problematic families (e.g., CLIP serine proteases and scavenger receptor proteins, which contain CLIP domains) are included with best estimates of naming, but will require additional alignments as well as protein evidence for clarity. Antimicrobial peptides were identified by querying *Cimex* proteins with a predicted length of 100 a.a. and smaller using PSI-BLAST. Query sequences included all AMP's found in paurometabolous insects and exemplars from each of the other insect orders (n = 113 query sequences). Sequences which found at least one match (defensins and diptericins) were then used to

query the *Cimex* genome assembly to identify any proteins that were not included as gene models (none were found).

Additional details

1) Two solid defensin paralogs arose from direct searches of the gene set:
Nominally they should be named as:
Defensin1 - CLEC002659-PA
Defensin2 - CLEC002658-PA

2) There was an interesting cluster of diptericin-related antimicrobial peptides, also close to ‘Prolixin’ from *Rhodnius*. These have consecutive Gene ID numbers CLEC003672-PA, CLEC003673-PA, and CLEC003674-PA, and they will need more alignment to resolve naming. CLEC003672-PA and CLEC003674-PA are 154 and 164 a.a., respectively and indeed seem to have two tandem diptericin components each. CLEC003673-PA is 70 a.a. and is an intact diptericin. These would be interesting to see on a browser, and with expression data, there seem to be at least 3 peptides, maybe 5, in an array.

3) Searches were carried out using all other described hemipteran antimicrobial peptides:

a. NOT FOUND, Short peptide sequences from Chernyah et al.[133]

b. NOT FOUND, Hemiptericins and Pyrrhocoricin found in earlier work starting with Cociancich et al.[134].

c. A secreted protein (CL-4-43-8) cited by Francischetti et al.[122] was present in the genome and showed several orthologs (CLEC011120-PA,CLEC011121-PA,CLEC011118-PA,CLEC011119-PA,CLEC002879-PA,CLEC002878-PA,CLEC002877-PA,CLEC013260-PA). None of these matched known immune effectors

All searches were repeated using PSI-BLAST and a protein dataset pruned to include only models 100 a.a. and smaller, and no additional matches were found, it will likely take a close analysis of upregulated transcripts or peptides after a challenge/mating to identify the rest.

SUPPLEMENTARY NOTE 21

UDP-glycosyltransferase annotation

The *Cimex lectularius* genome contains 7 UDP-glycosyltransferase (UGT) genes including one partial sequence due to a genomic gap (Supplementary Data 20). There are fewer UGT genes in the bed bug genome not only than in any other phytophagous insects of which genomes have been sequences so far, but also than in other blood sucking insects such as mosquitoes or tsetse flies; but higher than in the human body louse *Pediculus humanus corporis* (4 UGTs). Four UGT genes in the bed bug form a cluster in a genomic location (Scaffold64), sharing common three exons with different first exons probably by

alternative splicing, which suggests domain duplication led to the gene diversification having broad substrate specificity (Supplementary Fig. 40).

SUPPLEMENTARY NOTE 22

Transcription factors

We identified likely transcription factors (TFs) by scanning the amino acid sequences of predicted protein coding genes for putative DNA binding domains (DBDs), and when possible, we predicted the DNA binding specificity of each TF using the procedures described in Weirauch et al.[135]. Briefly, we scanned all protein sequences for putative DBDs using the 81 Pfam[136] models listed in Weirauch and Hughes[137] and the HMMER tool[138], with the recommended detection thresholds of Per-sequence E value < 0.01 and Per-domain conditional E value < 0.01. Each protein was classified into a family based on

its DBDs and their order in the protein sequence (e.g., bZIPx1, AP2x2, Homeodomain+Pou). We then aligned the resulting DBD sequences within each family using clustalOmega[139], with default settings. For protein pairs with multiple DBDs, each DBD was aligned separately. From these alignments, we calculated the sequence identity of all DBD sequence pairs (i.e. the percent of AA residues that are exactly the same across all positions in the alignment). Using previously established sequence identify thresholds for each family[135], we mapped the predicted DNA binding specificities by simple transfer. For example, the DBD of CLEC000015-PA is 95% identical to the *Drosophila melanogaster* Awh protein. Since the DNA binding specificity of Awh has already been experimentally determined, and the cutoff for homeodomain TFs is 70%, we can infer that CLEC000015-PA will have the same binding specificity as Awh.

Using the above procedure, we identified a total of 634 putative TFs in the *C. lectularius* genome. This value is similar to counts for other insects (e.g., 640, 551, and 689 for *Apis mellifera*, *Pediculus humanus*, and *Nasonia vitripennis*, respectively). Likewise, for the most part, the number of members of each TF family is comparable to that of other insects (Supplementary Fig. 41), with some notable exceptions. For example, the "MADF" family consists of 29 proteins in *C. lectularius*, which is more than double the number that is present in the genomes of the related insects *A. mellifera* (11 members) and *P. humanus* (1 member). Conversely, the chromatin reorganizing family "BAF1_ABF1" is not present in the *C. lectularius* genome, despite being nearly ubiquitously present in other insect genomes, including up to 18 members in drosophilids.

Of the 634 *C. lectularius* TFs, we were able to infer motifs for 214 (34%) (Supplementary Data 31-32), mostly based on DNA binding specificity data from *D. melanogaster* (133 TFs), but also from species as distant as human (54 TFs) and mouse (12 TFs). Many of the largest TF families have inferred motifs for a substantial proportion of their TFs, including Homeodomain (63 of 81, 78%), bHLH (43 of 52, 83%), and Forkhead box (17 of 19, 89%). As expected, the largest gap is for C2H2 zinc fingers (only 25 of 221, 11%), which evolve quickly by shuffling their many zinc finger arrays, resulting in largely dissimilar DBD sequences across organisms[140].

Supplementary References

1. Benton, R., Vannice, K.S., Gomez-Diaz, C. & Vosshall, L.B. Variant ionotropic glutamate receptors as chemosensory receptors in *Drosophila*. *Cell* **136,** 149-162 (2009).

2. Obiero, G.F. *et al.* Odorant and gustatory receptors in the tsetse fly *Glossina morsitans morsitans*. *PLoS Negl. Trop. Dis.* **8,** e2663 (2014).

3. Willis, J.H. Structural cuticular proteins from arthropods: annotation, nomenclature, and sequence characteristics in the genomics era. *Insect Biochem. Mol. Biol.* **40,** 189-204 (2010).

4. Karouzou, M.V. *et al. Drosophila* cuticular proteins with the R&R Consensus: annotation and classification with a new tool for discriminating RR-1 and RR-2 sequences. *Insect Biochem. Mol. Biol.* **37,** 754-760 (2007).

5. Tamura, K. *et al.* MEGA5: molecular evolutionary genetics analysis using maximum likelihood, evolutionary distance, and maximum parsimony methods. *Mol. Biol. Evol.* **28,** 2731-2739 (2011).

6. Ronquist, F. & Huelsenbeck, J.P. MrBayes 3: Bayesian phylogenetic inference under mixed models. *Bioinformatics* **19,** 1572-1574 (2003).

7. Huson, D.H. *et al.* Dendroscope: an interactive viewer for large phylogenetic trees. *BMC Bioinformatics* **8,** 460 (2007).

8. Skinner, M.E., Uzilov, A.V., Stein, L.D., Mungall, C.J. & Holmes, I.H. JBrowse: a next-generation genome browser. *Genome Res.* **19,** 1630-1638 (2009).

9. Poelchau, M. *et al.* The i5k Workspace@ NAL-enabling genomic data access, visualization and curation of arthropod genomes. *Nucleic Acids Res.* **43,** D714-D719 (2015).

10. Camacho, C. *et al.* BLAST+: architecture and applications. *BMC Bioinformatics* **10,** 421 (2009).

11. Kumar, S. *et al.* The role of reactive oxygen species on *Plasmodium melanotic* encapsulation in *Anopheles gambiae*. *PNAS* **100,** 14139-14144 (2003).

12. Corona, M. & Robinson, G. Genes of the antioxidant system of the honey bee: annotation and phylogeny. *Insect Mol. Biol.* **15,** 687-701 (2006).

13. Salzberg, S.L. *et al.* Serendipitous discovery of *Wolbachia* genomes in multiple *Drosophila* species. *Genome Biol.* **6,** R23 (2005).

14. Benoit, J.B. *et al.* Emerging roles of aquaporins in relation to the physiology of blood-feeding arthropods. *J. Comp. Physiol. B* **184,** 811-825 (2014).

15. Avis, T.J., Michaud, M. & Tweddell, R.J. Role of lipid composition and lipid peroxidation in the sensitivity of fungal plant pathogens to aluminum chloride and sodium metabisulfite. *Appl. Environ. Microbiol.* **73,** 2820-2824 (2007).

16. Larkin, M.A. *et al.* Clustal W and Clustal X version 2.0. *Bioinformatics* **23,** 2947-2948 (2007).

17. Capella-Gutiérrez, S., Silla-Martínez, J.M. & Gabaldón, T. trimAl: a tool for automated alignment trimming in large-scale phylogenetic analyses. *Bioinformatics* **25,** 972-1973 (2009).

18. Guindon, S. *et al.* New algorithms and methods to estimate maximum-likelihood phylogenies: assessing the performance of PhyML 3.0. *Syst. Biol.* **59,** 307-321 (2010).

19. Su, C.Y., Menuz, K. & Carlson, J.R. Olfactory perception: receptors, cells, and circuits. *Cell* **139,** 45-59 (2009).

20. Touhara, K. & Vosshall, L.B. Sensing odorants and pheromones with chemosensory receptors. *Annu. Rev. Physiol.* **71,** 307-332 (2009).

21. Jones, W.D., Cayirlioglu, P., Kadow, I.G. & Vosshall, L.B. Two chemosensory receptors together mediate carbon dioxide detection in *Drosophila*. *Nature* **445,** 86-90 (2007).

22. Kwon, J.Y., Dahanukar, A., Weiss, L.A. & Carlson, J.R. The molecular basis of CO_2 reception in *Drosophila*. *Proc. Natl. Acad. Sci. USA* **104,** 3574-3578 (2007).

23. Lu, T. *et al.* Odor coding in the maxillary palp of the malaria vector mosquito *Anopheles gambiae*. *Curr. Biol.* **17,** 1533-1544 (2007).

24. Liman, E.R., Zhang, Y.V. & Montell, C. Peripheral coding of taste. *Neuron* **81,** 984-1000 (2014).

25. Benton, R., Vannice, K.S., Gomez-Diaz, C. & Vosshall, L.B. Variant ionotropic glutamate receptors as chemosensory receptors in *Drosophila*. *Cell* **136,** 149-162 (2009).

26. Croset, V. *et al.* Ancient protostome origin of chemosensory ionotropic glutamate receptors and the evolution of insect taste and olfaction. *PLoS Genet.* **6,** e1001064 (2010).

27. Abuin, L. *et al.* Functional architecture of olfactory ionotropic glutamate receptors. *Neuron* **69,** 44-60 (2011).

28. Rytz, R., Croset, V. & Benton, R. Ionotropic receptors (IRs): chemosensory ionotropic glutamate receptors in *Drosophila* and beyond. *Insect Biochem. Mol. Biol.* **43,** 888-897 (2013).

29. Kirkness, E.F. *et al.* Genome sequences of the human body louse and its primary endosymbiont provide insights into the permanent parasitic lifestyle. *Proc. Natl. Acad. Sci. USA* **107,** 12168-12173 (2010).

30. Smith, C.R. *et al.* Draft genome of the red harvester ant *Pogonomyrmex barbatus*. *Proc. Natl. Acad. Sci. USA* **108,** 5667-5672 (2011).

31. Smadja, C., Shi, P., Butlin, R.K. & Robertson, H.M. Large gene family expansions and adaptive evolution for odorant and gustatory receptors in the pea aphid, *Acyrthosiphon pisum*. *Mol. Biol. Evol.* **26,** 2073-2086 (2009).

32. Robertson, H.M., Warr, C.G. & Carlson, J.R. Molecular evolution of the insect chemoreceptor gene superfamily in *Drosophila melanogaster*. *Proc. Natl. Acad. Sci. USA* **100,** 14537-14542 (2003).

33. Robertson, H.M. & Wanner, K.W. The chemoreceptor superfamily in the honey bee, *Apis mellifera*: expansion of the odorant, but not gustatory, receptor family. *Genome Res.* **16,** 1395-1403 (2006).

34. Smadja, C.M. *et al.* Large-scale candidate gene scan reveals the role of the chemoreceptor genes in host plant specialization and speciation in the pea aphid. *Evolution* **66,** 2723-2738 (2012).

35. Vosshall, L.B. & Hansson, B.S. A unified nomenclature system for the insect olfactory coreceptor. *Chem. senses* **36,** 497-498 (2011).

36. Richards, S. *et al.* The genome of the model beetle and pest *Tribolium castaneum*. *Nature* **452,** 949-955 (2008).

37. Missbach, C. *et al.* Evolution of insect olfactory receptors. *Elife* **3,** e02115 (2014).

38. Peñalva-Arana, D.C., Lynch, M. & Robertson, H.M. The chemoreceptor genes of the waterflea *Daphnia pulex*: many Grs but no Ors. *BMC Evol. Biol.* **9**, 79 (2009).

39. Chipman, A.D. *et al.* The first myriapod genome sequence reveals conservative arthropod gene content and genome organisation in the centipede *Strigamia maritima*. *PLoS Biol*.**12,** e1002005 (2014).

40. Saina, M. *et al.* A cnidarian homologue of an insect gustatory receptor functions in developmental body patterning. *Nat. Commun.* **6,** doi:10.1038 (2015).

41. Koh, T.W. *et al.* The *Drosophila* IR20a clade of ionotropic receptors are candidate taste and pheromone receptors. *Neuron* **83,** 850-865 (2014).

42. Stewart, S., Koh, T.W., Ghosh, A.C. & Carlson, J.R. Candidate ionotropic taste receptors in the *Drosophila* larva. *Proc. Natl. Acad. Sci. USA* **112,** 4195-4201 (2015).

43. Xiao, J.H. *et al.* Obligate mutualism within a host drives the extreme specialization of a fig wasp genome. *Genome Biol*. **14,** R141 (2013).

44. Terrapon, N. *et al.* Molecular traces of alternative social organization in a termite genome. *Nat. Commun.* **5** (2014).

45. Miyamoto, T., Slone, J., Song, X. & Amrein, H. A fructose receptor functions as a nutrient sensor in the *Drosophila* brain. *Cell* **151,** 1113-1125 (2012).

46. Sato, K., Tanaka, K. & Touhara, K. Sugar-regulated cation channel formed by an insect gustatory receptor. *Proc. Natl. Acad. Sci. USA* **108,** 11680-11685 (2011).

47. Grosjean, Y. *et al.* An olfactory receptor for food-derived odours promotes male courtship in *Drosophila*. *Nature* **478,** 236-240 (2011).

48. Peschel, N. & Helfrich-Förster, C. Setting the clock-by nature: circadian rhythm in the fruitfly *Drosophila melanogaster*. *FEBS Lett.* **585,** 1435-1442 (2011).

49. Bloch, G. The social clock of the honeybee. *J. Biol. Rhythms* **25,** 307-317 (2010).

50. Rubin, E.B. *et al.* Molecular and phylogenetic analyses reveal mammalian-like clockwork in the honey bee (*Apis mellifera*) and shed new light on the molecular evolution of the circadian clock. *Genome Res.* **16,** 1352-1365 (2006).

51. Yuan, Q., Metterville, D., Briscoe, A.D. & Reppert, S.M. Insect cryptochromes: gene duplication and loss define diverse ways to construct insect circadian clocks. *Mol. Biol. Evol.* **24,** 948-955 (2007).

52. Darlington, T.K. *et al.* Closing the circadian loop: CLOCK-induced transcription of its own inhibitors per and tim. *Science* **280,** 1599-1603 (1998).

53. Lee, C., Bae, K. & Edery, I. PER and TIM inhibit the DNA binding activity of a *Drosophila* CLOCK/dBMAL1 heterodimer without disrupting formation of the heterodimer: a basis for circadian transcription. *Mol. Cell Biol.* **19,** 5316-5325 (1999).

54. Reppert, S.M. The ancestral circadian clock of monarch butterflies: role in time-compensated sun compass orientation. *Cold Spring Harb. Symp. Quant. Biol.* **72,** 113 -118 (2007).

55. Ousley, P. & Terry, M. Screening of donor eyes for prior PRK: evaluation of the Orbscan and TMS-1 technologies. *Invest. Ophthalmol. Vis. Sci.* **38,** S848 (1997).

56. Tomita, T. *et al.* Conserved amino acid residues in C-terminus of PERIOD 2 are involved in interaction with CRYPTOCHROME 1. *Biochim. Biophys. Acta* **1803,** 492-498 (2010).

57. Vaidya, A.T. *et al.* Flavin reduction activates *Drosophila* cryptochrome. *Proc. Natl. Acad. Sci. USA* **110,** 20455-20460 (2013).

58. Emery, P. *et al.* *Drosophila* CRY is a deep brain circadian photoreceptor. *Neuron* **26,** 493-504 (2000).

59. Koh, K., Zheng, X. & Sehgal, A. JETLAG resets the *Drosophila* circadian clock by promoting light-induced degradation of TIMELESS. *Science* **312,** 1809-1812 (2006).

60. Hoang, N. *et al.* Human and *Drosophila* cryptochromes are light activated by flavin photoreduction in living cells. *PLoS Biol.* **6,** e160 (2008).

61. Koganemaru, R., Miller, D.M. & Adelman, Z.N. Robust cuticular penetration resistance in the common bed bug (*Cimex lectularius* L.) correlates with increased steady-state transcript levels of CPR-type cuticle protein genes. *Pest. Biochem. Physiol.* **106,** 190-197 (2013).

62. Mamidala, P. *et al.* RNA-Seq and molecular docking reveal multi-level pesticide resistance in the bed bug. *BMC Genomics* **13,** 6 (2012).

63. Mamidala, P., Jones, S.C. & Mittapalli, O. Metabolic resistance in bed bugs. *Insects* **2,** 36-48 (2012).

64. Zhu, F. *et al.* Bed bugs evolved unique adaptive strategy to resist pyrethroid insecticides. *Sci. Rep.* **3,** 1456 (2013).

65. Willis, J.H. Structural cuticular proteins from arthropods: annotation, nomenclature, and sequence characteristics in the genomics era. *Insect Biochem. Mol. Biol.* **40,** 189-204 (2010).

66. Karouzou, M.V. *et al. Drosophila* cuticular proteins with the R&R Consensus: annotation and classification with a new tool for discriminating RR-1 and RR-2 sequences. *Insect Biochem. Mol. Biol.* **37,** 754-760 (2007).

67. Futahashi, R. *et al.* Genome-wide identification of cuticular protein genes in the silkworm, *Bombyx mori*. *Insect Biochem. Mol. Biol.* **38,** 1138-1146 (2008).

68. Willis, J.H., Papandreou, N.C., Iconomidou, V.A. & Hamodrakas, S.J. Cuticular proteins. in *Insect Mol. Biol. Biochem.*134-166 (Academic Press San Diego, 2012).

69. Vannini, L., Dunn, W.A., Reed, T.W. & Willis, J.H. Changes in transcript abundance for cuticular proteins and other genes three hours after a blood meal in *Anopheles gambiae*. *Insect Biochem. Mol. Biol.* **44,** 33-43 (2014).

70. Nakato, H., Toriyama, M., Izumi, S. & Tomino, S. Structure and expression of mRNA for a pupal cuticle protein of the silkworm, *Bombyx mori. Insect Biochem.* **20,** 667-678 (1990).

71. Andersen, S.O., Rafn, K. & Roepstorff, P. Sequence studies of proteins from larval and pupal cuticle of the yellow meal worm, *Tenebrio molitor*. *Insect Biochem. Mol. Biol.* **27,** 121-131 (1997).

72. Bao, Y.Y. *et al.* Genomic insights into the serine protease gene family and expression profile analysis in the planthopper, *Nilaparvata lugens. BMC Genomics* **15,** 507 (2014).

73. Ribeiro, J.M. *et al.* An insight into the transcriptome of the digestive tract of the bloodsucking bug, *Rhodnius prolixus. PLoS Neglect. Trop. Dis.* **8,** e2594 (2014).

74. Cristofoletti, P.T., Ribeiro, A.F., Deraison, C., Rahbé, Y. & Terra, W.R. Midgut adaptation and digestive enzyme distribution in a phloem feeding insect, the pea aphid *Acyrthosiphon pisum. J. Insect Physiol.* **49,** 11-24 (2003).

75. Rider Jr, S., Srinivasan, D. & Hilgarth, R. Chromatin-remodelling proteins of the pea aphid, *Acyrthosiphon pisum* (Harris). *Insect Mol. Biol.* **19,** 201-214 (2010).

76. Balvín, O., Munclinger, P., Kratochvíl, L. & Vilímová, J. Mitochondrial DNA and morphology show independent evolutionary histories of bedbug *Cimex lectularius* (Heteroptera: Cimicidae) on bats and humans. *Parasit. Res.* **111,** 457-469 (2012).

77. Singh, R.N., Singh, K., Prakash, S., Mendki, M. & Rao, K. Sensory organs on the body parts of the bed-bug *Cimex hemipterus* Fabricius (Hemiptera: Cimicidae) and the anatomy of its central nervous system. *Int. J. Insect Morph.* **25,** 183-204 (1996).

78. Singh, N., Wang, C. & Cooper, R. Role of vision and mechanoreception in bed bug, *Cimex lectularius* L. behavior. *PLoS One* **10,** e0118855 (2015).

79. Malagoli, D. *et al.* Autophagy and its physiological relevance in arthropods: current knowledge and perspectives. *Autophagy* **6,** 575-588 (2010).

80. Benoit, J.B., Lopez-Martinez, G., Phillips, Z.P., Patrick, K.R. & Denlinger, D.L. Heat shock proteins contribute to mosquito dehydration tolerance. *J. Insect Physiol.* **56,** 151-156 (2010).

81. Krumlauf, R. Evolution of the vertebrate Hox homeobox genes. *BioEssays* **14,** 245-252 (1992).

82. Brown, S.J. *et al.* Sequence of the *Tribolium castaneum* homeotic complex: the region corresponding to the *Drosophila melanogaster* antennapedia complex. *Genetics* **160,** 1067-1074 (2002).

83. *Tribolium* Genome Sequencing Consortium. The genome of the model beetle and pest *Tribolium castaneum*. *Nature* **452,** 949-955 (2008).

84. Adams, M.D. *et al.* The genome sequence of *Drosophila melanogaster*. *Science* **287,** 2185-2195 (2000).

85. Negre, B. *et al.* Conservation of regulatory sequences and gene expression patterns in the disintegrating *Drosophila* Hox gene complex. *Genome Res.* **15,** 692-700 (2005).

86. Panfilio, K.A. & Akam, M. A comparison of Hox3 and Zen protein coding sequences in taxa that span the Hox3/zen divergence. *Dev. Genes Evol.* **217,** 323-329 (2007).

87. Panfilio, K.A., Liu, P.Z., Akam, M. & Kaufman, T.C. *Oncopeltus fasciatus zen* is essential for serosal tissue function in katatrepsis. *Dev. Biol.* **292,** 226-243 (2006).

88. Gómez-Skarmeta, J.L., del Corral, R.D., de la Calle-Mustienes, E., Ferrés-Marcó, D. & Modolell, J. Araucan and caupolican, two members of the novel iroquois complex, encode homeoproteins that control proneural and vein-forming genes. *Cell* **85,** 95-105 (1996).

89. Kehl, B.T., Cho, K.O. & Choi, K.W. Mirror, a *Drosophila* homeobox gene in the iroquois complex, is required for sensory organ and alula formation. *Development* **125,** 1217-1227 (1998).

90. McNeill, H., Yang, C.H., Brodsky, M., Ungos, J. & Simon, M.A. Mirror encodes a novel PBX-class homeoprotein that functions in the definition of the dorsal-ventral border in the *Drosophila* eye. *Genes Dev.* **11,** 1073-1082 (1997).

91. Orchard, I. & Paluzzi, J.P. Diuretic and antidiuretic hormones in the blood-gorging bug *Rhodnius prolixus*. *Ann. NY Acad. Sci.* **1163,** 501-503 (2009).

92. Pauls, D. *et al.* Peptidomics and processing of regulatory peptides in the fruit fly *Drosophila*. *EuPA Open Proteom.* **3,** 114-127 (2014).

93. Gäde, G. & Goldsworthy, G.J. Insect peptide hormones: a selective review of their physiology and potential application for pest control. *Pest Manag. Sci.* **59,** 1063-1075 (2003).

94. Verlinden, H. *et al.* Receptors for neuronal or endocrine signalling molecules as potential targets for the control of insect pests. *Adv. Insect Physiol.* **46,** 167-303 (2014).

95. Veenstra, J.A. Mono- and dibasic proteolytic cleavage sites in insect neuroendocrine peptide precursors. *Arch. Insect Biochem.* **43,** 49-63 (2000).

96. Ida, T. *et al.* Identification of the endogenous cysteine-rich peptide trissin, a ligand for an orphan G protein-coupled receptor in *Drosophila*. *Biochem. Biophys. Res. Co.* **414,** 44-48 (2011).

97. Nygaard, S. *et al.* The genome of the leaf-cutting ant *Acromyrmex echinatior* suggests key adaptations to advanced social life and fungus farming. *Genome Res.* **21,** 1339-1348 (2011).

98. Tanaka, Y., Suetsugu, Y., Yamamoto, K., Noda, H. & Shinoda, T. Transcriptome analysis of neuropeptides and G-protein coupled receptors (GPCRs) for neuropeptides in the brown planthopper *Nilaparvata lugens*. *Peptides* **53,** 125-133 (2014).

99. Huybrechts, J. *et al.* Neuropeptide and neurohormone precursors in the pea aphid, *Acyrthosiphon pisum. Insect Mol. Biol.* **19,** 87-95 (2010).

100. Neupert, S., Russell, W.K., Russell, D.H. & Predel, R. Two capa-genes are expressed in the neuroendocrine system of *Rhodnius prolixus*. *Peptides* **31,** 408-41 (2010).

101. Paluzzi, J.P. & Orchard, I. A second gene encodes the anti-diuretic hormone in the insect, *Rhodnius prolixus*. *Mol. Cell. Endocrinol.* **317,** 53-63 (2010).

102. Lee, D., Taufique, H., da Silva, R. & Lange, A.B. An unusual myosuppressin from the blood-feeding bug *Rhodnius prolixus*. *J. Exp. Biol.* **215,** 2088-2095 (2012).

103. Ons, S., Sterkel, M., Diambra, L., Urlaub, H. & Rivera-Pomar, R. Neuropeptide precursor gene discovery in the Chagas disease vector *Rhodnius prolixus*. *Insect Mol. Biol.* **20,** 29-44 (2011).

104. Predel, R. *et al.* Comparative peptidomics of four related hemipteran species: pyrokinins, myosuppressin, corazonin, adipokinetic hormone, sNPF, and periviscerokinins. *Peptides* **29,** 162-167 (2008).

105. Predel, R., Neupert, S., Russell, W.K., Scheibner, O. & Nachman, R.J. Corazonin in insects. *Peptides* **28,** 3-10 (2007).

106. Settembrini, B.P. *et al.* Distribution and characterization of corazonin in the central nervous system of *Triatoma infestans* (Insecta: Heteroptera). *Peptides* **32,** 461-468 (2011).

107. Caers, J. *et al.* More than two decades of research on insect neuropeptide GPCRs: an overview. *Front. Endocrinol.* **3,** 151 (2012).

108. Hauser, F., Cazzamali, G., Williamson, M., Blenau, W. & Grimmelikhuijzen, C.J. A review of neurohormone GPCRs present in the fruitfly *Drosophila melanogaster* and the honey bee *Apis mellifera. Prog. Neurobiol.* **80,** 1-19 (2006).

109. Hauser, F. *et al.* Genomics and peptidomics of neuropeptides and protein hormones present in the parasitic wasp *Nasonia vitripennis. J. Proteome Res.* **9,** 5296-5310 (2010).

110. Veenstra, J.A., Rombauts, S. & Grbić, M. In silico cloning of genes encoding neuropeptides, neurohormones and their putative G-protein coupled receptors in a spider mite. *Insect Biochem. Mol. Biol.* **42,** 277-295 (2012).

111. Dircksen, H. *et al.* Genomics, transcriptomics, and peptidomics of *Daphnia pulex* neuropeptides and protein hormones. *J. Proteome Res.* **10,** 4478-4504 (2011).

112. Mirabeau, O. & Joly, J.S. Molecular evolution of peptidergic signaling systems in bilaterians. *Proc. Natl. Acad. Sci. USA* **110,** E2028-E2037 (2013).

113. Mamidala, P., Mittapelly, P., Jones, S.C., Piermarini, P.M. & Mittapalli, O. Molecular characterization of genes encoding inward rectifier potassium (Kir) channels in the bed bug (*Cimex lectularius*). *Comp. Biochem. Phys. B* **164,** 275-279 (2013).

114. Davies, T.G., Field, L.M. & Williamson, M.S. The re-emergence of the bed bug as a nuisance pest: implications of resistance to the pyrethroid insecticides. *Med. Vet. Entomol.* **26,** 241-54 (2012).

115. Benoit, J.B., Del Grosso, N.A., Yoder, J.A. & Denlinger, D.L. Resistance to dehydration between bouts of blood feeding in the bed bug, *Cimex lectularius*, is enhanced by water conservation, aggregation, and quiescence. *Am. J. Trop. Med. Hyg.* **76,** 987-993 (2007).

116. Montes, C., Cuadrillero, C. & Vilella, D. Maintenance of a laboratory colony of *Cimex lectularius* (Hemiptera: Cimicidae) using an artificial feeding technique. *J. Med. Entomol.* **39,** 675-679 (2002).

117. Johnston, J.S., Yoon, K.S., Strycharz, J.P., Pittendrigh, B.R. & Clark, J.M. Body lice and head lice (Anoplura: Pediculidae) have the smallest genomes of any hemimetabolous insect reported to date. *J. Med. Entomol.* **44,** 1009-1012 (2007).

118. Gregory, T.R. & Johnston, J.S. Genome size diversity in the family Drosophilidae. *Heredity* **101,** 228-238 (2008).

119. Ueshima, N. Supernumerary chromosomes in the human bed bug, *Cimex lectularius* Linn. (Cimicidae: Hemiptera). *Chromosoma* **20,** 311-331 (1967).

120. Szalanski, A.L., Austin, J.W., McKern, J.A., Steelman, C.D. & Gold, R.E. Mitochondrial and ribosomal internal transcribed spacer 1 diversity of *Cimex lectularius* (Hemiptera: Cimicidae). *J. Med. Entomol.* **45,** 229-236 (2008).

121. Geraci, N.S., Johnston, J.S., Robinson, J.P., Wikel, S.K. & Hill, C.A. Variation in genome size of argasid and ixodid ticks. *Insect Biochem. Mol. Biol.* **37,** 399-408 (2007).

122. Francischetti, I.M. *et al.* Insight into the sialome of the bed bug, *Cimex lectularius. J. Proteome Res.* **9,** 3820-31 (2010).

123. Valenzuela, J.G., Charlab, R., Galperin, M.Y. & Ribeiro, J.M. Purification, cloning, and expression of an apyrase from the bed bug *Cimex lectularius*. A new type of nucleotide-binding enzyme. *J. Biol. Chem.* **273,** 30583-30590 (1998).

124. Valenzuela, J.G., Guimaraes, J.A. & Ribeiro, J.M. A novel inhibitor of factor X activation from the salivary glands of the bed bug *Cimex lectularius. Exp. Parasitol.* **83,** 184-90 (1996).

125. Sarkis, J.J., Guimaraes, J.A. & Ribeiro, J.M. Salivary apyrase of *Rhodnius prolixus*. Kinetics and purification. *Biochem. J.* **233,** 885-891 (1986).

126. Valenzuela, J.G., Belkaid, Y., Rowton, E. & Ribeiro, J.M. The salivary apyrase of the blood-sucking sand fly *Phlebotomus papatasi* belongs to the novel *Cimex* family of apyrases. *J. Exp. Biol.* **204,** 229-237. (2001).

127. Champagne, D.E., Smartt, C.T., Ribeiro, J.M. & James, A.A. The salivary gland-specific apyrase of the mosquito *Aedes aegypti* is a member of the 5'-nucleotidase family. *Proc. Natl. Acad. Sci. USA* **92,** 694-698 (1995).

128. Faudry, E. *et al. Triatoma infestans* apyrases belong to the 5'-nucleotidase family. *J. Biol. Chem.* **279,** 19607-19613 (2004).

129. Altschul, S.F. *et al.* Gapped BLAST and PSI-BLAST: a new generation of protein database search programs. *Nuc. Acids Res.* **25,** 3389-3402 (1997).

130. Tatusov, R.L. *et al.* The COG database: an updated version includes eukaryotes. *BMC Bioinformatics* **4,** 41 (2003).

131. Bateman, A. *et al.* The Pfam protein families database. *Nuc. Acids Res.* **28,** 263-266 (2000).

132. Barribeau, S.M. *et al.* A depauperate immune repertoire precedes evolution of sociality in bees. *Genome Biol.* **16,** 83 (2015).

133. Chernysh, S., Cociancich, S., Briand, J.P., Hetru, C. & Bulet, P. The inducible antibacterial peptides of the hemipteran insect *Palomena prasina*: identification of a unique family of prolinerich peptides and of a novel insect defensin. *J. Insect Physiol.* **42,** 81-89 (1996).

134. Cociancich, S. *et al.* Novel inducible antibacterial peptides from a hemipteran insect, the sap-sucking bug *Pyrrhocoris apterus*. *Biochem. J.* **300,** 567-575 (1994).

135. Weirauch, M.T. *et al.* Determination and inference of eukaryotic transcription factor sequence specificity. *Cell* **158**, 1431-1433 (2014).

136. Finn, R.D. *et al.* The Pfam protein families database. *Nuc. Acids Res.* **38**, D211-D222 (2014).

137. Weirauch, M.T. & Hughes, T.R. A catalogue of eukaryotic transcription factor types, their evolutionary origin, and species distribution. *Subcell. Biochem.* **52**, 25-73 (2011).

138. Eddy, S.R. A new generation of homology search tools based on probabilistic inference. *Genome Inform.* **23**, 205-211 (2009).

139. Sievers, F. *et al.* Fast, scalable generation of high-quality protein multiple sequence alignments using Clustal Omega. *Mol. Syst. Biol.* **7**, 539 (2011).

140. Najafabadi, H.S., *et al.* C2H2 zinc finger proteins greatly expand the human regulatory lexicon. *Nat. Biotechnol.* **33**, 555-562 (2015).
